# Development of Novel Anticancer Pyrazolopyrimidinones Targeting Glioblastoma

**DOI:** 10.1002/cmdc.202500337

**Published:** 2025-08-08

**Authors:** Kate Byrne, Natalia Bednarz, Ciara McEvoy, John C. Stephens, James F. Curtin, Gemma K. Kinsella

**Affiliations:** ^1^ School of Food Science and Environmental Health Technological University Dublin Grangegorman Lower, Dublin 7 Dublin D07 ADY7 Ireland; ^2^ Sustainability and Health Research Hub (SHRH) Technological University Dublin Grangegorman Lower, Dublin 7 Dublin D07 ADY7 Ireland; ^3^ Department of Chemistry Maynooth University Maynooth W23 F2H6 Ireland; ^4^ Kathleen Lonsdale Institute for Human Health Research Maynooth University Maynooth W23 F2H6 Ireland

**Keywords:** cancer, glioblastoma, pyrazolo[1,5‐*α*]pyrimidinones, structure‐activity relationships

## Abstract

Glioblastoma (GBM) is the most common and aggressive malignant grade IV brain tumor and is one of the most difficult types of brain cancer to treat with a high incidence of resistance to traditionally used chemotherapeutics. Pyrazolopyrimidinones are fused nitrogen‐containing heterocyclic systems which are a scaffold in several bioactive drugs and drug candidates. Here, a structure–activity relationship (SAR) study was performed where 23 substituted pyrazolo[1,5‐*α*]pyrimidinones were screened for cytotoxicity against the GBM U‐251 MG cell line and the noncancerous embryonic kidney HEK293 cell line to assess their potential as antiGBM agents capable of selectivity for cancer cells. Through analog synthesis of preliminary HIT compounds with varied structural substituents, a lead compound, **22**, has been identified, which proved capable of inducing significant GBM cell death while having a marginal cytotoxicity against the noncancerous cells. The mode of cell death studies suggested that the structurally varied HIT compounds induced cell death through differential mechanisms including cell membrane permeabilization and mitochondria membrane depolarization‐dependent mechanisms such as necrosis or apoptosis. The results highlight the potential of pyrazolo[1,5‐*α*]pyrimidinones derivatives as a novel anti‐GBM therapy, capable of selectively killing cancer cells. Furthermore, pyrazolo[1,5‐*α*]pyrimidinones provide a scaffold for further development of selective GBM therapies.

## Graphical abstract

Pyrazolo[1,5‐*α*]pyrimidinone derivatives exhibit selective cytotoxicity towards glioblastoma (GBM) cells over noncancerous cells. Structure–activity relationship studies identifies a lead compound with significant cytotoxicity activity, inducing apoptosis and necrosis in GBM cells, without affecting noncancerous cells. This demonstrates the potential of pyrazolopyrimidinones as selective therapeutic agents for GBM.

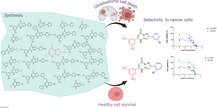

## 1 Introduction

Glioblastoma (GBM), previously known as GBM multiforme, is a common, highly aggressive grade IV isocitrate dehydrogenase (IDH)‐wildtype brain tumor.^[^ ^1^ ^]^ GBM is associated with a poor prognosis, with its 5‐year survival rate being less than 10% and a median survival time of 15 months.^[^ ^2^, ^3^ ^–^ ^4^ ^]^ GBM usually first manifests as a headache and progressively develops with symptoms such as seizures, focal neurological deficits, nausea, vomiting, blurry vision, and altered mental status due to the increase in intracranial pressure.^[^ ^5^ ^]^ The current treatment options for GBM are limited and often include surgical resection, adjuvant to chemotherapy and radiotherapy. Full surgical resection is often not possible due to the invasion of the tumor into functional regions of the brain. Hence, the surgical approach aims to decrease the tumor mass, and radiotherapy and chemotherapy are used to further treat the tumor.^[^ ^6^ ^]^ However, a major challenge in GBM treatment is its high incidence of resistance to radiotherapy and traditionally used chemotherapeutics. Temozolomide (TMZ) is the gold standard for GBM treatment.^[^ ^7^ ^,^ ^8^ ^]^ However, TMZ treatment fails in about 90% of patients and leads to recurrence, as the tumor may already be resistant due to upregulation of methylated‐DNA‐protein‐cysteine methyltransferase (MGMT), a DNA repair enzyme that inhibits TMZ's function, or through acquired tumor resistance post treatment leading to recurrence.^[^ ^8^ ^,^ ^9^ ^]^ Therefore, there is a clear need for novel therapeutic options for GBM.

**Figure 1:**
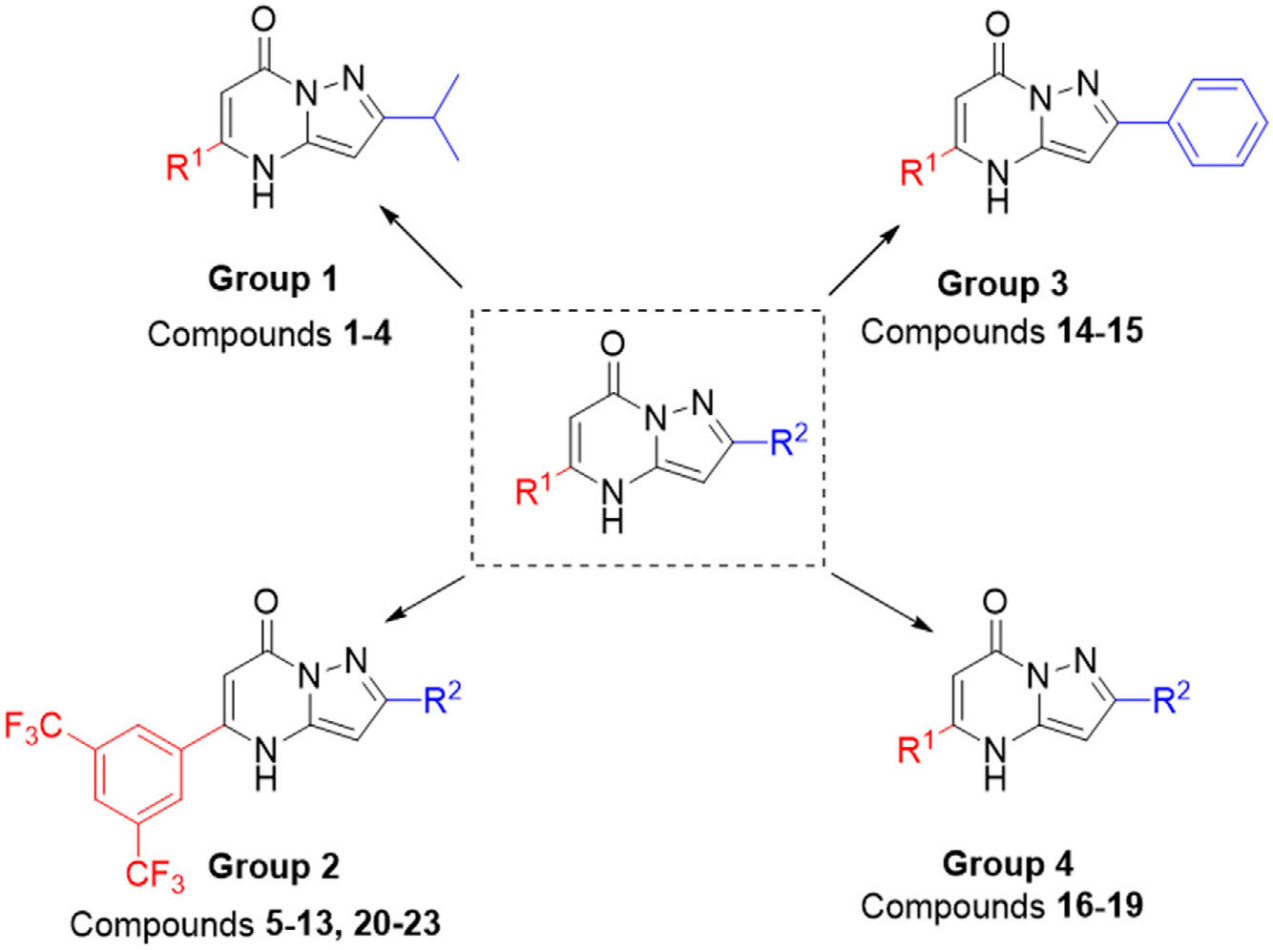
General structure of pyrazolo[1,5‐*α*]pyrimidinones with its groups 1–4.

Pyrazolopyrimidinones are nitrogen‐containing heterocyclic compounds, which act as a core scaffold in many bioactive pharmaceutical compounds.^[^ ^10^ ^,^ ^11^ ^]^ Derivatives of pyrazolopyrimidinones (for an example of the core structure, see **Figure** **1**) have shown inhibitory effects on many enzymes, for instance, several kinases such as PI3 kinase, glycogen synthase kinase‐3 (GSK‐3), and Src kinases, which are involved and play key roles in cellular functions and pathways.^[^ ^12^, ^13^, ^14^, ^15^, ^16^, ^17^, ^18^ ^–^ ^19^ ^]^ Hence, the beneficial use of pyrazolopyrimidinones derivatives has been highlighted for Alzheimer's disease, inflammation, erectile disfunction, obesity, cystic fibrosis, infections, and cancer therapy, among others.^[^ ^11^ ^,^ ^16^ ^,^ ^17^ ^,^ ^20^, ^21^, ^22^ ^–^ ^23^ ^]^ Notably, the anticancer properties of pyrazolopyrimidinone derivatives have been highlighted with their in vitro cytotoxicity against a number of cancer types including breast, GBM, colon, and liver cancer cell lines, with many compounds exhibiting a low half maximal inhibitory concentration (IC_50_) correlating to high cytotoxicity at relatively low concentrations.^[^ ^19^ ^,^ ^24^, ^25^, ^26^ ^–^ ^27^ ^]^ Interestingly, El‐Mekabaty and colleagues (2020) have reported that some pyrazolopyrimidinone derivatives exhibit selectivity towards liver hepatocellular carcinoma (HepG2) cells, with lesser cytotoxicity towards normal lung fibroblasts (WI‐38) cells, highlighting the potential of pyrazolopyrimidinone as a selective therapy which could lead to decreased incidence of adverse effects.^[^ ^26^ ^]^

An additional challenge with GBM treatment is the development of therapeutics that are both orally bioavailable and can cross the blood brain barrier (BBB).^[^ ^28^ ^,^ ^29^ ^]^ Pyrazolo[1,5‐*α*]pyrimidinones derivatives can be synthesized to adhere to Lipinski's rules, to aid oral bioavailability, which has been done by others and in this study.^[^ ^30^ ^,^ ^31^ ^]^ Additionally, pyrazolopyrimidinones are highly lipophilic, and they can be synthesized to have a low size and molecular weight with few rotatable bonds and hydrogen bond donors, which are properties favored for BBB drug permeability.^[^ ^19^ ^,^ ^32^, ^33^ ^–^ ^34^ ^]^ Furthermore, we have previously examined the potential of pyrazolo[1,5‐*α*]pyrimidinones as anticancer agents, by demonstrating that pyrazolo[1,5‐*α*]pyrimidinones derivatives have significant cytotoxicity against GBM cells.^[^ ^19^ ^]^ In the previous study, the compounds also showed potential as a combination therapy with cold atmospheric plasma, which further enhanced the cytotoxicity of certain derivatives, which highlights the potential and versatility of pyrazolopyrimidinones as a novel GBM therapy.

Here, a family of 23 pyrazolo[1,5‐*α*]pyrimidinones were developed and evaluated for their anticancer effects using two cell lines, the cancerous U‐251 MG GBM cell line and the noncancerous, immortalized human embryonic kidney (HEK293) cell line. The family composed of substituted pyrazolo[1,5‐*α*]pyrimidinones with R^1^ substituent on the pyrimidine ring and an R^2^ substituent on the pyrazole ring. The pyrazolo[1,5‐*α*]pyrimidinones were organized into groups based on structural changes made at the R^1^ and R^2^ position, with the core scaffold remaining unchanged (Figure 1). A structure–activity relationship (SAR) study was performed to determine the potential of the compounds in treating GBM, focusing on their cytotoxicity and selectivity toward U‐251 MG cells. Four HIT compounds, **5**, **7**, **9**, and **10**, were progressed which showed high cytotoxicity against the U‐251 MG at values lower than 50 µM (the biological response threshold) and a selective capacity for the cancer cells with the cytotoxicity against HEK293 higher than 50 µM.^[^ ^35^ ^]^ Further optimization of the structures, based on the initial observed HIT compounds, resulted in the development of the lead compound, **22**, which showed significant cytotoxicity against GBM cells at 2.79 µM, while exhibiting significant selective capacity for cancer cells with a lack of a relevant biological response against the noncancerous HEK293 cells. The mode of cell death was investigated using the mitochondrial membrane depolarization JC‐1 dye and cell membrane impermeable and DNA staining propidium iodide (PI) dye which suggested that the compounds induce cell death by cell membrane permeabilization and depolarization‐dependent modes of cell death. The cell death study also highlighted the significance the substituent functional groups and their location within the structure plays on the extent of cell death and the mechanism they elucidate. The compounds provide a scaffold for a potential novel GBM therapy, capable of selective cytotoxic effects on cancer cells.

## 2 Results and Discussion

Despite ongoing advances in cancer therapy, GBM remains a highly aggressive cancer with a poor prognosis. Survival outcomes have seen minimal improvement, with the median survival still approximately 15 months.^[^ ^36^ ^]^ A common drawback of the current treatment strategy is its lack of selectivity towards cancer cells, often leading to adverse effects and a decrease in the quality of life of the patient. Furthermore, the reoccurrence rates remain high due to the development of cancer cell resistance to radiotherapy and TMZ.^[^ ^36^ ^,^ ^37^ ^]^ Pharmacological approaches which are selectively cytotoxic for GBM cancer cells are an interesting avenue for efficient GBM treatment with decreased occurrence of side effects, improving the quality of life and survival rates of the patients. Others have shown the potential of pyrazolopyrimidinones as anticancer agents in several cell lines, but little is known about their selective capacity.^[^ ^11^ ^,^ ^19^ ^,^ ^38^, ^39^ ^–^ ^40^ ^]^

In this study, we aimed to evaluate pyrazolo[1,5‐*α*]pyrimidinones derivatives for efficacy against GBM and their selectivity potential. Four groups of novel pyrazolo[1,5‐*α*]pyrimidinones derivatives were synthesized, each with varied R^1^ and R^2^ substituents: groups 1, 2, 3, and 4 (Figure 1). The groups were screened against the U‐251 MG GBM cell line and the noncancerous embryonic kidney cell line, HEK293, to assess the potential of the newly synthesized family as a selective anti‐GBM therapy. The cytotoxicity of compounds in each group was evaluated via the MTT assay to identify HIT and subsequently lead compounds with the highest cytotoxicity against the U‐251 MG cells and lower cytotoxicity against the HEK293 cells. Compounds with IC_50_ values lower than 50 μM and a narrow IC_50_ 95% confidence intervals were deemed to elicit a sufficient biological response.^[^ ^41^ ^]^

### 2.1 Chemistry

**Scheme 1:**
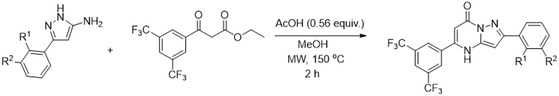
Synthesis of lead pyrazolo[1,5‐*α*]pyrimidinones, **21** and **22**. For **21**, R^1^ = H, R^2^ = Br, and for **22**, R^1^ = Br and R^2^ = H.

Experimental procedures leading to pyrazolo[1,5‐*α*]pyrimidinones are reported in the Supporting Information. The two lead compounds, **21** and **22** **,** were prepared by reacting ethyl 3‐(3,5‐bis(trifluoromethyl)phenyl)‐3‐oxopropanoate with their corresponding 5‐aminopyrazole and AcOH in MeOH. The reaction mixture was subjected to microwave irradiation (100 W, 150 °C) for 2 h (**Scheme** **1**). Pyrazolo[1‐5‐*α*]pyrimidinone **21** was purified via hot filtration with MeOH to yield the product in a 33% yield. Pyrazolo[1‐5‐*α*]pyrimidinone **22** was purified via column chromatography using silica gel with petroleum ether/ethyl acetate as eluent (70:30 to 100:0), with a 68% yield. The remaining compounds were synthesized using similar procedures and all compounds were characterized by ^1^H NMR, ^13^C NMR, 2D NMR, high‐resolution mass spectrometry (HR‐MS), and high‐performance liquid chromatography (HPLC); see Supporting information.

**Scheme 2:**
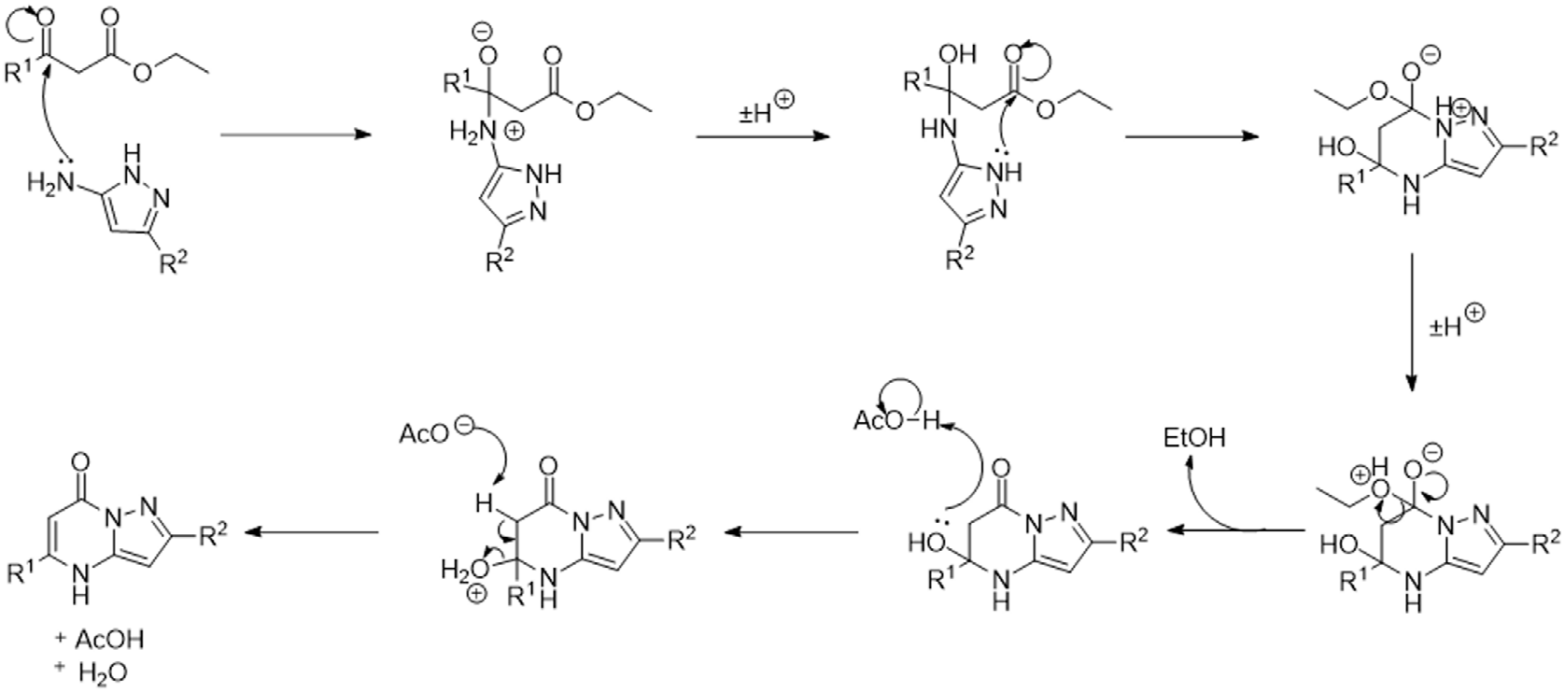
Proposed mechanism for the synthesis of the pyrazolo[1,5‐ *α*]pyrimidinones,.

A proposed mechanism is described in **Scheme** **2**. This begins with nucleophilic attack of the pyrazole primary amino group on the keto carbonyl carbon. A subsequent 6‐exo trig cyclization and sequential loss of EtOH and H_2_O gives the pyrazolo[1,5‐*α*]pyrimidinone.

#### 2.1.1 Group 1 Compounds

Group 1 pyrazolo[1,5‐*α*]pyrimidinones derivatives **1–4** were synthesized to include a variety of substituents at the R^1^ substituent, while the R^2^ substituent was retained as an isopropyl group. Pyrazolo[1,5‐*α*]pyrimidinone derivatives containing an isopropyl group substituent at R^2^ have previously shown cytotoxic effects on GBM cells.^[^ ^19^ ^]^ Compound **1** showed no biological activity against the U‐251 MG and HEK293 cell lines, whereby the IC_50_ values exceeding 50 µM were determined experimentally and did not bear a significant biological effect as per set threshold of <50 µM, in addition to large IC_50_ value ranges in both cell lines (**Table** **1**, Figure S47, Supporting Information). Interestingly, other compounds in the group, compounds **2**, **3**, and **4** exhibited lower IC_50_ values (<20 µM) against U‐251 MG cells, indicating a relevant biological effect. However, they lacked the desired selectivity for cancer cells with the compounds exhibiting a more cytotoxic effect towards noncancerous HEK293 cells, making them unsuitable as HIT compounds in this study (Table 1, Figure S47, Supporting Information). Hence, group 1 compounds did not show potential as a selective therapy for GBM and were not further studied.

**Table 1:** Summary of the cytotoxicity results and statistical analysis of groups **1**, **2**, **3**, and **4** pyrazolo[1,5‐α]pyrimidinone treatment against U‐251 MG and HEK293 cells.

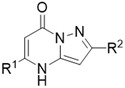											
Compound	Ra) group	R^2^ group	U‐251 MGa)		Hillslope	HEK293		Hillslope	Statistically distinct curves ‐Yes/No *P value*	Difference in IC_50_ ranges‐Yes/No	Selectivity for cancer cells with IC_50_ < 50 [µM]‐Yes/No
			IC_50_ [μM]	95% CI [μM]		IC_50_ [μM]	95% CI [μM]				
**1**	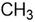	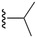	118.6	66.34 to 822.5	−0.5490	449.6	137.3 to 130,351	−0.4501	Yes 0.0468	No	No
**2**	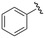	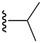	20.60	16.46 to 25.51	−1.273	9.802	6.539 to 14.40	−1.375	Yes *0.0024*	Yes	No
**3**	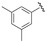	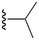	9.289	6.220 to 13.28	−0.5025	6.517	4.508 to 8.762	−1.114	Yes* < 0.0001*	No	No
**4**	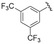	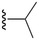	14.02	11.19 to 17.32	−1.384	7.383	6.377 to 8.568	−2.838	Yes* < 0.0001*	Yes	No
**5**	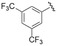	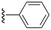	6.172	3.721 to 10.17	−0.2984	15.35	12.19 to 17.92	−1.491	Yes* < 0.0001*	Yes	Yes
**6**	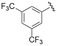	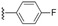	52.18	45.79 to 60.12	−5.948	68.22	62.84 to 73.30	−7.075	Yes *0.0068*	Yes	No
**7**	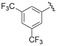	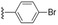	29.82	25.81 to 33.26	−3.243	60.88	X	−10.05	Yes* < 0.0001*	X	Yes
**8**	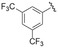	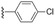	17.30	15.25 to 19.57	−1.561	52.37	45.33 to 58.51	−3.976	Yes* < 0.0001*	Yes	Yes
**9**	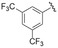	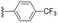	27.46	24.35 to 31.22	−3.665	54.43	49.95 to 62.95	−10.95	Yes* < 0.0001*	Yes	Yes
**10**	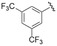	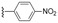	29.62	25.37 to 34.75	−3.232	73.78	65.47 to 83.01	−4.962	Yes* < 0.0001*	Yes	Yes
**11**	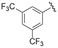	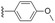	23.48	18.75 to 28.98	−0.9642	29.48	25.52 to 34.83	−4.056	Yes* < 0.0001*	No	No
**12**	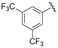	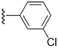	21.65	16.11 to 28.34	−0.8934	29.48	25.52 to 34.83	−4.056	Yes* < 0.0001*	No	Yes
**13**	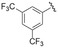	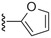	X	X	X	X	X	X	X	X	X
**14**	CH_3_	Ph	81.49	64.61 to 117.7	−1.263	X	X	X	X	X	X
**15**	Ph	Ph	19.92	17.25 to 22.92	−1.614	15.92	X	−5.573	Yes < 0.0001	X	No
**16**	CH_3_	CH_3_	X	X	X	X	X	X	X	X	X
**17**	Ph	t‐Bu	76.58	69.35 to 83.88	−5.735	X	X	X	X	X	X
**18**	i‐Pr	i‐Pr	170.0	X to 5429	−2.055	117.7	69.24 to 374.2	−0.5581	Yes* < 0.0001*	X	No
**19**	Et	i‐Pr	154.0	100.0 to 2410	−1.995	X	X	X	X	X	X

a) The null hypothesis was such that to be statistically significant the IC_50_ values for both cell lines would have two separate curves, the p value generated via the F‐test must be <0.05 and both IC_50_ ranges must not overlap. Compounds are to be considered HIT compounds if the IC_50_ value for HEK293 is higher than IC_50_ for U‐251 MG cells, and the biological response for U‐251 MG is below the 50 µM IC_50_ value criteria. X is denoted where an experimental value was not possible to determine with the concentrations used (100–0 µM).

#### 2.1.2 Group 2 Compounds

Group 2 pyrazolo[1,5‐*α*]pyrimidinones derivatives **5–12** were synthesized to retain the 1,3‐bis(trifluoromethyl)phenyl as the R^1^ substituent, while the R^2^ substituents was varied aryl group or heterocycle. The anti‐GBM role of 1,3‐bis(trifluoromethyl)phenyl at R^1^ position in the pyrazolopyrimidinone derivatives has been previously highlighted and further evaluated in this study.^[^ ^19^ ^]^ All group 2 compounds elicited a significant biological response towards U‐251 MG with IC_50_ values below the set 50 µM threshold, except for **6** at 52.18 µM and **13** which showed no cytotoxicity towards the GBM and embryonic kidney cell lines at the concentrations used (Table 1, Figure S47, Supporting Information). Hence, **13** did not show a potential as a selective therapy for GBM, and its *R* ^2^ functional group did not bear significance for this study.

Compounds **5**, **7**, **8**, **9**, and **10** elicited a biological response against the GBM cell line but did not elicit any biological response in noncancerous HEK293 cells, highlighting the selective capacity of these compounds (Table 1, Figure S47, Supporting Information). For instance, **10** showed the most selectivity with a difference of 44.16 µM between the IC_50_ values. However, to consider the compounds as a HIT they must show 2 statistically distinct IC_50_ curves without overlapping IC_50_ ranges and a F–test curve fitting p‐value of < 0.05 (Table 1). The HIT compounds must also show statistically differential response to pyrazolopyrimidinone treatment when analyzed via two‐way ANOVA with Tukey's post‐test (Table S1, Supporting Information). The ranges overlapped for two compounds, **11** and **12**, and a range for **7** in HEK293 cells could not be experimentally determined (Table 1, Figure S47, Supporting Information).

Furthermore, **5**, **11**, and **12** all exhibited significant cytotoxicity against the noncancerous cell lines at IC_50_ values below 30 µM, indicating that the lack of functional groups at *R* ^2^ or the addition of O‐CH_3_ at the *para* position and Cl at the *meta* position enhanced the cytotoxicity of the compounds against noncancerous cells. While cytotoxicity against cancer cells remained relatively comparable to others in the group, **5** exhibited the highest anticancer effect at 6.17 µM. Interestingly, the cytotoxicity and selectivity of **8** with Cl at *para* position was higher than that of **12** with Cl at *meta* position (Table 1). Cl can significantly increase the potency and lipophilicity of drugs and their affinity and interaction with target proteins, which can be impacted by the location of the Cl in the drug molecule.^[^ ^42^ ^,^ ^43^ ^]^ From the initial screening, compounds of interest, which passed all statistical and set criteria, were compounds **5, 7, 8**, **9**, and **10** (Table 1, Table S1, Supporting Information). Hence, group 2 compounds with the aryl substituents did show potential as a selective therapy for GBM and was therefore used to as a scaffold for further HIT optimization.

#### 2.1.3 Group 3 Compounds

Group 3 pyrazolo[1,5‐*α*]pyrimidinones derivatives, **14** and **15**, were synthesized to include varied R^1^ groups with the aryl phenyl at R^2^. While **14** did not elicit a biological effect in either cell lines, **15** elicited a significant biological effect in both cell lines (Table 1, Table S1, Figure S47, Supporting Information). **15** has previously shown cytotoxicity against U‐251 MG cells by He and colleagues (2021) at 77.68 µM after 2 days of treatment, compared to 19.92 µM after 5 days, suggesting cytotoxicity increases with exposure time. Regardless of the significant biological effect of **15**, no selectivity for the cancer cells was observed. In fact, **15** proved to be more cytotoxic towards noncancerous cells at 15.92 µM (Table 1). Due to this lack of selectivity, group 3 compounds were not further explored.

#### 2.1.4 Group 4 Compounds

Group 4 pyrazolo[1,5‐*α*]pyrimidinone derivatives **16–19** were synthesized to include varied substituents at both *R* ^1^ and *R* ^2^. Group 4 has previously shown potential as He and colleagues (2021) identified two lead prodrug candidates based on the structure of group 4. However, alone, the candidates had marginal cytotoxicity towards U‐251 MG GBM cells at IC_50_ values exceeding 200 µM. Therefore, further derivatives were synthesized to investigate if cytotoxicity could be modulated and to further evaluate the potential of group 4 as anti‐GBM agents. The synthesized derivatives, however, did not demonstrate enhanced cytotoxicity towards GBM cells at IC_50_ values considerably larger than the set threshold of 50 µM and, hence, were not studied further (Table 1, Figure S47, Supporting Information).

### 2.2 HIT Compounds

The evaluation of the pyrazolo[1,5‐*α*]pyrimidinone derivatives was carried out on two cell lines, the U‐251 MG cell line and the HEK293 cell line. Experimental IC_50_ values and the statistical analysis of generated curves are summarized and compared in Table 1. HIT compounds were identified based on three criteria: strong cytotoxicity against U‐251 MG cells, minimal or no cytotoxicity toward HEK293 cells, and statistically distinct (*p* < 0.05), nonoverlapping dose–response curves with significantly different IC_5__0_ values and ranges (Table 1). Statistical validation was performed using two‐way ANOVA with Tukey's post‐test (Table S1, Supporting Information).

Out of 19 candidates, three compounds have met all three HIT criteria and passed the ANOVA statistical test and hence have been identified as the HIT compounds: **8**, **9**, and **10.** These compounds showed potent activity against U‐251 MG cells (IC_50_ < 30 µM) and low toxicity in HEK293 cells (IC_50_ > 50 µM), with two statistically distinct curves and summary p‐values of <0.0001 (****), confirming their selective anticancer potential. Compound **7** also showed potential regardless of its lack of IC_50_ ranges for the HEK293 cell line, as it met all other criteria and it contains the bromine functional group which is known to highly modulate the bioactivity of compounds.^[^ ^39^ ^,^ ^44^ ^]^ Compound **7** was chosen for HIT optimization to further study the effects bromine may play in the bioactivity of our compounds. Since the chlorine substituent positioning was tested with compounds **8** and **12,** compound **8** was not chosen for further evaluation and HIT optimization. Based on these findings and to give a range of substituent types explored, compounds **7**, **9**, and **10** were selected for further study and to investigate their potential as a selective GBM therapy.

Compound **5**, bearing only an unsubstituted aryl ring at *R* ^2^, has also shown selectivity for cancer cells, though it still elicited a response in HEK293 cells at concentrations below 50 µM. Notably, compound **5** exhibited the strongest anti‐GBM activity of all candidates, with an IC_50_ of 6.17 µM. Additionally, **5** proved to have two statistically distinct curves with nonoverlapping IC_50_ ranges and had a statistically significant differential effect in the two cell lines at summary p‐value of < 0.001 (****) (Table S1, Supporting Information). Hence, **5** was chosen for further HIT optimization due to its notable anticancer activity and structural value in understanding the relationship between structural change and biological effects (Figure S48, Supporting Information).

Compounds **7**, **9**, and **10** share the basic structure of **5**, featuring a 1,3‐bis(trifluoromethyl)phenyl substituent at R^1^, while the R^2^ substituents included an aryl ring bearing an electron withdrawing group, i.e., NO_2_, Br, or CF_3_ (**Table** **2**, Figure S47, Supporting Information). Br and CF_3_ are common lipophilic groups that can increase the lipophilicity of compounds. While all R^2^ substituents were statistically significant at p‐value < 0.0001, the NO_2_ group showed the greatest IC_50_ difference between U‐251 MG and HEK293 cells, followed by the Br and CF_3_ substituents (Table 1).

**Table 2:** Analysis of the predicted physicochemical properties of compounds **5**, **7**, **9**, and **10**.^[^ ^44^ ^]^

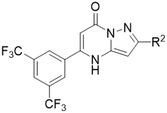						
Compound	*R* ^2^ group	Molecular weight [g mol^−1^]	LogP	# H bond donor	# H bond acceptor	Polar surface area [Å^2^]
**5**	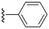	423.32	5.31	1	8	50.16
**7**	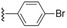	502.21	5.95	1	8	50.16
**9**	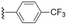	491.31	6.32	1	11	50.16
**10**	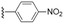	468.31	4.60	1	10	95.98

Since the functional groups seemed to significantly augment the activity of the compounds, the physicochemical properties of the compounds chosen for HIT optimization, such as lipophilicity, molecular weight, polar surface area, and number of hydrogen bond donors and acceptors, were predicted and evaluated. These are important factors in terms of bioactivity and crossing the BBB.^[^ ^44^ ^]^ These properties were assessed using Lipinski's Rule of Five as a guideline to estimate oral bioavailability (Table 2). Most HIT compounds obey the Lipinski's rule for bioavailability with violations regarding the LogP for all compounds, as well as 14 hydrogen bond acceptors for **9**, and a higher molecular weight of 502.21 g/mol for **7**. As the violations are close to the acceptable ranges, it is possible the compounds will still exhibit good oral bioavailability, as many of currently approved drugs also violate Lipinski's rules (Table 2).^[^ ^45^ ^]^

Lipophilic compounds with a low molecular weight (between 400 and 600 g/mol), fewer hydrogen bond donors and a lower polar surface area (<90 Å^2^) have a higher likelihood of crossing the BBB.^[^ ^32^, ^33^ ^–^ ^34^ ^]^ The compounds chosen for HIT optimization are all highly lipophilic with predicted LogP values of 5.28–6.20, where higher values represent higher lipophilicity.^[^ ^34^ ^]^ Lipophilic compounds are favored for BBB permeability as they can readily pass through the lipid bilayer of the cell membrane. However, too high of a lipophilicity can hinder the effect of the drug as it could fail to exit the lipid bilayer and can become trapped, diminishing the effect it may have on target cells. Additionally, the concentration of the drug might be decreased by uptake by the peripheral tissues, resulting in the use of higher concentration and enhanced probability of side effects. The high lipophilicity of the compounds may therefore become an obstacle in the efficacy of the compounds to cross the BBB, and furthermore alternative administration routes should be explored.^[^ ^33^ ^,^ ^46^ ^]^ Regarding the size of the compounds, all adhere to the acceptable 400–600 g mol^‐^^1^ range and have only 1 hydrogen bond donor. The polar surface area remains low for all compounds at 50.17 Å^2^ bar **10** at 95.99 Å^2^.

#### 2.2.1 HIT Compound's Analogs

Based on the identified HIT compounds, a further four analogs were synthesized, compounds **20**, **21**, **22**, and **23**. The new analogs had the R^2^ *para* substituent changed to an *ortho* or a *meta* position (**Table** **3**). Other positions were also considered but could not be accessed synthetically. Their anticancer properties were evaluated along with their selectivity for cancerous U‐251 MG cell line over a noncancerous HEK293 cell line (Figure S49, Supporting Information).

**Table 3:** Summary of cytotoxicity results and statistical analysis of HIT analog treatment against U‐251 MG and HEK293 cells.

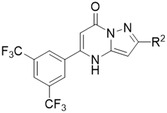											
		U‐251MGa)			HEK293						
Compound	Ra) group	IC_50_ [μM]	95% CI [μM]	Hillslope	IC_50_ [μM]	95% CI [μM]	Hillslope	Statistically different curves‐Yes/No *P value*	Difference in IC_50_ ranges ‐Yes/No	Selectivity for cancer cells with IC_50_ < 50µM ‐Yes/No	ANOVA summary
**20**	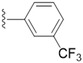	30.97	27.15 to 35.55	−4.045	X	X	X	X	X	X	**
**21**	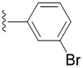	0.71	0.1915 to 1.878	−0.3128	19.04	16.58 to 21.79	−1.907	Yes < 0.0001	Yes	Yes	****
**22**	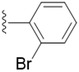	2.79	1.025 to 5.911	−0.2934	66.91	55.68 to 76.84	−3.518	Yes* < 0.0001*	Yes	Yes	****
**23**	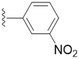	23.19	16.88 to 30.68	−1.177	60.32	54.10 to 67.29	−6.753	Yes < 0.0001	Yes	Yes	****

a) The null hypothesis was such that to be statistically significant the IC_50_ values for both cell lines would have two separate curves, the p value generated via the F‐test must be <0.05, both IC_50_ ranges must not overlap, and the ANOVA analysis must show significance at p‐value <0.05. Compounds are to be considered HIT compounds if the IC_50_ value for HEK293 is higher than IC_50_ for U‐251 MG cells, and the biological response for U‐251 MG is below the 50 µM IC_50_ value criteria. *X is denoted where an experimental value was not possible.*

Upon carrying out two‐way ANOVA with Tukey's post‐test to compare the effects of compounds in both cell lines, statistical significance was determined and **21**, **22**, and **23** were identified as new HIT compounds at p‐value < 0.0001 (Table S1, Supporting Information). The lead compound was identified as the *ortho* positioned Br derivative, **22**, as it met all set criteria and it exhibited the largest difference in IC_50_'s between both cell lines with a strong significant cytotoxicity against the U‐251 MG cell line at 2.79 µM and relevantly marginal cytotoxicity against the noncancerous HEK293 cells at 66.91 µM, indicating a strong selectivity for cancer cells. Bromination of compounds often corresponds to altered drug potency as the bromine can increase the lipophilicity of the compound and alter its stability, solubility, and its interaction with the biological target depending on its location within the compound, as evident by **7, 21**, and **22,** where bromination had significant effects on the cytotoxicity and selectivity of the compounds (**Table** **4**).^[^ ^47^ ^]^

**Table 4:** A comparison of the changes in the cytotoxicity of **7**, **21**, and **22** when the bromo substituent is moved to different positions in the phenyl ring.

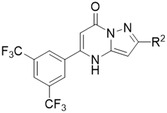			
Compound	*R* ^2^ group	U‐251 MG IC_50_ value [μM]	HEK293 IC_50_ value [μM]
**7**	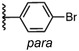	29.49	60.88
**21**	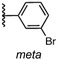	0.71	19.04
**22**	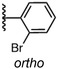	2.79	66.91

Compound **21** also passed all tests of significance and had a high cytotoxic effect against the U‐251 MG cells at 0.71 µM. However, it exhibited a biological response in HEK293 cells at 19.04 µM, indicating the *ortho* position of the Br somehow aids the selectivity of the compounds for cancer cells over noncancerous embryonic kidney cells. Additionally, changing the position of the Br around the phenyl ring alters the anti‐GBM properties of the compounds; with the highest cytotoxicity with the *meta* position, followed by *ortho* (which showed superior selectivity), and *para* which showed lesser cytotoxicity but good selectivity (Table 4). The NO_2_ derivatives, **10** and **23**, showed good selectivity but lesser GBM cytotoxicity, compared to **21** and **22,** at IC_50_ values of 23.19 and 29.62 µM for U‐251 MG, respectively (Table 1, 3). The alteration of the position of the CF_3_ substituent from *para* to *meta* position did not notably change the cytotoxicity of the compounds, with overlapping IC_50_ ranges. Furthermore, the effect on the HEK293 cells could not be reliably calculated for **20**; hence, no conclusive statements can be made on the impact of the *meta* position on selectivity (Table 1 and 3).

### 2.3 Evaluation of the Mode of Cell Death

Flow cytometry was utilized to determine whether the HIT compounds elicit the cell death observed by the MTT assay via cell membrane permeabilization dependent and mitochondrial membrane potential dependent mechanisms such as necrosis and apoptosis. Previous studies have highlighted the role of pyrazolo[1,5‐*α*]pyrimidinone derivatives as kinase inhibitors, which can promote cell death through mechanisms such as apoptosis, autophagy, and necrosis.^[^ ^11^ ^,^ ^19^ ^,^ ^38^ ^,^ ^40^ ^,^ ^48^, ^49^ ^–^ ^50^ ^]^ For instance, Ali et al. (2019) demonstrated pyrazolo[1,5‐a]pyrimidine derivatives can inhibit the CDK2/cyclin A2 enzyme, a key player in the cell cycle, whose upregulation is linked to various cancers.^[^ ^50^ ^]^ The tested derivatives showed anti‐cancer properties in four cancer cell lines, and the HIT compounds induced cell cycle arrest and inhibited proliferation of HCT‐116 colon cancer cells and proved to cause apoptotic DNA fragmentation.^[^ ^50^ ^]^

#### 2.3.1 Evaluation of Cell Membrane Permeabilization as the Mode of Cell Death

**Figure 2:**
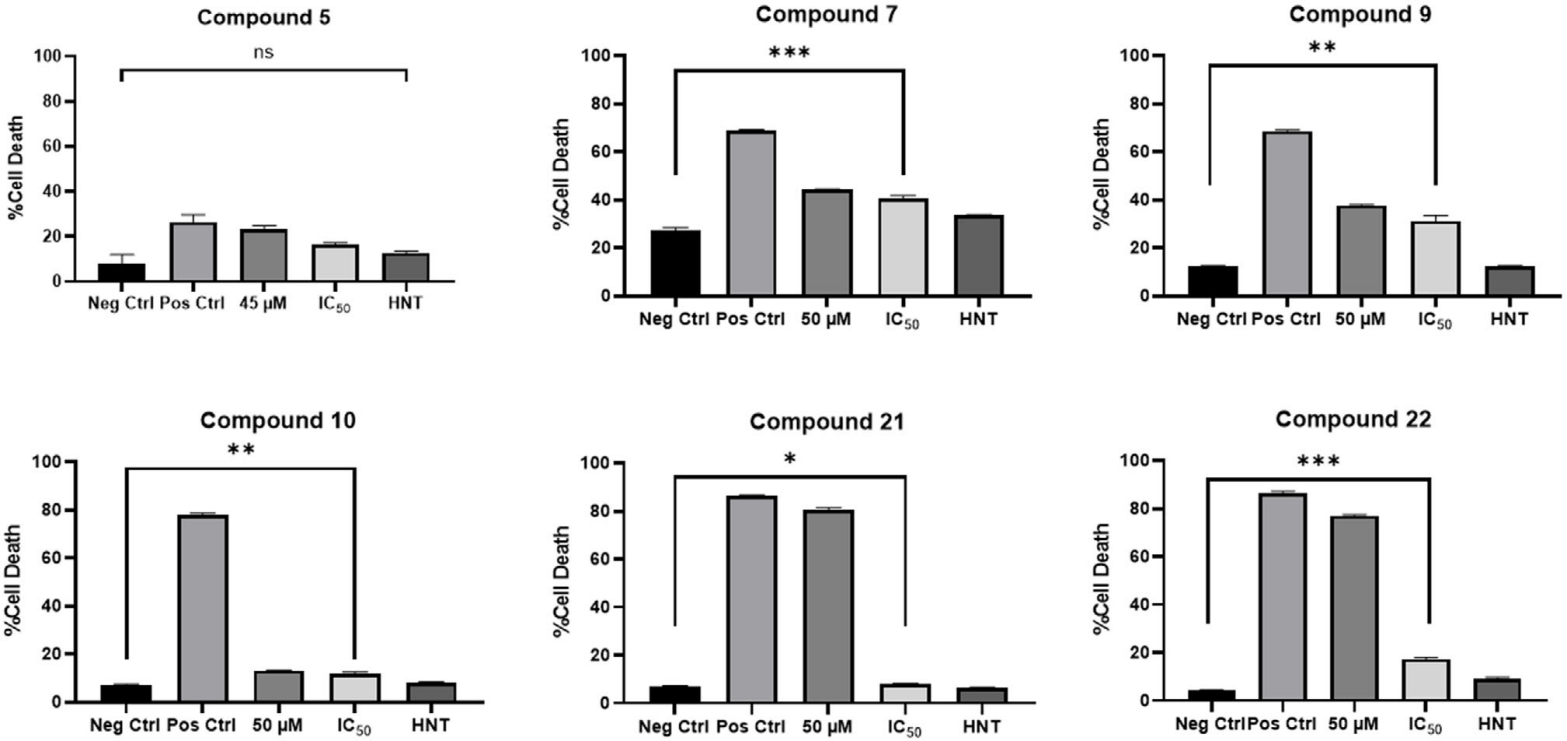
Flow cytometry analysis of HIT compound treatments on U‐251 MG GBM cells using PI dye. Cells were treated with HIT compound for 5 days, followed by analysis via PI flow cytometry. The statistical significance between the negative control and the IC_50_ compound concentration was determined via one‐way ANOVA with Dunnett's T3 multiple comparison post‐test. Statistical significance is represented as ≥0.05 (ns), 0.01 to 0.05 (*), 0.001 to 0.01 (**), 0.001 to 0.01(***), and <0.0001(****).

To evaluate the compounds for cell membrane permeabilization dependent cell death, U‐251 MG cells were treated with a positive control and with varied concentrations of the HIT compounds, including the highest toxic concentrations, the IC_50_ value and the highest nontoxic drug concentration (Table S2, Supporting Information), and analyzed with propidium iodide (PI). PI is a cell membrane impermeable DNA staining dye. Alone, PI can enter late apoptotic and necrotic cells whose membranes have been compromised. The compound **5** IC_50_ treatment did not induce cell death by cell wall permeabilization as the number of necrotic cells observed was not statistically significant when compared to the negative control. There was also no statistical difference between the negative control and the highest nontoxic concentration of **5** (**Figure** **2**), hence indicating that **5** did not cause cell death based on cell permeabilization and that other mode(s) of action are occurring.

Data suggests that the remaining HIT compounds (**7**, **9**, **10**, **21**, and **22**) all exhibit significant cell membrane permeabilization‐dependent cell death such as necrosis or apoptosis (Figure 2). Compounds **7** and **22** are most likely to induce cell membrane permeabilization‐dependent cell death due to the high significance observed between the negative control and their corresponding IC_50_ concentrations (p‐value < 0.001***); while **21** only showed a p‐value of < 0.05 which indicates weak evidence of permeabilization dependent cell death (Figure 2, Table S3, Supporting Information). Results indicate that the addition of the Br functional group at *ortho* and *para* positions promotes cell death via cell permeabilization. As addition of bromine can alter the stability and the interaction of the drugs with the biological target, the bromination of the pyrazolopyrimidinones could potentially lead to enhanced apoptosis or necrosis.^[^ ^47^ ^]^ PI staining was a useful tool to show that the HIT compounds induce cell death; however, it was not necessarily necrosis or apoptosis. Compound **5,** which lacked functional groups on the aryl ring, showed no significant evidence of cell death induced via cell permeabilization. Hence, data suggests that by the addition of the functional groups on the aryl ring, the likelihood of cell membrane permeabilization‐dependent cell death increases. Hence, subsequently, JC‐1 dye was used to further investigate the possible mode of cell death of **5** and other HIT compounds.

#### 2.3.2 Evaluation of Apoptosis as Mode of Cell Death

**Figure 3:**
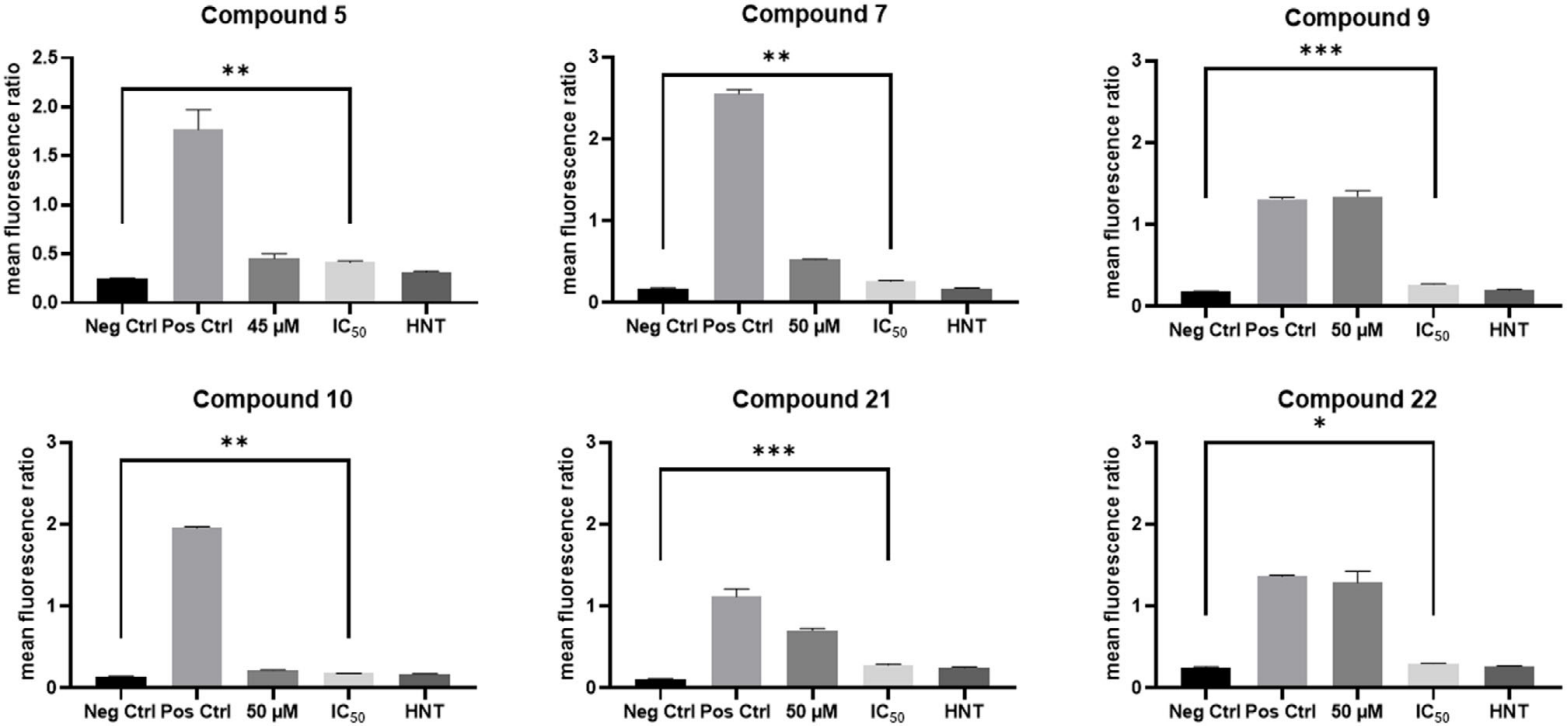
Flow cytometry analysis of HIT compound treatments on U‐251 MG GBM cells using JC‐1 dye. Cells were treated with HIT compound for 5 days, followed by analysis via PI flow cytometry. The statistical significance between the negative control and the IC_50_ compound concentration was determined via one‐way ANOVA with Dunnett's T3 multiple comparison post‐test. Statistical significance is represented as: ≥0.05 (ns), 0.01 to 0.05 (*), 0.001 to 0.01 (**), 0.001 to 0.01(***), and < 0.0001(****).

To evaluate the compounds for apoptosis via changes in the mitochondrial membrane potential, U‐251 MG cells were treated with a positive control and with compounds as per Table S2, Supporting Information, and analyzed with the JC‐1 dye. JC‐1 distinguishes healthy and apoptotic cells based on its ability to form red fluorescent aggregates in intact mitochondria found in healthy cells. A one‐way ANOVA with Dunnett's T3 post‐test was carried out whereby each treatment was compared to the negative control to determine statistical significance. There is statistical significance between the negative control and the IC_50_ of the HIT compound **5** (**Figure** **3**) which is eliciting enough apoptosis to be deemed statistically significant with a *p* value of < 0.01**.

Analysis of the remaining compounds indicated that the most statistically significant compounds were **9** and **21** and to a lesser extent **7**, **10**, and **22** inferring a change in the mitochondrial membrane potential which is indicative of apoptosis (Figure 3, Table S4, Supporting Information).^[^ ^40^ ^]^ Interestingly, the most significant compounds did not share a common functional group; instead, their substituents were *para* positioned CF_3_ and *meta* positioned Br indicating that the functional group and their position within the compound structure plays a significant role on the mode of cell death and cytotoxicity. Since all HIT compounds showed statistical significance at *p* > 0.05, apoptosis could be a potential mode of cell death as observed by others with varied pyrazolo[1,5‐*α*]pyrimidinone derivatives which were found to inhibit key cellular kinases and which can act in a pro‐apoptotic manner.^[^ ^49^ ^,^ ^51^, ^52^ ^–^ ^53^ ^]^

The results obtained via flow cytometry have shown that the HIT compounds are eliciting cell death either through cell permeabilization dependent manner or apoptotic manner in the U‐251 MG cell line, confirming the MTT cell viability assay results. However, the exact mode of cell death is still unknown and warrants further investigation as different compounds showed different levels of significance in response to PI and JC‐1 analysis as clearly evident by compound **5** (*p* > 0.05^ns^ with PI and *p* < 0.01** with JC‐1; Figure 3, Table S4, Supporting Information). Additionally, the exact impact of the functional groups on the aryl ring on the mode of cell death remains unclear, as well as the effect of the therapy on noncancerous cells. Furthermore, the mode of cell death and the efficacy of the therapy are to be further investigated in a model which more closely mimics the in vivo environment, as the limitation of 2D models has been heavily reported.^[^ ^54^, ^55^, ^56^ ^–^ ^57^ ^]^ Notably, the identified HIT compounds did show capacity at selective induction of cancer cell death, highlighting the potential of the pyrazolopyrimidinones therapy as a developing selective therapy for GBM. The HIT compounds can also be used as a scaffold in further studies to develop a novel selective anticancer therapy.

## 3 Conclusion

GBM is the most common and aggressive type of brain tumor, which is difficult to treat due to a high incidence of resistance to conventional chemotherapeutic options. Here, an initial 19 pyrazolo[1,5‐*α*]pyrimidinone compounds were successfully synthesized and assessed for cytotoxicity against GBM and embryonic kidney cell lines to determine efficacy as a selective anti‐GBM treatment. From an initial SAR study, four HIT compounds were identified which exhibited significant GBM cytotoxicity while demonstrating a marginal effect on the embryonic kidney cells, indicating selective capacity. The compounds contained lipophilic and electron withdrawing *R* ^2^ substituents except for **10** which contained a strong electron withdrawing group but was not lipophilic. HIT optimization identified three compounds exhibiting strong anti‐GBM activity with selectivity for the GBM cell line over the noncancerous cell line. Evaluation of the mode of action via flow cytometry identified that the HIT compounds can induce cell death by either membrane permeabilization or mitochondria membrane depolarization‐dependent modes of cell death such as necrosis or apoptosis. The lead compound, **22**, has been identified which has shown significant potential to be used as a novel chemotherapeutic option for the treatment of GBM, capable of selectivity towards cancer cells. The mode of cell death studies suggests the compounds induce cell death by cell membrane permeabilization and mitochondria membrane depolarization‐dependent modes of cell death such as apoptosis and necrosis. Pyrazolopyrimidinones demonstrate the potential for selectively targeting GBM cells, making them promising, minimally toxic, therapeutic candidates warranting further study.

## 4 Experimental Section

4.1

4.1.1

##### General Procedure of the Synthesis of Pyrazolopyrimidinones via Microwave

A microwave vial (2 mL) was charged with the required 5‐aminopyrazole (0.45 mmol, 1 equiv), *β*‐ketoester (0.675 mmol, 1.5 equiv), and AcOH (14.3 μL, 0.25 mmol, 0.56 equiv) in MeOH (1 mL) and was subjected to MW irradiation (100 W, 150 °C) for 2 h. The resulting mixture was concentrated under reduced pressure, and the residue was purified via column chromatography or trituration in MeOH/ water to give the title compound. Some compounds, **17**, **23**, were purified further via hot recrystallization in EtOH.

##### General Procedure of the Synthesis of Pyrazolopyrimidinones via Reflux

A round‐bottom flask (10 mL) was charged with the required 5‐aminopyrazole (0.45 mmol, 1 equiv), *β*‐ketoester (0.675 mmol, 1.5 equiv), and AcOH (14.3 μL, 0.25 mmol, 0.56 equiv) in MeOH (3 mL). The reaction mixture was stirred under reflux for 20 h. After completion, the solvent was removed under reduced pressure, and the residue was purified via column chromatography or trituration to give the title compound. Some compounds were purified further via recrystallization in EtOH.

##### 5‐(3,5‐Bis(trifluoromethyl)phenyl)‐2‐(3‐bromophenyl)pyrazolo[1,5‐α]pyrimidin‐7(4¬H)‐one (21)

3‐(2‐Bromophenyl)‐1H‐pyrazol‐5‐amine (0.220 g, 0.9 mmol, 1 equiv), ethyl 3‐(3,5‐bis(trifluoromethyl)phenyl)‐3‐oxopropanoate, (0.650 g, 1.98 mmol, 2.2 equiv), AcOH (42.9 μL, 0.75 mmol, 0.56 equiv), and MeOH (3 mL) were subjected to MW irradiation (100 W, 150 °C) for 2 h. The MeOH was removed under reduced pressure to afford a residue, which was purified by column chromatography using a gradient elution from 70:30 petroleum ether/EtOAc to 100% EtOAc.d. Yield 0.306 g, 68%; *R*_f_ 0.65 (EtOAc); ^1^H NMR (500 MHz, DMSO) *δ* 8.67 (s, 2H, H3), 8.12 (s, 1H, H1), 7.81 (d, *J* = 5.6 Hz, 1H, H14), 7.73 (d, *J* = 8.0 Hz, 1H, H11), 7.44 (t, *J* = 7.1 Hz, 1H, H13), 7.31 (t, *J* = 7.5 Hz, 1H, H12), 6.60 (s, 1H, H8), 6.30 (s, 1H, H6); ^13^C NMR (125 MHz, DMSO) *δ* 158.4 (*C*O), 152.7 (C5), 151.67 (C9) 148.5 (C7), 142.0 (C4), 135.4 (C10), 133.2 (C11), 131.8 (C14), 130.5 (q, *J* = 25 Hz, C2), 129.6 (C12), 127.5 (C13), 126.9 (C3), 123.4 (q, *J* = 275 Hz (*C*F_3_), 122.4 (C15), 121.9 (C1), 94.4 (C8), 89.2 (C6); ^19^F NMR (471 MHz, DMSO) *δ* −61.21 (C*F*_3_); IR (ATR) 2922, 1605, 1540, 1276, 1127, 680 cm^−1^; HR‐MS calcd for C_20_H_10_BrF_6_N_3_ONa m/z: [M + Na]^+^, 523.9911; found [M + Na]^+^ = 523.9803 [Diff (ppm) = −0.29].

##### 5‐(3,5‐Bis(trifluoromethyl)phenyl)‐2‐(3‐bromophenyl)pyrazolo[1,5‐*α*]pyrimidin‐7(4­*H*)‐one (22)

3‐(3‐Bromophenyl)‐1H‐pyrazol‐5‐amine (0.22g, 0.9 mmol, 1 equiv), ethyl 3‐(3,5‐bis(trifluoromethyl)phenyl)‐3‐oxopropanoate (0.650 g, 1.98 mmol, 2.2 equiv), AcOH (42.9 μL, 0.75 mmol, 0.56 equiv), and MeOH (5 mL) were subjected to MW irradiation (100 W, 150 °C) for 2 hr. The resulting mixture was purified using hot filtration with MeOH (3 ml). Yield 0.15 g, 33%; *R*_f_: 0.61 (EtOAc); ^1^H NMR (500 MHz, DMSO) *δ* 12.93 (s, 1H, N*H*), 8.54 (s, 2H, H3), 8.36 (s, 1H, H1), 8.20 (s, 1H, H15), 8.04 (d, *J* = 7.8 Hz, 1H, H11), 7.63 (d, *J* = 8.0 Hz, 1H, H13), 7.46 (t, *J* = 7.9 Hz, 1H, H12), 6.80 (s, 1H, H8), 6.41 (s, 1H, H6); ^13^C NMR (125 MHz, DMSO) *δ* 156.0 (*C*O), 152.1 (C9), 147.0 (C5), 143.1 (C7), 134.8 (C4), 134.6 (C14), 131.8 (C13), 131.0 (C12), 130.9 (q, *J* = 33.3 Hz, C2), 128.7 (C15), 128.6 (C3), 125.3 (C11), 124.5 (C1), 123.1 (q, *J* = 272.5 Hz, *C*F_3_), 122.2 (C10), 96.0 (C6), 87.2 (C8); ^19^F NMR (471 MHz, DMSO) *δ* −61.15 (C*F*_3_); IR (ATR) 3449, 1665, 1606, 1459, 1369, 682.5 cm^−1^; HRMS calcd C_20_H_10_BrF_6_N_3_O m/z: [M + Na]^+^, 523.9911, found [M + Na]^+^ = 523.9807 [Diff (ppm) = 0.55].

##### Cell Culture

The human GBM cell line, U‐251 MG (formerly known as U373 MG), and the human embryonic kidney cell line, HEK293, were kindly gifted by Dr. Michael Carty (Trinity College Dublin) and Dr. Darren Fayne (Trinity College Dublin). Both cell lines were initially purchased from the American Type Culture Collection, ATCC. The absence of mycoplasma was checked by using a MycoAlert PLUS Mycoplasma Detection kit (Lonza). U‐251 MG and HEK293 were cultured and maintained in Dulbecco's modified eagle medium (DMEM)‐high glucose medium (Merck) supplemented with 10% fetal bovine serum (FBS) (Merck) and 1% penicillin–streptomycin antibiotic (Merck) in a 37 °C incubator within a humidified 5% (v/v) CO_2_ atmosphere. Cells were routinely sub‐cultured upon 70–80% confluency using 0.25% w/v Trypsin‐EDTA solution (Merck).

##### Pyrazolopyrimidinone Cell Treatment

Dose–response curves for each of the pyrazolo[1,5‐*α*]pyrimidinones were performed to determine the cytotoxic effect of the compounds on a cancerous, U‐251 MG cell line and a noncancerous, HEK293 cell line. The synthesized pyrazolopyrimidinones were dissolved in DMSO (Merck) to obtain a stock solution which was stored at −20 °C. The working solutions of the pyrazolopyrimidinones did not exceed a DMSO value of 0.05% to avoid the cytotoxic effects of DMSO on the cells. U‐251 MG and HEK293 cells were seeded at a density of 2 x 10^3^ cells per well (100 μL) in a 96‐well plate (Sarstedt) and incubated for 24 h prior to each treatment to allow for cell adhesion. The stock solution was diluted in media to working concentrations of 100, 75, 50, 25, 10, 5, and 0.1 μM. Media on the seeded cells was then replaced with the working concentrations of the compound or the controls in a 96‐well plate (100 μL). The treated plate was incubated at 37 °C in the humidified incubator for 5 days. Cell viability was assessed after 5 days using the MTT assay. Dose–response curves were then plotted relative to the positive control whereby cells were treated 20% DMSO and a negative control whereby cells were maintained in fresh DMEM.

##### Cell Viability Assay

To determine the extent of the cytotoxic effect of the pyrazolopyrimidinone on each cell line, the cell viability at each working concentration was determined via the MTT assay (Merck). The working solution of MTT used was 5 mg MTT per 1 ml of PBS diluted in 9 ml of media. After 5 days of incubation, the treatment was removed from each of the wells and replaced with 100 μL of fresh MTT solution. The plate was then incubated for 3 h at 37 °C in a humidified incubator to allow the reduction of the yellow tetrazolium salt to purple formazan crystals. After the incubation, the MTT solution was removed from the wells and replaced with 100 μL of DMSO for 15 min to dissolve the formazan crystals. The absorbance was read at 570 nm using a spectrophotometric microplate reader (Thermo Fisher Scientific).

##### Evaluation of Cell Death by Flow Cytometry

To evaluate if the HIT compounds are potentially eliciting cell death, flow cytometry using propidium iodide (PI) and JC‐1 dye was used.

##### Propidium Iodine Analysis

Propidium iodide (PI) is a DNA staining, cell membrane impermeable dye which was used to assess cell death via loss of membrane integrity and is used to indicate necrotic or late apoptotic cells. Stock solutions of PI dye (Merck, Ireland) were prepared at 10 μg/mL in PBS and stored at −20 °C. U‐251 MG cells were seeded in 96‐well plates at a seeding density of 2 x 10^3^ cells per well (100 μL), as previously stated. After 24 h, the U‐251 MG cells were treated with fresh drug at the highest toxic concentration, the IC_50_ value, and the highest nontoxic and incubated for 5 days. Subsequently, the compound was collected in 15 ml falcon tubes. Cells were washed with PBS (100 μL), and the PBS was collected in corresponding tubes to ensure all cells are collected. The cells were collected using 0.25% trypsin‐EDTA (50 μL) with a 6‐min incubation and media neutralization (100 μL). Each tube was centrifuged at 1200rpm for 5 min to obtain a pellet. The supernatant was removed, and the pellet was resuspended in PBS and transferred to an Eppendorf tube. Ten microliters of PI stain (2.5 μg/ml) was added to the Eppendorf tubes, and the tubes were incubated for a period of 15 min. Cells were also treated using controls whereby the positive control used was DMEM and 20% DMSO and the negative control used was fresh media. The cells were then analyzed using flow cytometry (Beckmann Coulter, CytExpert version 2.4.0.28). Fluorescence was measured using the FL2 channel. PI was detected in the red channel which is R‐phycoerythrin (PE‐Cy5). PE was used in combination with fluorescein isothiocyanate (FITC) and is excited at a wavelength of 488 nm.

##### JC‐1 Analysis

JC‐1 dye forms aggregates in healthy mitochondria which changes the fluorescent property of the dye. Hence, it is used to assess mitochondrial membrane depolarization and indicate apoptosis and other regulated cell death pathways. Stock solutions of JC‐1 dye (Merck) were prepared at 1 mg/mL in DMSO and stored at −20 °C. U‐251 MG cells were seeded in 6‐well plates (Sarstedt) at a seeding density of 2 x 10^4^ cells per well (1000μL) after which cells were treated with drug concentrations (highest toxic, IC_50_, nontoxic). After 5 days incubation, the compound and wash step with PBS was collected in falcon tubes. The cells were then harvested by trypsinization (500 μL), incubated for a period of 6 min, neutralized with media (1000 μL), and pelleted at 1200rpm for 5 min. A JC‐1 working solution of 2 μg/mL was made up in DMEM and was light protected. The cell pellet was resuspended and stained with 2 μg/mL JC‐1 dye and incubated and protected from the light for a period of 10 min. Cells were then centrifuged at 1200rpm for 5 min. The supernatant was removed, and cells were washed with 1 mL of sterile PBS. This was repeated twice. The cells were resuspended in sterile PBS where the cell pellet was observed to be red in color. Cells were also treated using controls whereby the positive control used was DMEM and 20% DMSO and the negative control used was fresh media. The cells were then analyzed using flow cytometry (Beckmann Coulter, CytExpert version 2.4.0.28). Fluorescence was measured using FL1 (FITC) (530 nm) and FL2 (PE) (595 nm) channels with emission.

##### Statistical Analysis

Each of the experiments was performed at least three independent times, with a minimum of six technical replicates per experiment. Statistical analysis was carried out using GraphPad Prism version 8.0.0 (GraphPad Softwares, Inc.). Curve fitting and the IC_50_ values were obtained through the dose–response curves using nonlinear regression. To determine if the dose–response curves were statistically distinct and not overlapping, extra sum‐of‐squares F‐test was used, and a p‐value was obtained. The effect of compound treatment on both cell lines was statistically compared via two‐way ANOVA with Tukey's post‐test. The CytExpert (Beckman Coulter) software was used for flow cytometry analysis. Graphs represent the % dead cells whereby the total number of dead cells was divided by the total cell population. The statistical significance of the flow cytometry results was determined via one‐way analysis of variance (ANOVA) with Dunnett's T3 multiple comparison post‐test. Data is presented as the mean ± standard error of mean (SEM). Summary of results from statistical analysis reports p‐values as follows: ≥0.05 (ns), 0.01–0.05 (*), 0.001–0.01 (**), 0.001–0.01(***), and <0.0001(****).

## Conflict of Interest

The authors declare no conflicts of interest.

## Supporting information

Supplementary Material

## Data Availability

The data that support the findings of this study are available from the corresponding author upon reasonable request.
