# Supplementary material for: Development of Novel Anticancer Pyrazolopyrimidinones Targeting Glioblastoma
Source: ChemMedChem. 2025 Aug 8;20(20):e202500337. doi: 10.1002/cmdc.202500337 (PMC12530846; doi:10.1002/cmdc.202500337)
Supplement: Supplementary file 1 — Supplementary Material [file CMDC-20-e202500337-s001.pdf]

# Development of Novel Anticancer Pyrazolopyrimidinones Targeting Glioblastoma

Kate Byrne[a,b], Natalia Bednarz[a,c], Ciara McEvoy[a,b], John C. Stephens[b,d], James F. Curtin[a,c] and Gemma K. Kinsella[a,c*]

[a]School of Food Science and Environmental Health, Technological University Dublin, Ireland.

[b]Department of Chemistry, Maynooth University, Ireland.

[c]Sustainability and Health Research Hub, Technological University Dublin, Ireland.

[d]Kathleen Lonsdale Institute for Human Health Research, Maynooth University, Ireland.

***Corresponding Author:** gemma.kinsella@tudublin.ie

## General information

The synthesized compounds were characterized using spectroscopic techniques including $^{1}H$ NMR, $^{13}C$ NMR, 2D NMR, IR and HR-MS. NMR spectra were recorded using Bruker Ascend 500 spectrometer at 293 K operating at 500 MHz for the $^{1}H$ nucleus and 125 MHz for the $^{13}C$ nucleus or a Bruker Advance 300 spectrometer operating at 300 MHz for $^{1}H$ nucleus and 75 MHz for $^{13}C$ nucleus. Peak multiplicities of $^{1}H$-NMR signals were designated as s (singlet), d (doublet), dd (doublet of doublet), t (triplet), q (quartet), m (multiplet) etc. Coupling constants (*J*) are in Hz. The chemical shift denoted in parts per millions (ppm) using reference 0 ppm TMS. The peaks at $\delta$ 3.3 and 2.5 in the $^{1}H$-NMR spectra are from the DMSO-$d^{6}$ solvent. $^{13}C$ NMR spectra were recorded with complete proton decoupling.

Infrared spectra were recorded on a Perkin Elmer Spectrum 100 FT-IR spectrophotometer using a smart endurance single bounce diamond, attenuated total reflection (ATR) cell. Spectra were recorded in the region of 4000–600 $cm^{-1}$ and were obtained by the co-addition of 4 scans with a resolution of 4 $c^{-1}$.

High-resolution mass spectrometry (HR-MS) was performed in the University of Bath (UoB). In UoB, HPLC-ESI-TOF analysis was conducted using an electrospray time-of-flight (MicrOTOF) mass spectrometer (Bruker Daltonik GmbH, Bremen, Germany), which was coupled to an Agilent HPLC stack (Agilent, Santa Clara, CA, United States) consisting of Agilent G1312A binary pump with G1329A autosampler and G1316A column oven. Analyses were performed in ESI positive and negative mode. Data processing was performed using the Compass Data Analysis software scripts (Bruker Daltonik GmbH, Bremen, Germany).

Microwave reactions were carried out using a CEM Discover Microwave Synthesizer with a vertically focused floor mounted infrared temperature sensor, external to the microwave tube. The 10 mL reaction vessels used were supplied from CEM and were made of borosilicate glass. Melting point analyses were carried out using a Stewart Scientific SMP11 melting point apparatus and are uncorrected.

## General procedure of the synthesis of pyrazolopyrimidinones via microwave

A microwave vial (2 mL) was charged with the required 5-aminopyrazole (0.45 mmol, 1 equiv), β-ketoester (0.675 mmol, 1.5 equiv), AcOH (14.3 µL, 0.25 mmol, 0.56 equiv) in MeOH (1 mL) were subjected to MW irradiation (100 W, 150 $^{o}$C) for 2 h. The resulting mixture was concentrated under reduced pressure and the residue was purified via column chromatography or trituration in MeOH/ water to give the title compound. Some compounds; **17**, **23**, where purified further via hot recrystallization in EtOH.

**General procedure of the synthesis of pyrazolopyrimidinones via reflux**

A round-bottom flask (10 mL) was charged with the required 5-aminopyrazole (0.45 mmol, 1 equiv), β-ketoester (0.675 mmol, 1.5 equiv), and AcOH (14.3 µL, 0.25 mmol, 0.56 equiv) in MeOH (3 mL). The reaction mixture was stirred under reflux for 20 h. After completion, the solvent was removed under reduced pressure, and the residue was purified via column chromatography or trituration to give the title compound. Some compounds where purified further via recrystallization in EtOH.

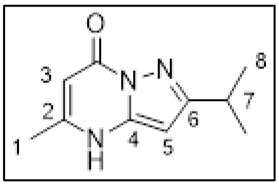


**2-Isopropyl-5-methylpyrazolo[1,5-a]pyrimidin-7(4H)-one (1).** 3-Isopropyl-1H-pyrazol-5-amine (0.169 g, 1.35 mmol, 1 equiv), ethyl 3-oxobutanoate (0.386 g, 2.97 mmol, 2.2 equiv), AcOH (42.9 µL, 0.75 mmol, 0.56 equiv) and MeOH (4 mL) were subjected to MW irradiation (100 W, 150 $^{o}$C) for 2 h. The MeOH was removed under reduced pressure to leave a residue and the residue was purified via precipitation with MeOH (1 mL) and water (3 mL) to give the title compound. Yield 0.147 g, 57%; $R_f$ 0.65 (EtOAc); $^{1}$H NMR (500 MHz, DMSO) $\delta$ 12.11 (s, 1H, N*H*), 5.92 (s, 1H, H5), 5.49 (s, 1H. H3), 2.99 – 2.90 (Hept, 1H, H7), 2.25 (s, 3H, H1), 1.22 (d, *J* = 6.9 Hz, 6H, H8); $^{13}$C NMR (125 MHz, DMSO) $\delta$ 161.6 (C6), 156.6 (*C*O), 149.6 (C2), 141.9 (C4), 94.9 (C4), 85.7 (C5), 28.0 (C7), 22.3 (C8), 18.6 (C1); IR (ATR) 2962, 2169, 2039, 1577, 1408, 1285, 1178 cm$^{-1}$; HR-MS calcd for $C_{10}H_{13}N_3O$ m/z: $[M+Na]^+$, 214.1059, found $[M+H]^+$ = 214.0951 [Diff (ppm) = -0.07].

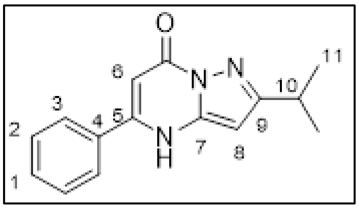


**2-Isopropyl-5-phenylpyrazolo[1,5-a]pyrimidin-7(4H)-one (2).** 3-Isopropyl-1H-pyrazol-5-amine (0.169 g, 1.35 mmol, 1 equiv), ethyl 3-oxo-3-phenylpropanoate (0.380 g, 1.98 mmol, 1.5 equiv), AcOH (42.9 µL, 0.75 mmol, 0.56 equiv) and MeOH (3 mL) were subjected to MW irradiation (100 W, 150 $^{o}$C) for 2 h. The product precipitated out in ice cold MeOH (3 mL) to give the title compound. Yield 0.042 g, 27%; R$_f$ 0.73 (EtOAc); $^{1}$H NMR (400 MHz, DMSO) *δ* 12.35 (s, 1H, N*H*), 7.86 – 7.77 (m, 2H, H3), 7.58 (d, *J* = 5.2 Hz, 3H, H1 & H2), 6.05 (s, 1H, H8), 5.99 (s, 1H, H6), 3.00 (dd, *J* = 13.7, 6.9 Hz, 1H, H10), 1.27 (d, *J* = 6.9 Hz, 6H, H11); $^{13}$C NMR (125 MHz, DMSO) *δ* 162.0 (C9), 156.3 (*C*O), 149.3 (C5), 142.2 (C7), 132.4 (C4), 131.0 (C1), 129.1 (C2), 127.2 (C3), 93.7 (C6), 86.6 (C8), 28.0 (C10), 22.2 (C11); IR (ATR) 3248, 3129, 3060, 2969, 2324, 1661, 1604, 1471, 1426, 1471, 1426, 1290 cm$^{-1}$; HR-MS calcd for $C_{15}H_{15}N_3O$ m/z: [M+H]$^+$, 254.1288, found [M+H]$^+$ = 254.1288 [Diff (ppm) = 0.37].

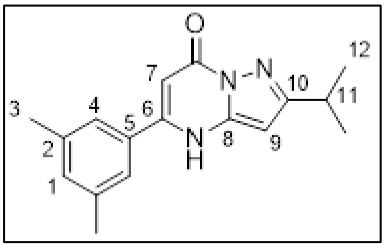


**5-(3,5-Dimethylphenyl)-2-isopropylpyrazolo[1,5-a]pyrimidin-7(4H)-one (3).** 3-Isopropyl-1H-pyrazol-5-amine (0.169 g, 1.35 mmol, 1 equiv), β- ethyl 3-(3,5-dimethylphenyl)-3-oxopropanoate (0.651 g, 2.97 mmol, 2.2equiv), AcOH (42.9 µL, 0.75 mmol, 0.56 equiv) and MeOH (4 mL) were subjected to MW irradiation (100 W, 150 $^{o}$C) for 2 h. The resulting product crystalised in ice cold MeOH (3 mL) and was obtained as a white solid. Yield 0.118 g, 31%; R$_f$ 0.8 (EtOAc); $^{1}$H NMR (500 MHz, DMSO) *δ* 12.29 (s, 1H, N*H*), 7.42 (s, 2H, H4), 7.21 (s, 1H, H1), 6.03 (s, 1H, H9), 5.95 (s, 1H, H7), 3.00 (hept, *J* = 6.9 Hz, 1H, H11), 2.36 (s, 6H, H3), 1.26 (d, *J* = 6.9 Hz, 6H, H12); $^{13}$C NMR (125 MHz, DMSO) *δ* 162.0 (C10), 156.4 (*C*O), 149.5 (C6), 142.2 (C8), 138.4 (C9), 132.4 (C1 & C5), 124.8 (C4), 93.4 (C7), 86.5 (C9), 28.0 (C11), 22.4 (C12), 20.9 (C3); IR (ATR) 3200, 3077, 2962, 2301, 2179, 2038, 1594, 1440, 1290 cm$^{-1}$; HR-MS calcd for $C_{17}H_{19}N_3O$ m/z: [M+H]$^+$, 282.1528, found [M+H]$^+$ = 282.1600 [Diff (ppm) = -0.18].

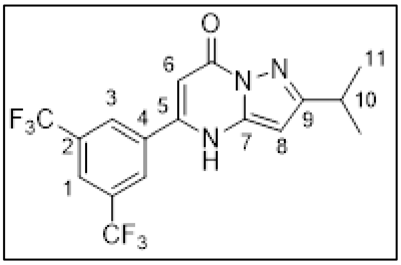


**5-(3,5-Bis(trifluoromethyl)phenyl)-2-isopropylpyrazolo[1,5-a]pyrimidin-7(4H)-one (4).** 3-Isopropyl-1H-pyrazol-5-amine (0.1689 g, 1.35 mmol, 1 equiv), ethyl 3-(3,5-bis(trifluoromethyl)phenyl)-3-oxopropanoate, (0.664 g, 2.03 mmol, 1.5 equiv), AcOH (42.9 µL, 0.75 mmol, 0.56 equiv) in MeOH (4 mL). These were reacted in MeOH (3 mL) via reflux for 20 hours. The resulting product precipitated out in ice cold MeOH (2 mL) to give the title compound. Yield 0.130 g, 25%; $R_f$ 0.28 (EtOAc); $^1$H NMR (500 MHz, DMSO) *δ* 8.48 (s, 2H, H2), 8.30 (s, 1H, H1), 6.26 (s, 1H, H6), 6.11 (s, 1H, H8), 3.04 – 2.97 (m, 1H, H10), 1.25 (d, *J* = 6.8 Hz, 6H, H11); $^{13}$C NMR (125 MHz, DMSO) *δ* 162.7 (C9), 156.4 (<u>C</u>O), 146.8 (C5), 142.5 (C7), 135.3 (C4), 131.2 (q, *J* = 33.3 Hz, C2), 128.6 (C3), 124.5 (C1), 123.2 (q, *J* = 273.1 Hz, <u>C</u>$F_3$), 95.6 (C6), 87.2 (C8), 28.2 (C10), 22.5 (C11); $^{19}$F NMR (471 MHz, DMSO) *δ* -61.27 (C<u>*F*</u>$_3$); IR (ATR) 3095, 2974, 1613, 1369, 1277, 1129, 902.4 cm$^{-1}$; HR-MS calcd for $C_{17}H_{13}F_6N_3O$ m/z: [M+H]$^+$, 390.0963, found [M+H]$^+$ = 390.1042 [Diff (ppm) = 1.87].

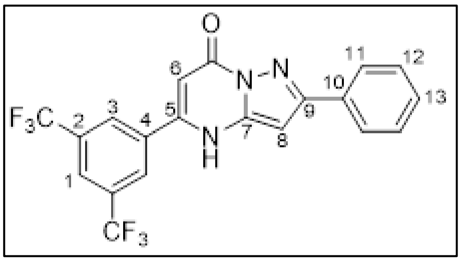


**5-(3,5-Bis(trifluoromethyl)phenyl)-2-phenylpyrazolo[1,5-a]pyrimidin-7(4H)-one (5).** 3-Phenyl-1H-pyrazol-5-amine7 (0.143 g, 0.9 mmol, 1 equiv), ethyl 3-(3,5-bis(trifluoromethyl)phenyl)-3-oxopropanoate (0.650 g, 1.98 mmol, 2.2 equiv), AcOH (42.9 µL, 0.75 mmol, 0.56 equiv) in MeOH (3 mL) were subjected to MW irradiation (100 W, 150 $^o$C) for 2 h. The MeOH was removed under reduced pressure to leave a residue and the residue was purified via precipitation with MeOH (1.5 mL) This solid underwent hot recrystallization using EtOH (3 mL) to give the title compound. $^1$H NMR (500 MHz, DMSO) *δ* 12.86 (s, 1H, N<u>*H*</u>), 8.55 (s, 2H, H3), 8.35 (s, 1H, H1), 8.02 (d, *J* = 7.2 Hz, 2H, H11), 7.50 (t, *J* = 7.4 Hz, 2H, H12), 7.44 (d, *J* = 7.3 Hz, 1H, H13), 6.72 (s, 1H, H8), 6.40 (s, 1H, H6); $^{13}$C NMR (125 MHz, DMSO) *δ* 156.5 (<u>C</u>O), 154.0 (C9), 147.3 (C5), 143.6 (C7), 135.4 (C4), 132.7 (C10), 131.34 (q, *J* = 33.3 Hz, C2), 129.5 (C13), 129.3 (C12), 129.0 (C3), 126.7 (C11), 124.9 (C1), 123.5 (q, *J* = 546.4, 273.1 Hz, <u>C</u>$F_3$), 96.3 (C6), 87.3 (C8); $^{19}$F NMR (471 MHz, DMSO) *δ* -61.16 (C<u>*F*</u>$_3$); IR (ATR) 3517, 3131, 2891, 1605, 1370, 1276, 1122 cm$^{-1}$; HR-MS calcd for $C_{20}H_{11}F_6N_3O$ m/z: [M+H]$^+$, 424.0885, found [M+H]$^+$ = 424.0806 [Diff (ppm) = 1.31].

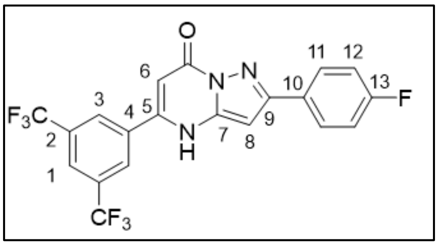


**5-(3,5-Bis(trifluoromethyl)phenyl)-2-(4-fluorophenyl)pyrazolo[1,5-a]pyrimidin-7(4H)-one (6).** 3-(4-Fluorophenyl)-1H-pyrazol-5-amine (0.1594 g, 0.9 mmol, 1 equiv), ethyl 3,3,5-bis(trifluoromethyl)phenyl)-3-oxopropanoate (0.650 g, 1.98 mmol, 2.2 equiv), AcOH (42.9 µL, 0.75 mmol, 0.56 equiv) and MeOH (3 mL) were subjected to MW irradiation (100 W, 150 $^{o}$C) for 2 h. The resulting product precipitated out in methanol (3 mL) and to give the title compound. Yield 0.162 g, 82%; $R_f$ 0.78 (EtOAc); $^{1}$H NMR (500 MHz, DMSO) $\delta$ 12.85 (s, 1H, N*H*), 8.55 (s, 2H, H3), 8.35 (s, 1H, H1), 8.07 (m, 2H, H11), 7.32 (dd, $J$ = 12.3, 5.4 Hz, 2H, H12), 6.71 (s, 1H, H8), 6.40 (s, 1H, H6); $^{13}$C NMR (125 MHz, DMSO) $\delta$ 162.67 (d, $J$ = 245.9 Hz, C13), 156.0 (*C*O), 152.7 (C9), 146.8 (C5), 143.1 (C7), 134.8 (C4), 130.9 (q, $J$ = 33.3 Hz, C2), 128.8 (C10), 128.6 (C3), 128.4 (d, $J$ = 8.4 Hz, C11), 124.4 (C1), 123.1 (q, $J$ = 273.0 Hz, *C*F3), 115.72 (d, $J$ = 21.6 Hz, C12), 96.0 (C6), 86.7 (C8); $^{19}$F NMR (471 MHz, DMSO) $\delta$ -61.16 (C*F*3), -112.55 (dd, $J$ = 9.8, 4.4 Hz, F); IR (ATR) 3094, 2168, 1670, 1285, 1125, 847.1, 683.8, 401.6 cm$^{-1}$; HR-MS calcd for $C_{20}H_{10}F_7N_3O$ m/z: [M+H]$^+$, 442.0712, found [M+H]$^+$ = 442.0788 [Diff (ppm) = 0.71].

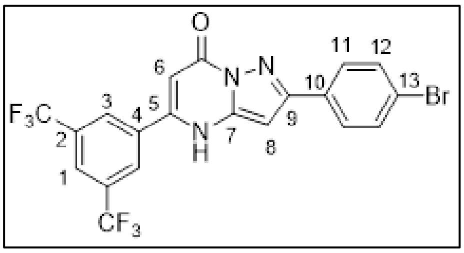


**5-(3,5-Bis(trifluoromethyl)phenyl)-2-(4-bromophenyl)pyrazolo[1,5-a]pyrimidin-7(4H)-one (7).** 3-(4-Bromophenyl)-1H-pyrazol-5-amine, (0.174 g, 0.9 mmol, 1 equiv), ethyl 3-(3,5-bis(trifluoromethyl)phenyl)-3-oxopropanoate (0.650 g, 1.98 mmol, 2.2 equiv), AcOH (42.9 µL, 0.75 mmol, 0.56 equiv) and MeOH (3 mL) were subjected to MW irradiation (100 W, 150 $^{o}$C) for 2 h. The resulting mixture was purified using hot filtration with MeOH (3 mL), to give the title compound. Yield 0.287 g, 64%; Rf 0.48 (70:30 Pet Ether/ EtOAc 1:1 v/v); $^{1}$H NMR (500 MHz, DMSO) $\delta$ 12.88 (s, 1H, N*H*), 8.54 (s, 2H, H3), 8.35 (s, 1H, H1), 7.98 (d, $J$ = 8.4 Hz, 2H, H11), 7.68 (d, $J$ = 8.4 Hz, 2H, H12), 6.74 (s, 1H, H8), 6.41 (s, 1H, H6); $^{13}$C NMR (125 MHz, DMSO) $\delta$ 156.4 (*C*O), 153.0 (C9), 147.3 (C5), 143.5 (C7), 135.3 (C4), 132.2 (C12), 132.0 (C10), 131.3 (q, J = 25 Hz, C2), 129.1 (C3), 128.8 (C11), 125.0 (C1), 123.5 (q, $J$ = 273.2 Hz, *C*F3), 96.5 (C6), 87.4 (C8); $^{19}$F NMR (471 MHz, DMSO) $\delta$ -61.31 (d, J = 141.6 Hz, C*F*3); IR (ATR) 3490, 3436, 3136, 1656, 1606, 1428, 1371, 1277, 1111, 681

cm$^{-1}$; HR-MS calcd for $C_{20}H_{-10}BrF_6N_3ONa$ m/z: [M + Na]+, 523.9911; found [M + Na]+ = 523.9805 [Diff (ppm) = 0.18].

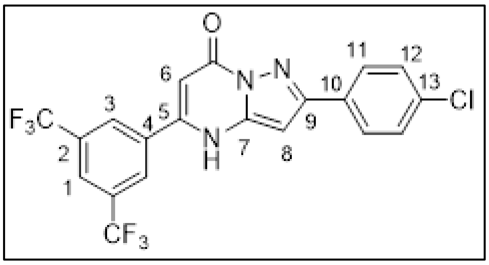


**5-(3,5-Bis(trifluoromethyl)phenyl)-2-(4-chlorophenyl)pyrazolo[1,5-a]pyrimidin-7(4H)-one (8).** A microwave vial was charged with the required 3-(4-chlorophenyl)-1H-pyrazol-5-amine (0.1737 g, 0.9 mmol, 1 equiv), ethyl 3-(3,5-bis(trifluoromethyl)phenyl)-3-oxopropanoate (0.650 g, 1.98 mmol, 2.2 equiv), AcOH (42.9 µL, 0.75 mmol, 0.56 equiv) and MeOH (3 mL) were subjected to MW irradiation (100 W, 150 °C) for 2 h. The resulting product precipitated out in methanol (3 mL) and to give the title compound. Yield 0.203 g, 99%; $R_f$ 0.68 (EtOAc); $^1$H NMR (500 MHz, DMSO) $\delta$ 12.87 (s, 1H, N*H*), 8.54 (s, 2H, H3), 8.35 (s, 1H, H1), 8.04 (d, *J* = 8.5 Hz, 2H, H11), 7.55 (d, *J* = 8.5 Hz, 2H, H12), 6.74 (s, 1H, H8), 6.41 (s, 1H, H6); $^{13}$C NMR (125 MHz, DMSO) $\delta$ 156.0 (*C*O), 152.5 (C9), 146.8 (C5), 143.1 (C7), 134.8 (C4), 133.7 (C10), 131.1 (q, *J* = 16.25 Hz, C2), 130.7 (C13), 128.8 (C12), 128.6 (C3), 128.0 (C11), 124.5 (C1), 123.1 (q, *J* = 16.25 Hz, 271.25 Hz, *C*F$_3$), 96.0 (C6), 87.0 (C8); $^{19}$F NMR (471 MHz, DMSO) $\delta$ -61.16 (C*F*$_3$); IR (ATR) 3489, 3431, 3137, 1656, 1606, 1430, 1371, 1277, 1112, 681 cm$^{-1}$; HR-MS calcd for $C_{20}H_{10}ClF_6N_3O$ m/z: [M+H]$^+$, 458.0417; found [M+H]$^+$ = 458.0490 [Diff (ppm) =0.47].

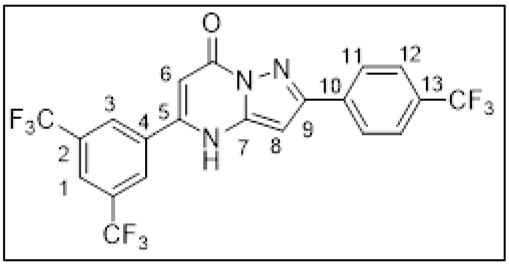


**5-(3,5-Bis(trifluoromethyl)phenyl)-2-(4-(trifluoromethyl)phenyl)pyrazolo[1,5-a]pyrimidin-7(4H)-one (9).** 3-(4-(Trifluoromethyl)phenyl)-1H-pyrazol-5-amine (0.204 g, 0.9 mmol, 1 equiv), ethyl 3-(3,5-bis(trifluoromethyl)phenyl)-3-oxopropanoate (0.650 g, 1.98 mmol, 2.2 equiv), AcOH (42.9 µL, 0.75 mmol, 0.56 equiv) and MeOH (3 mL) were subjected to MW irradiation (100 W, 150 °C) for 2 h. The resulting product precipitated out in methanol (3 mL) to give the title compound. Yield 0.084 g, 38%; $R_f$ 0.65 (EtOAc); $^1$H NMR (500 MHz, DMSO) $\delta$ 12.92 (s, 1H, N*H*), 8.54 (s, 2H, H3), 8.34 (s, 1H, H1), 8.23 (d, *J* = 8.1 Hz, 2H, H11), 7.83 (d, *J* = 8.3 Hz, 2H, H12), 6.81 (s, 1H, H8), 6.41 (s, 1H, H6); $^{13}$C NMR

(125 MHz, DMSO) *δ* 156.0 (*C*O), 152.0 (C9), 147.0 (C5), 143.2 (C7), 136.2 (C10), 134.8 (C4), 130.9 (q, *J* = 33.2 Hz, C2), 129.1 (q, *J* = 25 Hz, C13), 128.6 (C3), 126.9 (C11), 125.7 (d, *J* = 3.8 Hz, C12), 124.5 (C1), 124.3 (q, *J* = 287.5 Hz, $\underline{C}F_3$), 123.08 (q, *J* = 272.9 Hz, 2$\underline{C}F_3$), 96.1 (C6), 87.5 (C8); $^{19}$F NMR (471 MHz, DMSO) *δ* -61.21 ($C\underline{F}_3$); IR (ATR) 3066, 3021, 2969, 2926, 2152, 2019, 1668, 1601,1515, 1437, 1274, 1129, 681 cm$^{-1}$; HR-MS calcd for $C_{12}H_{10}F_9N_3O$ m/z: [M + H]$^+$, 492.0680; found [M + H]$^+$ = 492.0753 [Diff (ppm) = 0.08].

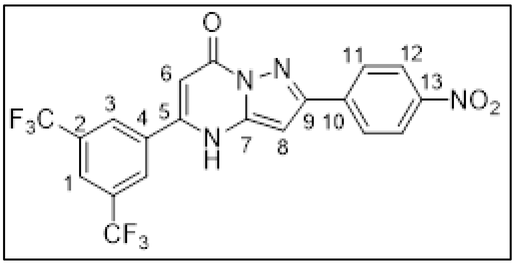


**5-(3,5-Bis(trifluoromethyl)phenyl)-2-(4-nitrophenyl)pyrazolo[1,5-a]pyrimidin-7(4H)-one (10).** 3-(4-Nitrophenyl)-1H-pyrazol-5-amine (0.1837 g, 0.9 mmol, 1 equiv), ethyl 3-(3,5-bis(trifluoromethyl)phenyl)-3-oxopropanoate (0.650 g, 1.98 mmol, 2.2 equiv), AcOH (42.9 µL, 0.75 mmol, 0.56 equiv) and MeOH (6 mL) were subjected to MW irradiation (100 W, 150 $^o$C) for 2 h. The resulting product precipitated out in MeOH (3 mL) and to give the title compound. Yield 0.3684 g, 87%; $R_f$ 0.60 (EtOAc); $^1$H NMR (500 MHz, DMSO) *δ* 12.95 (s, 1H, N*H*), 8.54 (s, 2H, H3), 8.35 (s, 1H, H1), 8.29 (q, *J* = 9.0 Hz, 4H, H11 & H12), 6.86 (s, 1H, H8), 6.43 (s, 1H, H6); $^{13}$C NMR (125 MHz, DMSO) *δ* 155.9 (*C*O), 151.4 (C9), 147.5 (C13), 147.1 (C5), 143.2 (C7), 138.5 (C10), 134.7 (C4), 130.9 (q, *J* = 33.3 Hz, C2), 128.6 (C3), 127.3 (C11), 124.5 (C1), 124.0 (C12), 123.1 (q, *J* = 273.3 Hz, $\underline{C}F_3$), 96.1 (C6), 88.0 (C8); $^{19}$F NMR (471 MHz, DMSO) *δ* -61.19 ($C\underline{F}_3$); IR (ATR) 3131, 2218, 2162, 2021, 1675, 1606, 1369, 1321, 1276, 1128, 681 cm$^{-1}$; HR-MS calcd for $C_{20}H_{10}F_6N_4O_3$ m/z: [M+H]$^+$, 469.0657, found [M+H]$^+$ = 469.0734 [Diff (ppm) = 1.07].

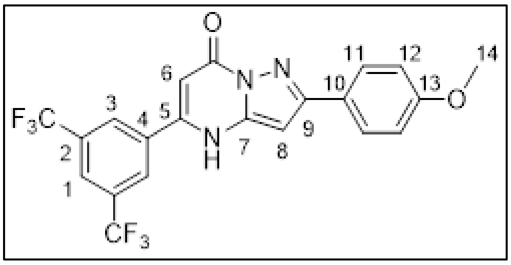


**5-(3,5-Bis(trifluoromethyl)phenyl)-2-(4-methoxyphenyl)pyrazolo[1,5-a]pyrimidin-7(4H)-one (11).** A microwave vial was charged with the required 3-(4-methoxyphenyl)-1H-pyrazol-5-amine (0.17g, 0.9 mmol, 1 equiv), ethyl 3-(3,5-bis(trifluoromethyl)phenyl)-3-oxopropanoate, (0.650 g, 1.98mmol, 2.2 equiv), AcOH (42.9 µL, 0.75 mmol, 0.56 equiv) and MeOH (3 mL) were subjected to

MW irradiation (100 W, 150 °C) for 2 h. The resulting product precipitated out in methanol (3 mL) to give the title compound. Yield 0.208 g, 51%; $R_f$ 0.64 (EtOAc); $^1$H NMR (500 MHz, DMSO) $\delta$ 12.79 (s, 1H, N*H*), 8.55 (s, 2H, H3), 8.35 (s, 1H, H1), 7.95 (d, *J* = 8.4 Hz, 2H, H11), 7.05 (d, *J* = 8.3 Hz, 2H, H12), 6.63 (s, 1H, H8), 6.38 (s, 1H, H6), 3.82 (s, 3H, H14); $^{13}$C NMR (125 MHz, DMSO) $\delta$ 160.0 (C13), 156.0 (*C*O), 153.5 (C9), 146.5 (C5), 142.9 (C7), 134.9 (C4), 130.9 (q, *J* = 33.4 Hz, C2), 128.5 (C3), 127.7 (C11),.124.7 (C10), 124.4 (C1), 123.1 (q, *J* = 273.0 Hz, $\underline{C}F_3$), 114.2 (C12), 95.9 (C6), 86.3 (C8), 55.2 (C14); $^{19}$F NMR (471 MHz, DMSO) $\delta$ -61.16 ($C\underline{F}_3$); IR (ATR) 3444, 3128, 2954, 1607, 1121, 681 cm$^{-1}$; HR-MS calcd for $C_{21}H_{13}F_6N_3O_2$ m/z: $[M + H]^+$, 454.0912; found $[M + H]^+$= 454.0986 [Diff (ppm) = 0.41].

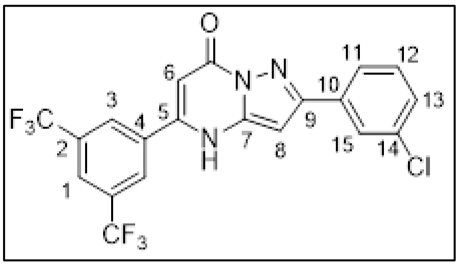


**5-(3,5-Bis(trifluoromethyl)phenyl)-2-(3-chlorophenyl)pyrazolo[1,5-a]pyrimidin-7(4H)-one (12).** 3-(3-Chlorophenyl)-1H-pyrazol-5-amine (0.1737g, 0.9 mmol, 1 equiv), ethyl 3-(3,5-bis(trifluoromethyl)phenyl)-3-oxopropanoate (0.650 g, 1.98 mmol, 2.2 equiv), AcOH (42.9 µL, 0.75 mmol, 0.56 equiv) and MeOH (3 mL) were subjected to MW irradiation (100 W, 150 °C) for 2 h. The resulting product precipitated out in MeOH (3 mL) to give the title compound. Yield 0.312 g, 76%; $R_f$ 0.74 (EtOAc); $^1$H NMR (500 MHz, DMSO) $\delta$ 12.89 (s, 1H, N*H*), 8.52 (s, 2H, H3), 8.32 (s, 1H, H1), 8.01 (s, 1H, H15), 7.95 (d, *J* = 7.4 Hz, 1H, H11), 7.53 – 7.42 (m, 2H, H12 & H13), 6.76 (s, 1H, H8), 6.36 (s, 1H,H6); $^{13}$C NMR (125 MHz, DMSO) $\delta$ 156.0 (*C*O), 152.2 (C9), 146.9 (C5), 143.1 (C4), 134.8 (C7), 134.4 (C10), 133.7 (C14), 130.9 (q, *J* = 33.4 Hz, C2), 130.7 (C12), 128.8 (C13), 128.6 (C3), 125.8 (C15), 124.9 (C11), 124.5 (C1), 123.1 (q, *J* = 272.9 Hz, $\underline{C}F_3$), 96.0 (C6), 87.2 (C8); $^{19}$F NMR (471 MHz, DMSO) $\delta$ -61.22 ($C\underline{F}_3$); IR (ATR) 3510, 3445, 3143, 3066, 2814, 2165, 1664, 1605, 1282, 1124 cm$^{-1}$; HR-MS calcd for $C_{20}H_{10}ClF_6N_3O$ m/z: $[M+H]^+$, 458.0417, found $[M+H]^+$ = 458.0486 [Diff (ppm) =-0.64].

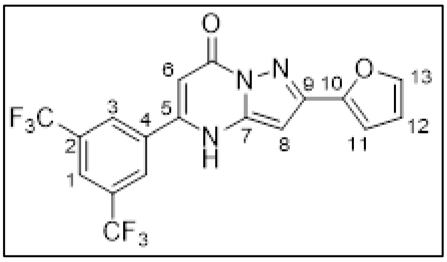


**5-(3,5-Bis(trifluoromethyl)phenyl)-2-(furan-2-yl)pyrazolo[1,5-a]pyrimidin-7(4H)-one (13).** 3-(Furan-2-yl)-1H-pyrazol-5-amine (0.1342 g, 0.9 mmol, 1 equiv), ethyl 3-(3,5-

bis(trifluoromethyl)phenyl)-3-oxopropanoate (0.650 g, 1.98 mmol, 2.2 equiv), AcOH (42.9 µL, 0.75 mmol, 0.56 equiv) in MeOH (3 mL). These were reacted in MeOH (3 mL) via reflux for 20 hours. The resulting product precipitated out in ice cold MeOH (2 mL) to give the title compound. Yield 0.089 g, 24%; $R_f$ 0.26 (EtOAc); $^1$H NMR (500 MHz, DMSO) *δ* 8.50 (s, 2H, H3), 8.33 (s, 1H, H1), 7.81 (s, 1H, H13), 7.04 (s, 1H, H11), 6.65 (s, 1H, H12), 6.49 (s, 1H, H6), 6.36 (s, 1H, H8); $^{13}$C NMR (125 MHz, DMSO) *δ* 156.3 (C̲O), 147.7 (C9), 147.2 (C10), 146.3 (C5), 144.1 (C13), 143.0 (C4), 135.1 (C7), 131.2 (q, *J* = 33.3 Hz, C2), 128.7 (C3), 124.7 (C1), 123.2 (q, *J* = 273.2 Hz, $\underline{C}F_3$), 112.2 (C12), 109.1 (C11), 96.1 (C8), 86.6 (C6); $^{19}$F NMR (471 MHz, DMSO) *δ* -61.23 ($C\underline{F}_3$); IR (ATR) 3169, 3080, 1603, 1367, 1278, 1129, 899.9, 687.1, 538.7 cm$^{-1}$; HR-MS calcd for $C_{18}H_9F_6N_3O_2$ m/z: [M+H]$^+$, 414.0599, found [M+H]$^+$ = 414.0670 [Diff (ppm) = -0.51].

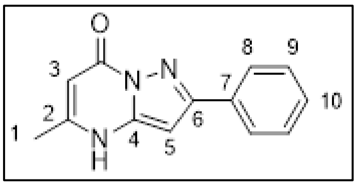

**5-Methyl-2-phenylpyrazolo[1,5-a]pyrimidin-7(4H)-one (14).** 3-Phenyl-1H-pyrazol-5-amine (0.248 g, 1.56 mmol, 1 equiv), ethyl 3-oxobutanoate (0.526 g, 4.05 mmol, 2.6 equiv), AcOH (42.9 µL, 0.75 mmol, 0.48 equiv) and MeOH (4 mL) subjected to MW irradiation (100 W, 150 °C) for 2 h. The resulting product crystalised in cold methanol (3 mL) to give the title compound. Yield 0.232 g, 77%; $R_f$ 0.63 (EtOAc); $^1$H NMR (500 MHz, DMSO) *δ* 12.34 (s, 1H, N*H̲*), 7.97 (d, *J* = 7.4 Hz, 2H, H8), 7.46 (t, *J* = 7.5 Hz, 2H, H9), 7.40 (t, *J* = 7.3 Hz, 1H, H10), 6.57 (s, 1H, H5), 5.60 (s, 1H, H3), 2.30 (s, 3H, H1); $^{13}$C NMR (125 MHz, DMSO) *δ* 156.2 (C̲O), 152.9 (C6), 150.2 (C2), 142.8 (C4), 132.9 (C7), 128.8 (C10), 128.7 (C9), 126.1 (C8), 95.3 (C3), 85.4 (C5), 18.6 (C1); IR (ATR) 3141, 3032, 2881, 2815, 2323, 1573, 1418, 1332, 1219, 1140 cm$^{-1}$; HR-MS calcd for $C_{13}H_{11}N_3O$ m/z: [M+H]$^+$, 226.0902, found [M+H]$^+$ = 226.0973 [Diff (ppm) =-0.48].

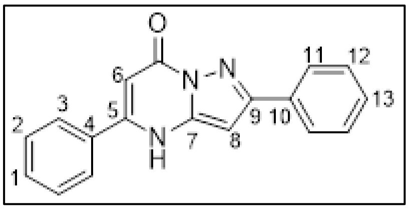

**2,5-Diphenylpyrazolo[1,5-a]pyrimidin-7(4H)-one (15).** 3-Phenyl-1H-pyrazol-5-amine (0.215 g, 1.35 mmol, 1 equiv), ethyl 3-oxo-3-phenylpropanoate (0.380 g, 1.98 mmol, 1.5 equiv), AcOH (42.9 µL, 0.75 mmol, 0.56 equiv) and MeOH (3 mL) were subjected to MW irradiation (100 W, 150 °C) for 2 h. The product precipitated out in cold MeOH (3 mL) to give the title compound. Yield 0.114 g, 30%; $R_f$ 0.76 (EtOAc); $^1$H NMR (500 MHz, DMSO) *δ* 12.60 (s, 1H, N*H̲*), 8.01

(d, *J* = 7.2 Hz, 2H, H11), 7.89 – 7.83 (m, 2H, H3), 7.63 – 7.56 (m, 3H, H1 & H2), 7.49 (t, *J* = 7.4 Hz, 2H, H12), 7.42 (t, *J* = 7.3 Hz, 1H, H13), 6.66 (s, 1H, H8), 6.10 (s, 1H, H6); $^{13}$C NMR (125 MHz, DMSO) *δ* 156.3 (<u>*C*</u>O), 153.3 (C9), 149.6 (C5), 143.1 (C7), 132.4 (C10), 132.3 (C4), 131.1 (C1), 129.1 (C2), 129.0 (C13), 128.8 (C12), 127.3 (C3), 126.2 (C11), 94.1 (C6), 86.6 (C8); IR (ATR) 3256, 3124, 3029, 2908, 1667, 1598, 1442, 1141, 762, 689 cm$^{-1}$; HR-MS calcd for $C_{11}H_{15}N_3O$ m/z: $[M+H]^+$, 310.1059, found $[M+H]^+$ = 310.0950 [Diff (ppm) =-0.22].

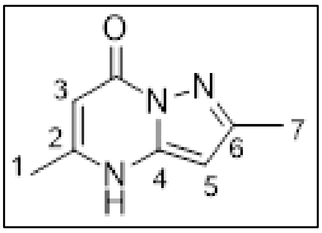


**2,5-Dimethylpyrazolo[1,5-a]pyrimidin-7(4H)-one (16).** 3-Methyl-1H-pyrazol-5-amine (0.175 g, 1.8 mmol, 1 equiv), ethyl 3-oxobutanoate (0.515 g, 3.96 mmol, 2.2 equiv), AcOH (85.8 µL, 0.75 mmol, 0.56 equiv) and MeOH (6 mL) were subjected to MW irradiation (100 W, 150 $^{o}$C) for 2 h. The MeOH was removed under reduced pressure to leave a residue and the residue was purified via precipitation with MeOH (1 mL) to give the title compound. This was repeated three times to achieve the final yield. Yield 0.147 g, 50%; $R_f$: 0.56 (EtOAc); $^1$H NMR (500 MHz, DMSO) *δ* 12.09 (s, 1H, N<u>*H*</u>), 5.90 (s, 1H, H5), 5.48 (s, 1H, H3), 2.25 (d, *J* = 3.2 Hz, 6H, H1 & H7); $^{13}$C NMR (125 MHz, DMSO) *δ* 156.0 (<u>*C*</u>O), 151.7 (C6), 149.6 (C2), 142.1 (C4), 94.9 (C3), 88.0 (C5), 18.5 (C1), 14.1 (C7); IR (ATR) 2824, 2152, 2039, 1626, 1331, 1132, 1006 cm$^{-1}$; HR-MS calcd for $C_8H_9N_3O$ m/z: $[M+H]^+$, 186.0746, found $[M+H]^+$ = 186.0637 [Diff (ppm) = 0.07].

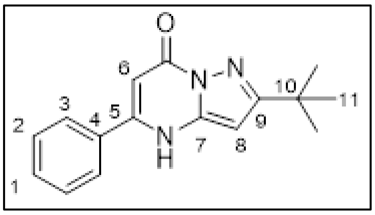

**2-(Tert-butyl)-5-phenylpyrazolo[1,5-a]pyrimidin-7(4H)-one (17).** A microwave vial was charged with the required 3-(tert-butyl)-1H-pyrazol-5-amine (0.188 g, 1.35 mmol, 1 equiv), ethyl 3-oxo-3-phenylpropanoate (0.778 g, 4.05 mmol, 3 equiv), AcOH (64.35 µL, 1.125 mmol, 0.83 equiv) and MeOH (4.5 mL) were subjected to MW irradiation (100 W, 150 $^{o}$C) for 2 h. The MeOH was removed under reduced pressure to leave a residue and the residue was purified via precipitation with MeOH (1.5 mL) followed by $H_2O$ (3 mL) to give the title compound. This solid underwent hot recrystallization using EtOH (3 mL) to give a white solid. Yield 0.262 g, 73%; Rf 0.73 (EtOAc); $^1$H NMR (500 MHz, DMSO) *δ* 12.35 (s, 1H, N<u>*H*</u>), 7.82 (d, J =

5.1 Hz, 2H, H3), 7.59 (s, 3H, H1 & H2 ), 6.08 (s, 1H, H8), 5.99 (s, 1H, H6), 1.32 (s, 9H, H11); $^{13}$C NMR (125 MHz, DMSO) $\delta$ 164.8 (C9), 156.4 (<u>*C*</u>O), 148.9 (C5), 142.2 (C7), 132.5 (C4), 131.0 (C1), 129.1 (C2), 127.2 (C3), 93.7 (C6), 86.0 (C8), 32.5 (C10), 30.1 (C11); IR (ATR) 2956, 2457, 2153, 1672, 1652, 1323, 972.3, 694.9, 527.9 cm$^{-1}$; HR-MS calcd for $C_{16}H_{17}N_3O$ m/z: [M+H]+, 268.1372, found [M+H]+ = 268.1445 [Diff (ppm) = 0.30].

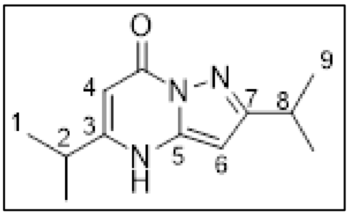


**2,5-Diisopropylpyrazolo[1,5-a]pyrimidin-7(4H)-one (18).** 3-Isopropyl-1H-pyrazol-5-amine (0.225 g, 1.8 mmol, 1 equiv), ethyl 4-methyl-3-oxopentanoate (0.626 g, 3.96 mmol, 2.2 equiv), AcOH (85.8 µL, 0.75 mmol, 0.56 equiv) and MeOH (6 mL) were subjected to MW irradiation (100 W, 150 $^{o}$C) for 2 h. The resulting product precipitated out in ice cold MeOH (3 mL) to give the title compound. Yield 0.293 g, 74%; $R_f$ 0.7 (EtOAc); $^{1}$H NMR (500 MHz, DMSO) $\delta$ 11.96 (s, 1H, N<u>*H*</u>), 5.94 (s, 1H, H6), 5.52 (s, 1H, H4), 3.00 – 2.92 (m, 1H, H8), 2.85 – 2.76 (m, 1H, H2), 1.23 (d, *J* = 6.9 Hz, 12H, H1 & H9); $^{13}$C NMR (125 MHz, DMSO) $\delta$ 161.7 (C7), 158.6 (C3), 156.6 (<u>*C*</u>O), 142.0 (C5), 91.8 (C4), 85.5 (C6), 31.6 (C2), 28.0 (C8), 22.4 (C9), 21.1 (C1); IR (ATR) 3166, 3075, 2962, 2313, 2193, 2038, 1575, 1415,1289, 1178 cm$^{-1}$; HR-MS calcd for $C_{12}H_{17}N_3O$ m/z: $[M+H]^+$, 220.1372, found $[M+H]^+$ = 220.1445 [Diff (ppm) = 1.07].

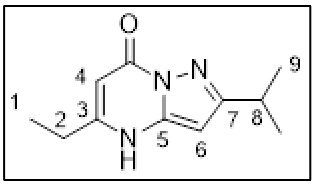


**5-Ethyl-2-isopropylpyrazolo[1,5-a]pyrimidin-7(4H)-one (19).** 3-Isopropyl-1H-pyrazol-5-amine (0.225 g, 1.8 mmol, 1 equiv), 5-ethyl-2-isopropylpyrazolo[1,5-a]pyrimidin-7(4H)-one (0.571 g, 3.96 mmol, 2.2 equiv), AcOH (85.8 µL, 0.75 mmol, 0.56 equiv) and MeOH (6 mL) were subjected to MW irradiation (100 W, 150 $^{o}$C) for 2 h. The MeOH was removed under reduced pressure to leave a residue and the residue was purified via precipitation with MeOH (1 mL) to give the title compound. Yield 0.118 g (32%); 0.24 (EtOAc); $^{1}$H NMR (500 MHz, DMSO) $\delta$ 12.07 (s, 1H, N<u>*H*</u>), 5.93 (s, 1H, H6), 5.51 (s, 1H, H4), 2.94 (dd, *J* = 13.9, 6.9 Hz, 1H, H8), 2.54 (q, *J* = 7.6 Hz, 2H, H2), 1.22 (d, *J* = 6.9 Hz, 6H, H9), 1.20 (t, *J* = 7.6 Hz, 3H, H1); $^{13}$C NMR (125 MHz, DMSO) $\delta$ 161.6 (C7), 156.5 (<u>*C*</u>O), 154.7 (C3), 142.0 (C5), 93.4 (C4), 85.4 (C6), 28.0 (C8), 25.6

(C2), 22.3 (C9), 12.6 (C1); IR (ATR) 3174, 3080, 2964, 2334, 2221, 1575, 1433, 1289 $cm^{-1}$; HR-MS calcd for $C_{12}H_{17}N_3O$ m/z: $[M+Na]^+$, 228.1215, found $[M+Na]^+$ = 228.1106 [Diff (ppm) = 0.73].

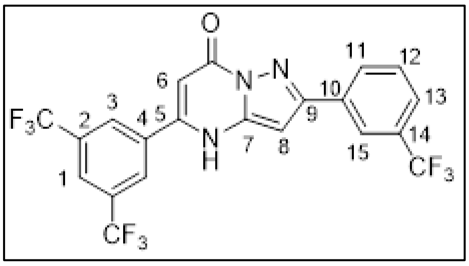


**5-(3,5-Bis(trifluoromethyl)phenyl)-2-(3-(trifluoromethyl)phenyl)pyrazolo[1,5-a]pyrimidin-7(4H)-one (20).** 3-(3-(Trifluoromethyl)phenyl)-1H-pyrazol-5-amine (0.2044 g, 0.9 mmol, 1 equiv), ethyl 3-(3,5-bis(trifluoromethyl)phenyl)-3-oxopropanoate (0.650 g, 1.98 mmol, 2.2 equiv), AcOH (42.9 µL, 0.75 mmol, 0.56 equiv) and MeOH (3 mL) were subjected to MW irradiation (100 W, 150 $^oC$) for 2 h. The resulting product precipitated out in MeOH (3 mL) to give the title compound. Yield 0.252 g, 57%; $R_f$ 0.82 (Petroleum ether/ EtOAc 1:1 v/v); $^1H$ NMR (500 MHz, DMSO) $\delta$ 12.93 (s, 1H, N*H*), 8.54 (s, 2H, H3), 8.34 (s, 1H, H1), 8.32 (d, *J* = 7.7 Hz, 2H, H11 & H15), 7.78 (d, *J* = 7.4 Hz, 1H, H13), 7.73 (t, *J* = 7.6 Hz, 1H, H12), 6.86 (s, 1H, H8), 6.40 (s, 1H, H6); $^{13}C$ NMR (125 MHz, DMSO) $\delta$ 155.9 (*C*O), 152.0 (C9), 147.0 (C5), 143.1 (C7), 134.8 (C4), 133.3 (C10), 130.8 (q, *J* = 33.3 Hz, C2), 130.3 (C11), 130.0 (C12), 129.9 (q, *J* = 37.5 Hz, C14), 128.6 (C3), 125.9 (q, *J* = 137.5 Hz, $\underline{C}F_3$), 125.5 (C13), 124.4 (C1), 123.6 (q, *J* = 137.5 Hz, $2\underline{C}F_3$), 122.3 (C15), 96.1 (C6), 87.2 (C8); $^{19}F$ NMR (471 MHz, DMSO) $\delta$ -61.21 ($C\underline{F}_3$); IR (ATR) 3381, 1682, 1612, 1370, 1283, 1187, 1118, 696.9 $cm^{-1}$; HR-MS calcd for $C_{21}H_{10}F_9N_3O$ m/z: $[M+Na]^+$, 514.0676 , found $[M+Na]^+$ = 514.0680 [Diff (ppm) =-0.78].

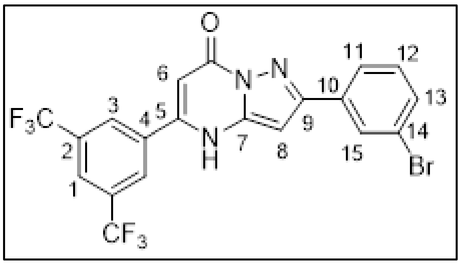


**5-(3,5-Bis(trifluoromethyl)phenyl)-2-(3-bromophenyl)pyrazolo[1,5-α]pyrimidin-7(4¬H)-one (21).** 3-(3-Bromophenyl)-1H-pyrazol-5-amine (0.22g, 0.9 mmol, 1 equiv), ethyl 3-(3,5-bis(trifluoromethyl)phenyl)-3-oxopropanoate (0.650 g, 1.98 mmol, 2.2 equiv), AcOH (42.9 µL, 0.75 mmol, 0.56 equiv) and MeOH (5 mL) were subjected to MW irradiation (100 W, 150 $^oC$) for 2 h. The resulting mixture was purified using hot filtration with MeOH (3 mL), to give the title compound. Yield 0.15 g, 33%; Rf: 0.61 (EtOAc); $^1H$ NMR (500 MHz, DMSO) $\delta$ 12.93 (s, 1H, N*H*), 8.54 (s, 2H,

H3), 8.36 (s, 1H, H1), 8.20 (s, 1H, H15), 8.04 (d, *J* = 7.8 Hz, 1H, H11), 7.63 (d, *J* = 8.0 Hz, 1H, H13), 7.46 (t, *J* = 7.9 Hz, 1H, H12), 6.80 (s, 1H, H8), 6.41 (s, 1H, H6); $^{13}$C NMR (125 MHz, DMSO) $\delta$ 156.0 (*C*O), 152.1 (C9), 147.0 (C5), 143.1 (C7), 134.8 (C4), 134.6 (C14), 131.8 (C13), 131.0 (C12), 130.9 (q, *J* = 33.3 Hz, C2), 128.7 (C15), 128.6 (C3), 125.3 (C11), 124.5 (C1), 123.1 (q, *J* = 272.5 Hz, *C*$F_3$), 122.2 (C10), 96.0 (C6), 87.2 (C8); $^{19}$F NMR (471 MHz, DMSO) $\delta$ -61.15 (C*F*$_3$); IR (ATR) 3449, 1665, 1606, 1459, 1369, 682.5 cm$^{-1}$; HR-MS calcd $C_{20}H_{10}BrF_6N_3O$ m/z: [M + Na]$^+$, 523.9911, found [M + Na]$^+$ = 523.9807 [Diff (ppm) = 0.55].

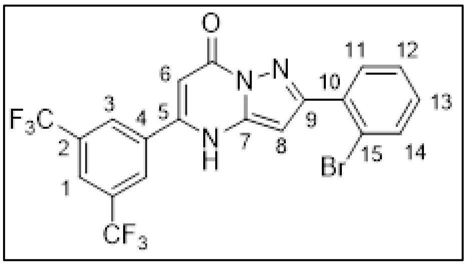


**5-(3,5-Bis(trifluoromethyl)phenyl)-2-(2-bromophenyl)pyrazolo[1,5-a]pyrimidin-7(4H)-one (22).** 3-(2-Bromophenyl)-1H-pyrazol-5-amine (0.220 g, 0.9 mmol, 1 equiv), ethyl 3-(3,5-bis(trifluoromethyl)phenyl)-3-oxopropanoate, (0.650 g, 1.98 mmol, 2.2 equiv), AcOH (42.9 µL, 0.75 mmol, 0.56 equiv) and MeOH (3 mL) were subjected to MW irradiation (100 W, 150 $^{o}$C) for 2 h. The MeOH was removed under reduced pressure to leave a residue, and the residue was purified via column chromatography (70:30 petroleum ether/EtOAc to 100 EtOAc) yielding the the title compound. Yield 0.306 g 68%; $R_f$ 0.65 (EtOAc); $^{1}$H NMR (500 MHz, DMSO) $\delta$ 8.67 (s, 2H, H3), 8.12 (s, 1H, H1), 7.81 (d, *J* = 5.6 Hz, 1H, H14), 7.73 (d, *J* = 8.0 Hz, 1H, H11), 7.44 (t, *J* = 7.1 Hz, 1H, H13), 7.31 (t, *J* = 7.5 Hz, 1H, H12), 6.60 (s, 1H, H8), 6.30 (s, 1H, H6); $^{13}$C NMR (125 MHz, DMSO) $\delta$ 158.4 (*C*O), 152.7 (C5), 151.67 (C9) 148.5 (C7), 142.0 (C4), 135.4 (C10), 133.2 (C11), 131.8 (C14), 130.5 (q, *J* = 25 Hz, C2), 129.6 (C12), 127.5 (C13), 126.9 (C3), 123.4 (q, *J* = 275 Hz (C*F*$_3$), 122.4 (C15), 121.9 (C1), 94.4 (C8), 89.2 (C6); $^{19}$F NMR (471 MHz, DMSO) $\delta$ -61.21 (C*F*$_3$); IR (ATR) 2922, 1605, 1540, 1276, 1127, 680 cm$^{-1}$ ; HR-MS calcd for $C_{20}H_{10}BrF_6N_3ONa$ m/z: [M + Na]$^+$, 523.9911; found [M + Na]$^+$ = 523.9803 [Diff (ppm) = -0.29].

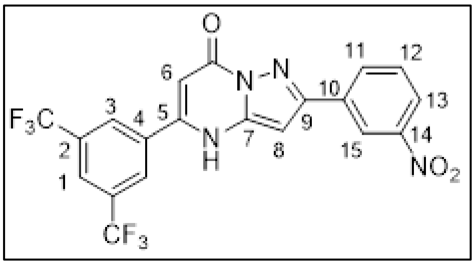


**5-(3,5-Bis(trifluoromethyl)phenyl)-2-(3-nitrophenyl)pyrazolo[1,5-a]pyrimidin-7(4H)-one (23).** 3-(3-Nitrophenyl)-1H-pyrazol-5-amine (0.1837 g, 0.9 mmol, 1 equiv), ethyl 3-(3,5-bis(trifluoromethyl)phenyl)-3-oxopropanoate (0.650 g, 1.97 mmol, 2.2 equiv), AcOH (42.9 µL, 0.75 mmol, 0.56 equiv) and MeOH (3 mL) were subjected to MW irradiation (100 W, 150 $^{o}$C) for 2 h. The resulting product precipitated out in MeOH (3 mL) to give the title compound. This solid underwent hot recrystallization using EtOH (3 mL) to give a white solid. Yield 0.143 g, 34%; $R_f$ 0.68 (Petroleum ether/ EtOAc 1:1 v/v); $^1$H NMR (500 MHz, DMSO) $\delta$ 12.95 (s, 1H, N*H*), 8.74 (s, 1H, H15), 8.52 (s, 2H, H3), 8.42 (d, $J$ = 7.4 Hz, 1H, H13 ), 8.34 (s, 1H, H1), 8.23 (d, $J$ = 7.8 Hz, 1H, H11 ), 7.76 (t, $J$ = 7.8 Hz, 1H, H12), 6.85 (s, 1H, H8), 6.40 (s, 1H, H6); $^{13}$C NMR (125 MHz, DMSO) $\delta$ 155.9 (*C*O), 151.4 (C9), 148.3 (C14), 147.0 (C5), 143.2 (C7), 134.7 (C4), 133.9 (C10), 132.7 (C13), 130.9 (q, $J$ = 37.5 Hz, C2), 130.4 (C12), 128.6 (C3),124.5 (C1), 123.5 (C11), 123.1 (q, $J$ = 273.0 Hz, *C*F$_3$), 120.3 (C15), 96.1 (C6), 87.5 (C8); $^{19}$F NMR (471 MHz, DMSO) $\delta$ -61.16 (C*F*$_3$); IR (ATR) 3141, 2883, 1687, 1615, 1518, 1350, 1274, 1139 cm$^{-1}$; HR-MS calcd for $C_{20}H_{10}F_6N_4O_3$ m/z: [M+H]$^+$, 469.0657, found [M+Na]$^+$ = 469.0730 [Diff (ppm) =0.13].

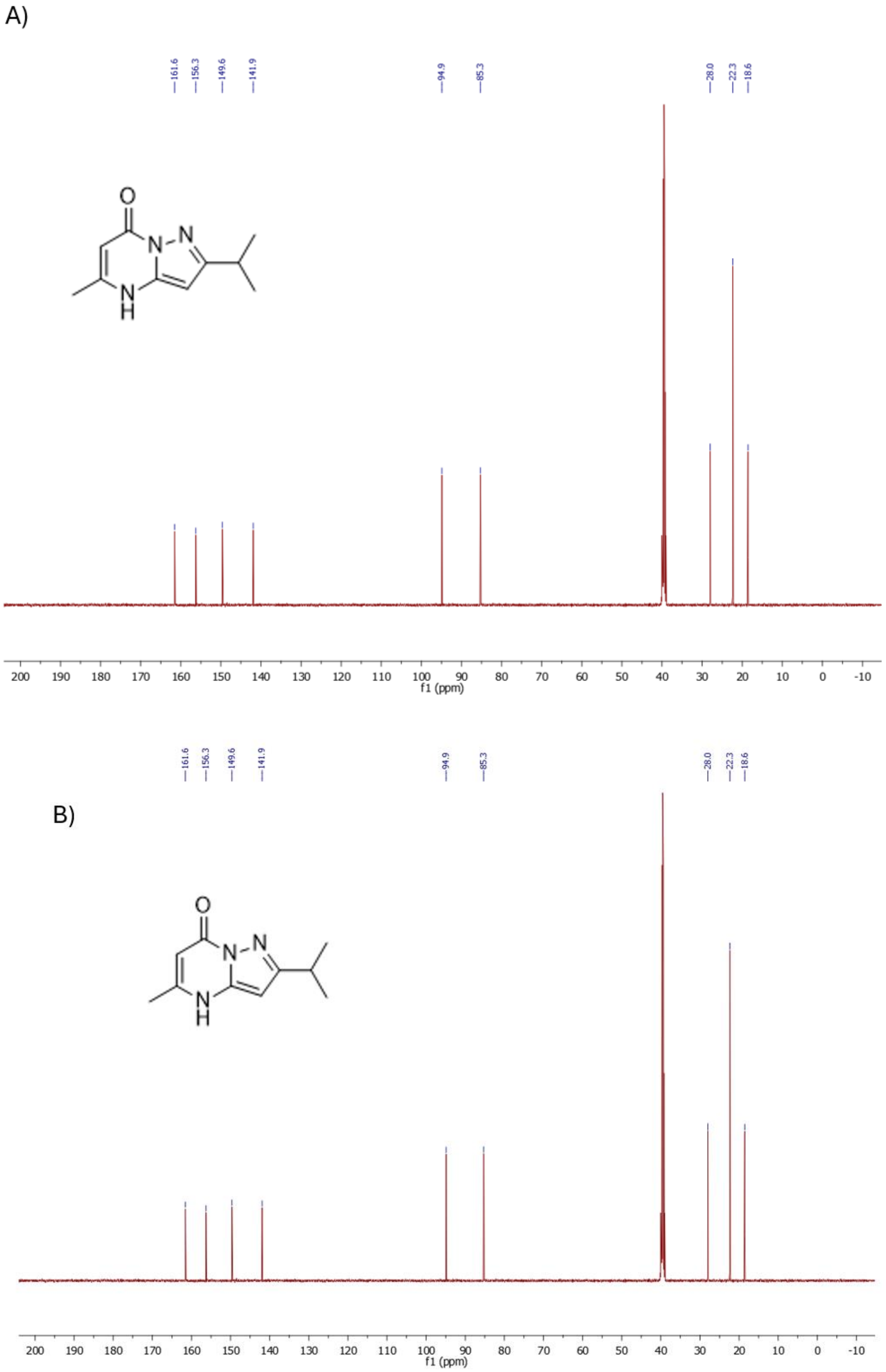


**Figure 1:** $^{1}H$ (500 MHz, DMSO-$d_6$) (A) and $^{13}C$ (125 MHz, DMSO-$d_6$) (B) NMR spectra of 2-isopropyl-5-methylpyrazolo[1,5-a]pyrimidin-7(4H)-one (**1**) in DMSO.

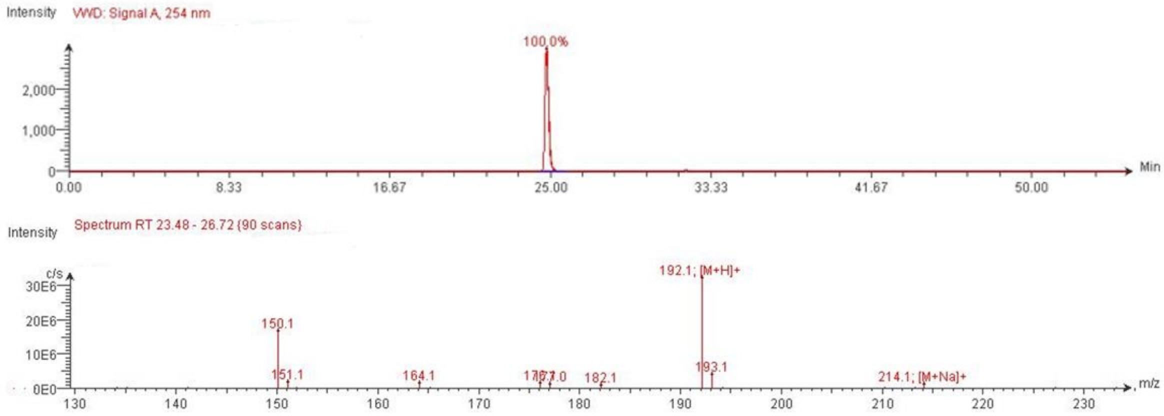


**Figure 2:** LCMS of 2-isopropyl-5-methylpyrazolo[1,5-a]pyrimidin-7(4H)-one (**1**), representing its $(M+H)^+$ value 192.1.

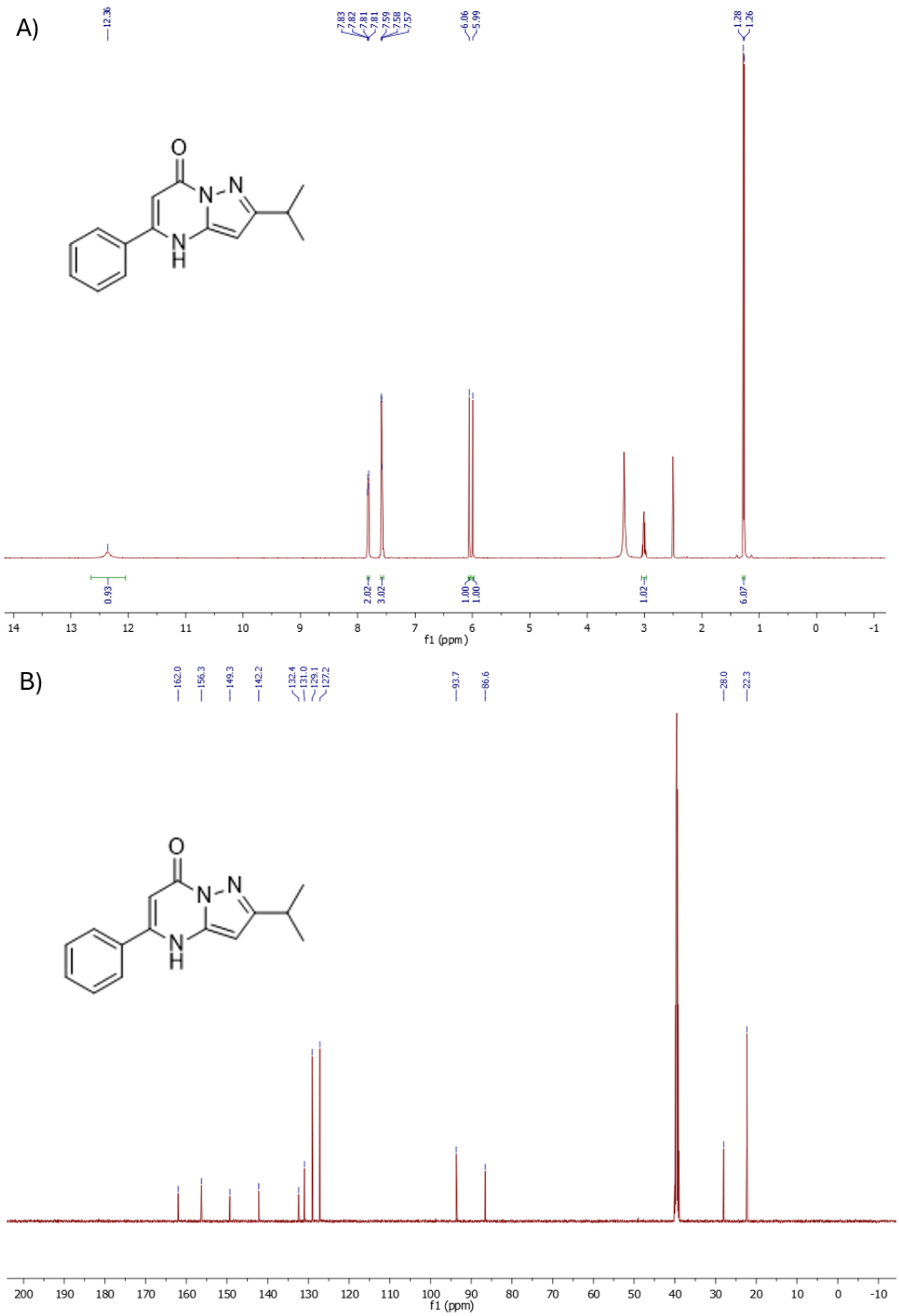


**Figure 3:** $^{1}H$ (500 MHz, DMSO-$d_6$) (A) and $^{13}C$ (125 MHz, DMSO-$d_6$) (B) NMR spectra of 2-isopropyl-5-phenylpyrazolo[1,5-a]pyrimidin-7(4H)-one (**2**).

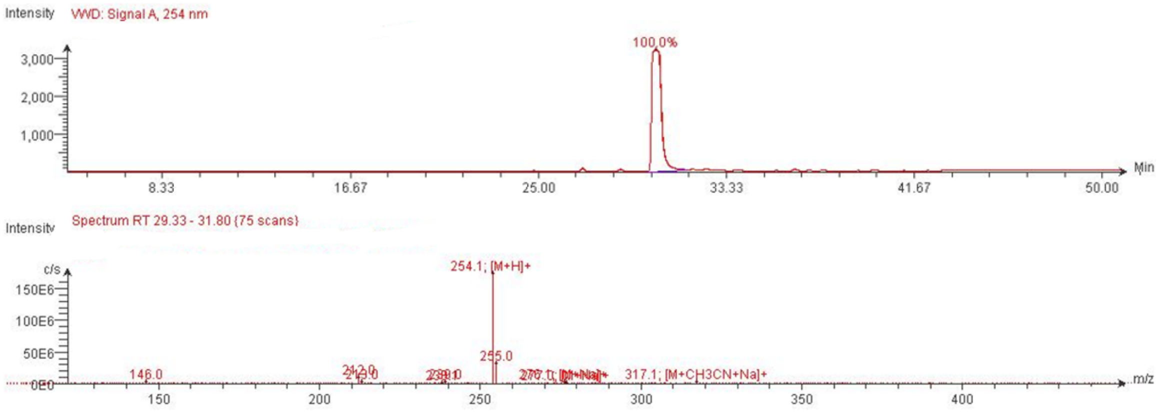


**Figure 4:** LCMS of 2-isopropyl-5-phenylpyrazolo[1,5-a]pyrimidin-7(4H)-one (**2**), representing its $(M+H)^+$ value 254.1.

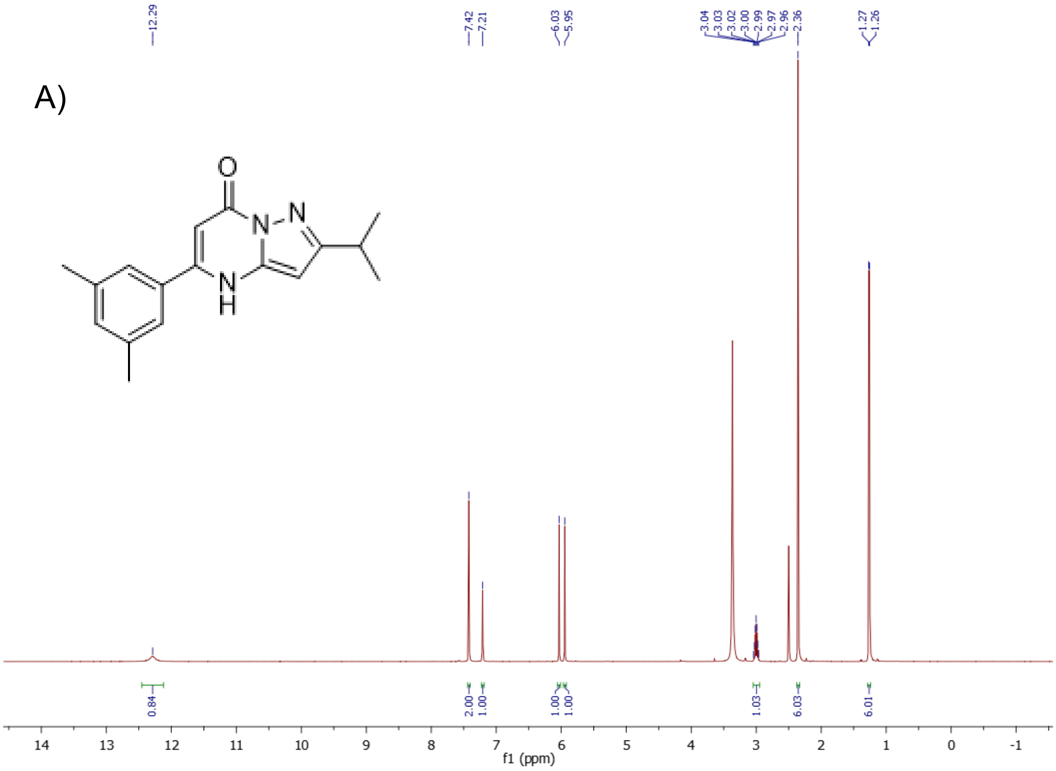


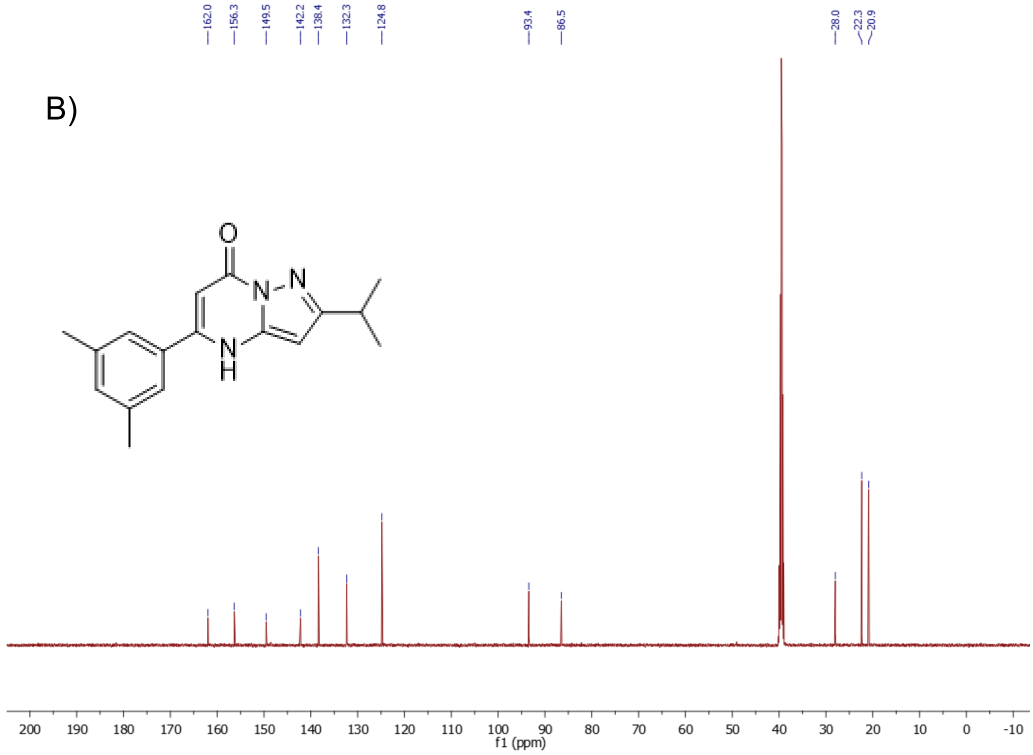


**Figure 5:** $^{1}$H (500 MHz, DMSO-$d_6$) (A) and $^{13}$C (125 MHz, DMSO-$d_6$) (B) NMR spectra of 5-(3,5-dimethylphenyl)-2-isopropylpyrazolo[1,5-a]pyrimidin-7(4H)-one (**3**).

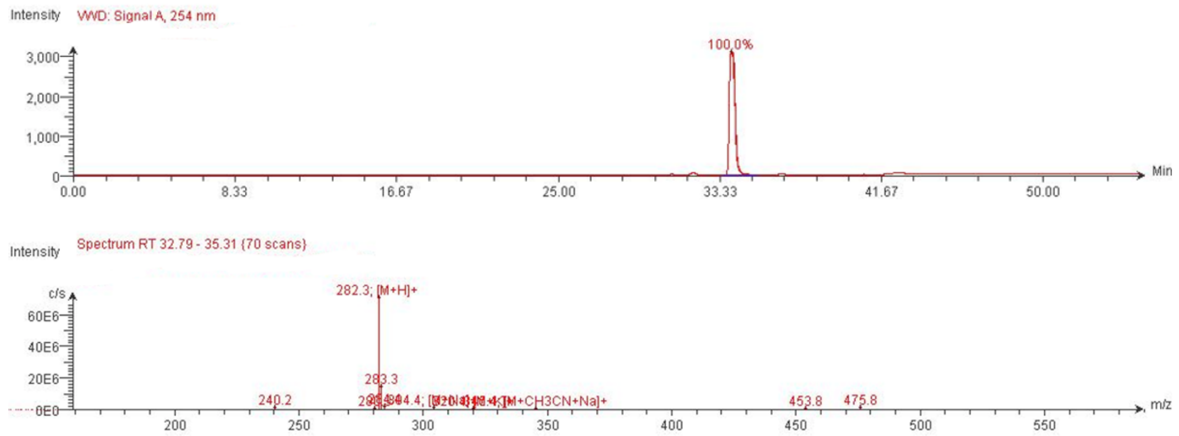


**Figure 6:** LCMS of 5-(3,5-dimethylphenyl)-2-isopropylpyrazolo[1,5-a]pyrimidin-7(4H)-one (**3**), representing its $(M+H)^+$ value 282.3.

A)

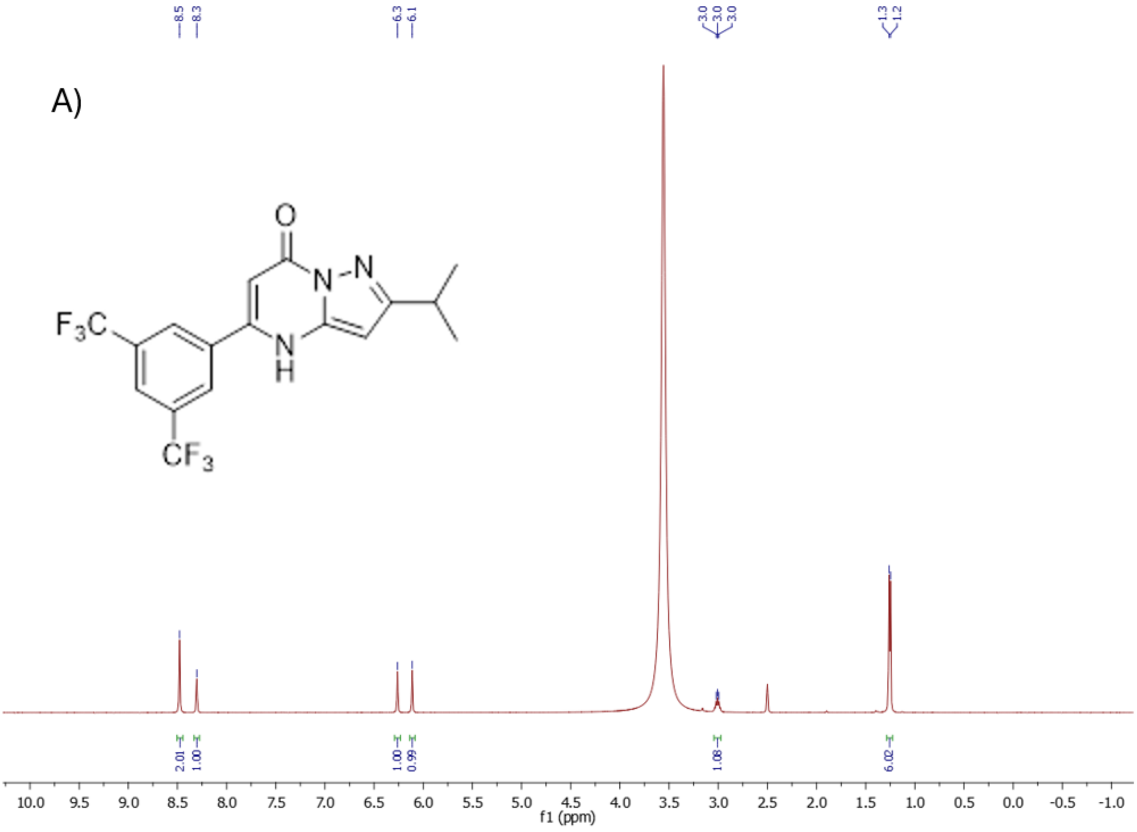
8.5
8.3
6.3
6.1
3.0
3.0
3.0
1.3
1.2
O
N
N
$F_3C$
N
H
$CF_3$
2.01
1.00
1.00
0.99
1.08
6.02
10.0 9.5 9.0 8.5 8.0 7.5 7.0 6.5 6.0 5.5 5.0 4.5 4.0 3.5 3.0 2.5 2.0 1.5 1.0 0.5 0.0 -0.5 -1.0
f1 (ppm)

B)

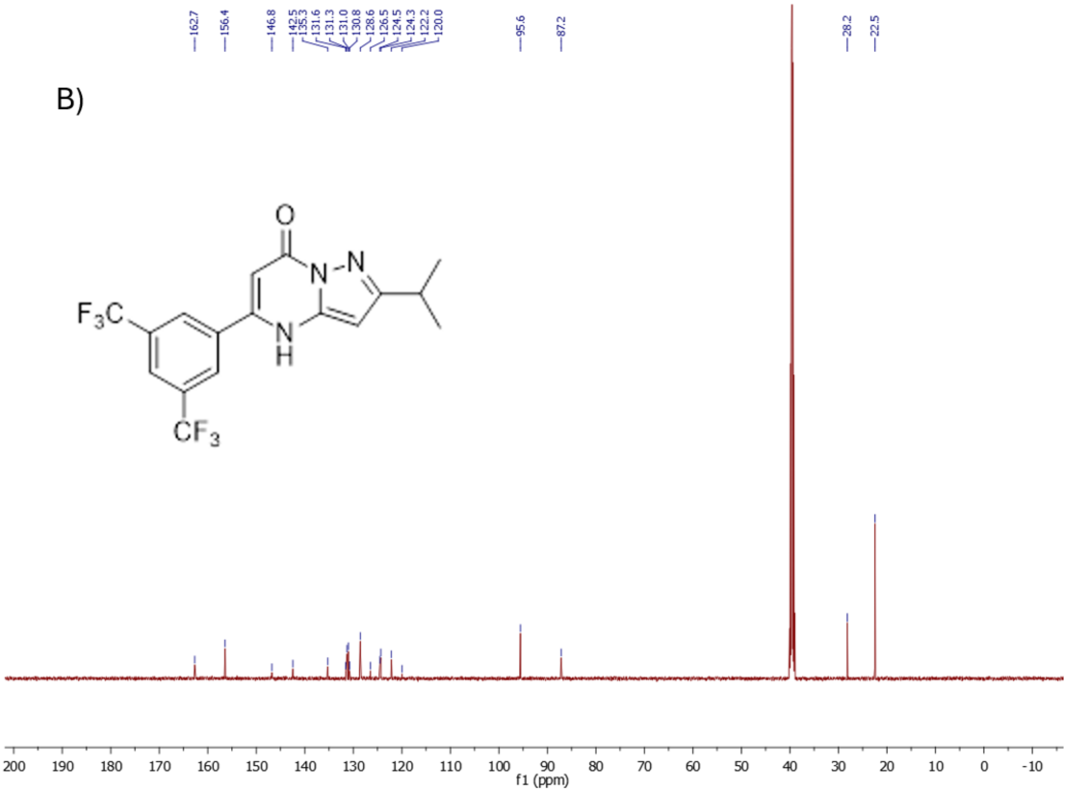
162.7
156.4
146.8
142.5
135.3
131.6
131.3
131.0
130.8
128.6
126.5
124.5
124.3
122.2
120.0
95.6
87.2
28.2
22.5
O
N
N
$F_3C$
N
H
$CF_3$
200 190 180 170 160 150 140 130 120 110 100 90 80 70 60 50 40 30 20 10 0 -10
f1 (ppm)

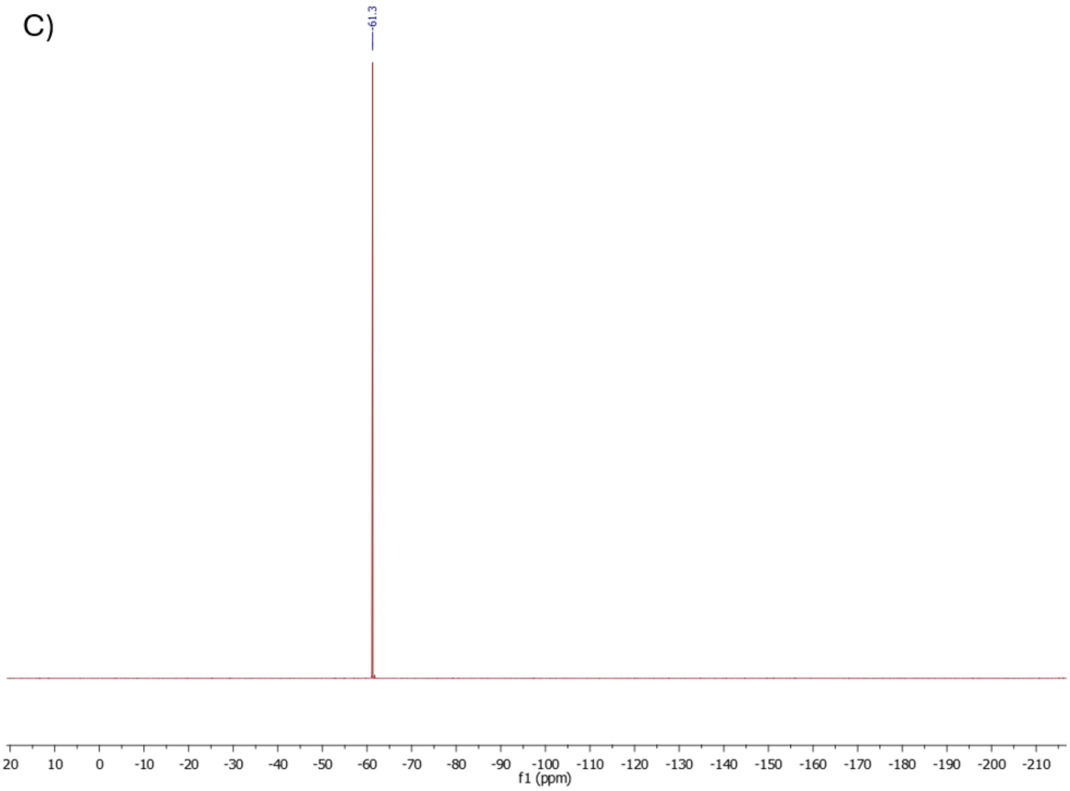


**Figure 7:** $^{1}H$ (500 MHz, DMSO-$d_6$) (A) and $^{13}C$ (125 MHz, DMSO-$d_6$) (B) $^{19}F$ (471 MHz, DMSO-$d_6$) (C) NMR spectra of 5-(3,5-bis(trifluoromethyl)phenyl)-2-isopropylpyrazolo[1,5-a]pyrimidin-7(4H)-one (**4**).

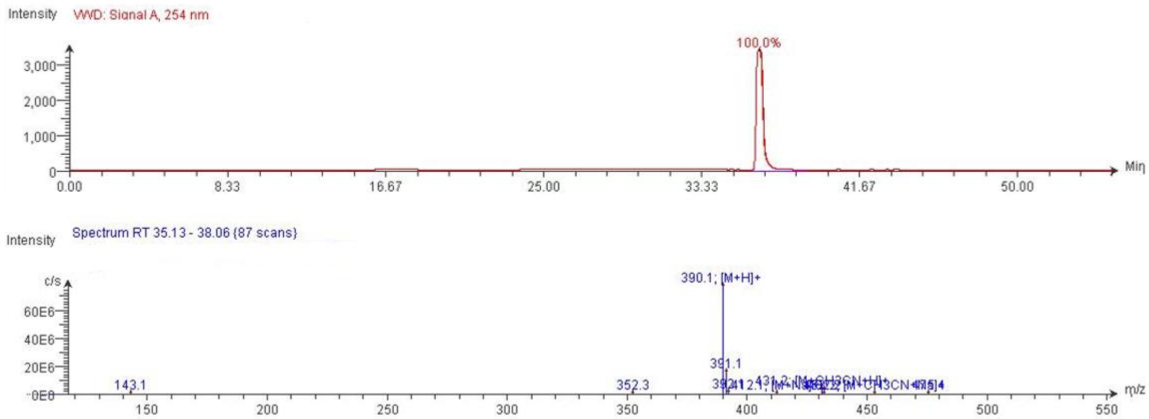


**Figure 8:** LCMS of 5-(3,5-bis(trifluoromethyl)phenyl)-2-isopropylpyrazolo[1,5-a]pyrimidin-7(4H)-one (**4**), representing its $(M+H)^{+}$ value 390.1.

A)

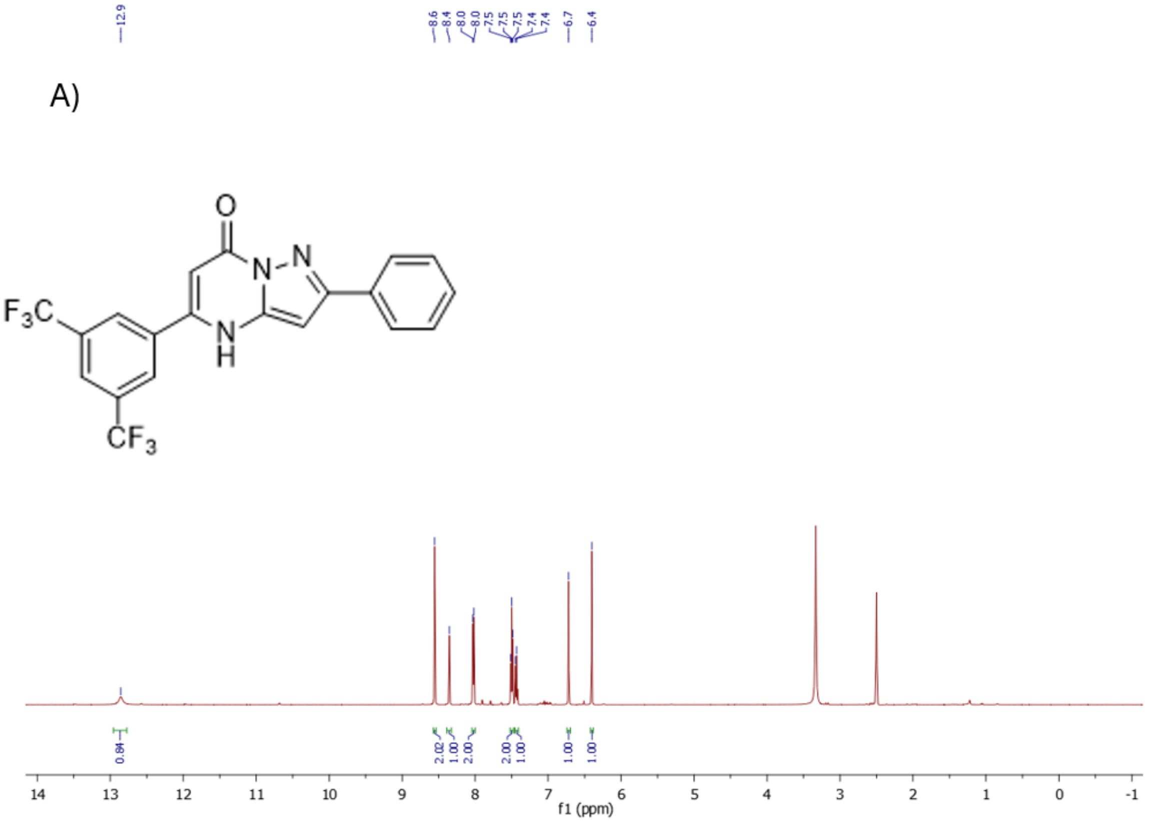

12.9
8.6
8.4
8.0
8.0
7.5
7.5
7.5
7.4
7.4
6.7
6.4
O
N
N
F3C
N
H
CF3
0.84
2.02
1.00
2.00
2.00
1.00
1.00
1.00
14
13
12
11
10
9
8
7
6
5
4
3
2
1
0
-1
f1 (ppm)


B)

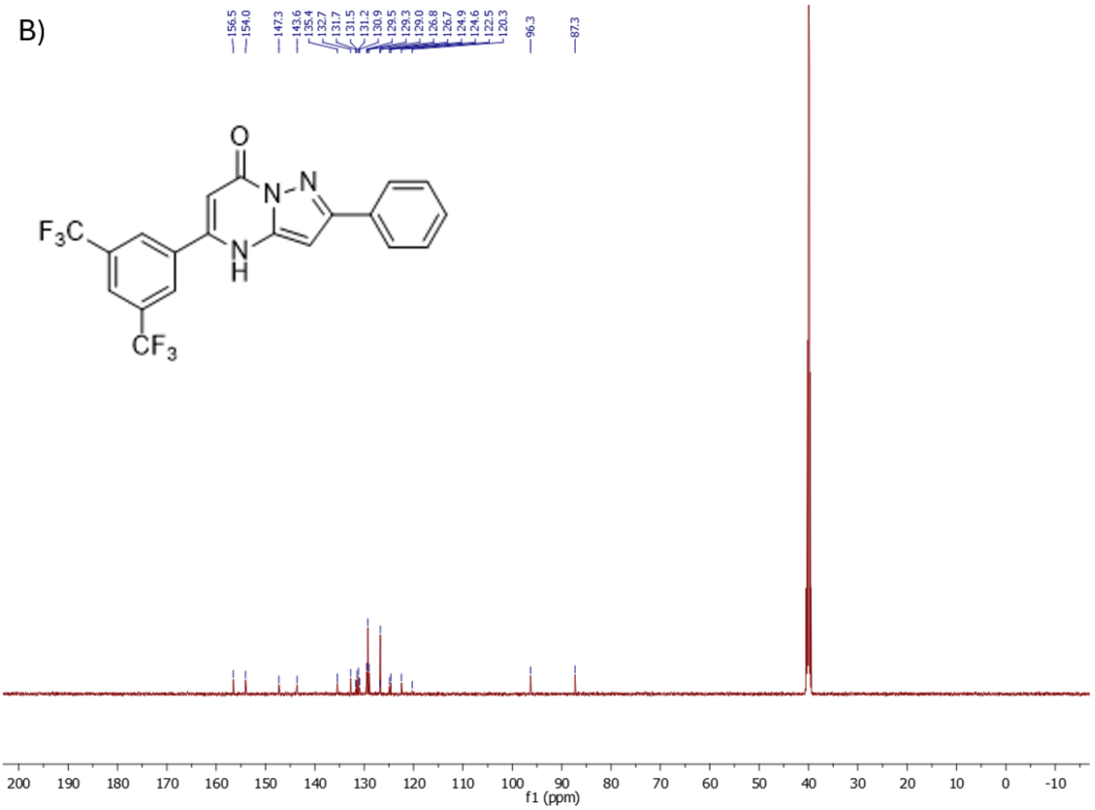

156.5
154.0
147.3
143.6
135.4
132.7
131.7
131.5
131.2
130.9
129.5
129.3
129.0
126.8
126.7
124.9
124.6
122.5
120.3
96.3
87.3
O
N
N
F3C
N
H
CF3
200
190
180
170
160
150
140
130
120
110
100
90
80
70
60
50
40
30
20
10
0
-10
f1 (ppm)

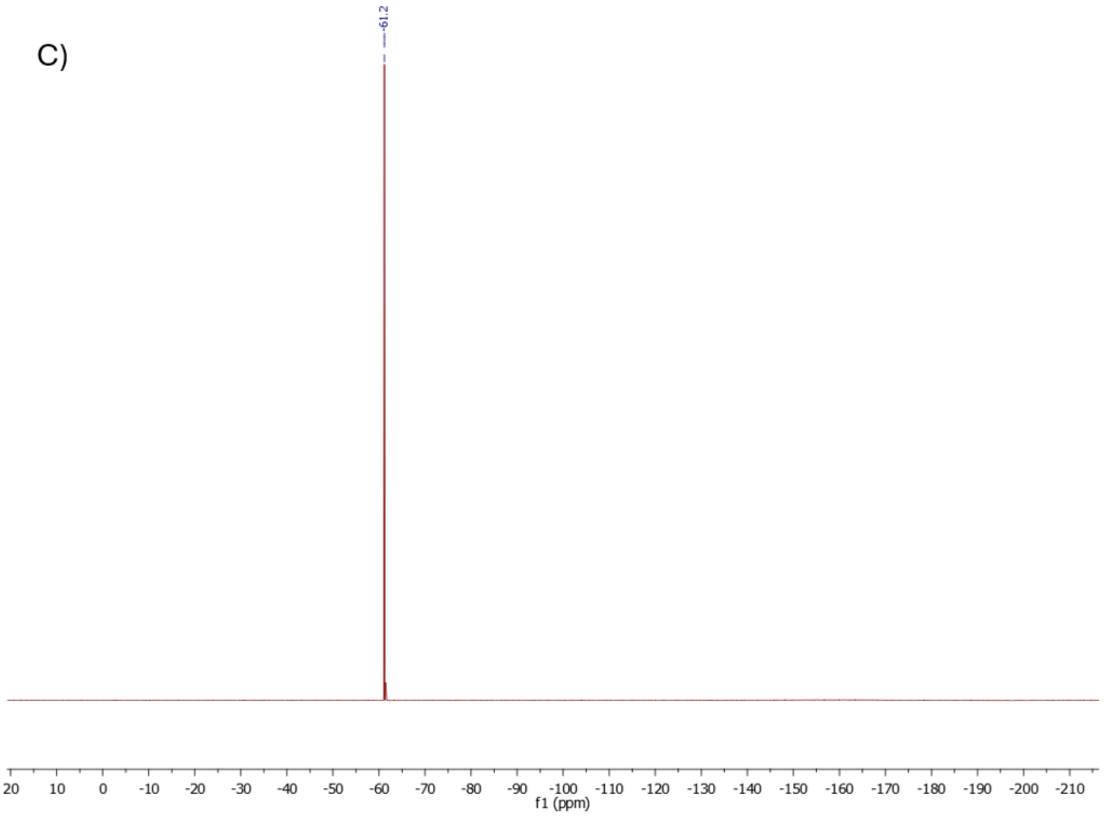


**Figure 9:** $^{1}H$ (500 MHz, DMSO-$d_6$) (A) and $^{13}C$ (125 MHz, DMSO-$d_6$) (B) $^{19}F$ (471 MHz, DMSO-$d_6$) (C) NMR spectra of 5-(3,5-bis(trifluoromethyl)phenyl)-2-phenylpyrazolo[1,5-a]pyrimidin-7(4H)-one (**5**).

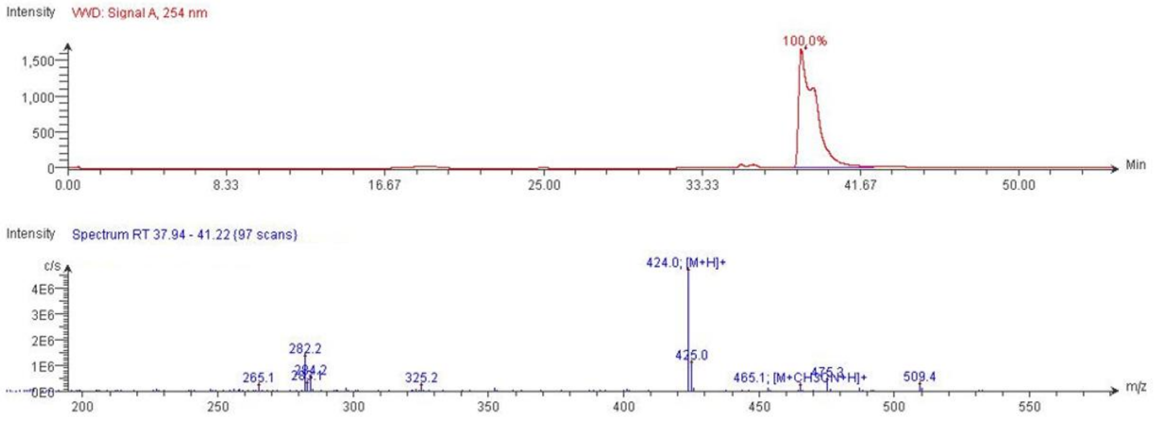


**Figure 10:** LCMS of 5-(3,5-bis(trifluoromethyl)phenyl)-2-phenylpyrazolo[1,5-a]pyrimidin-7(4H)-one (**5**), representing its $(M+H)^{+}$ value 424.0.

A)

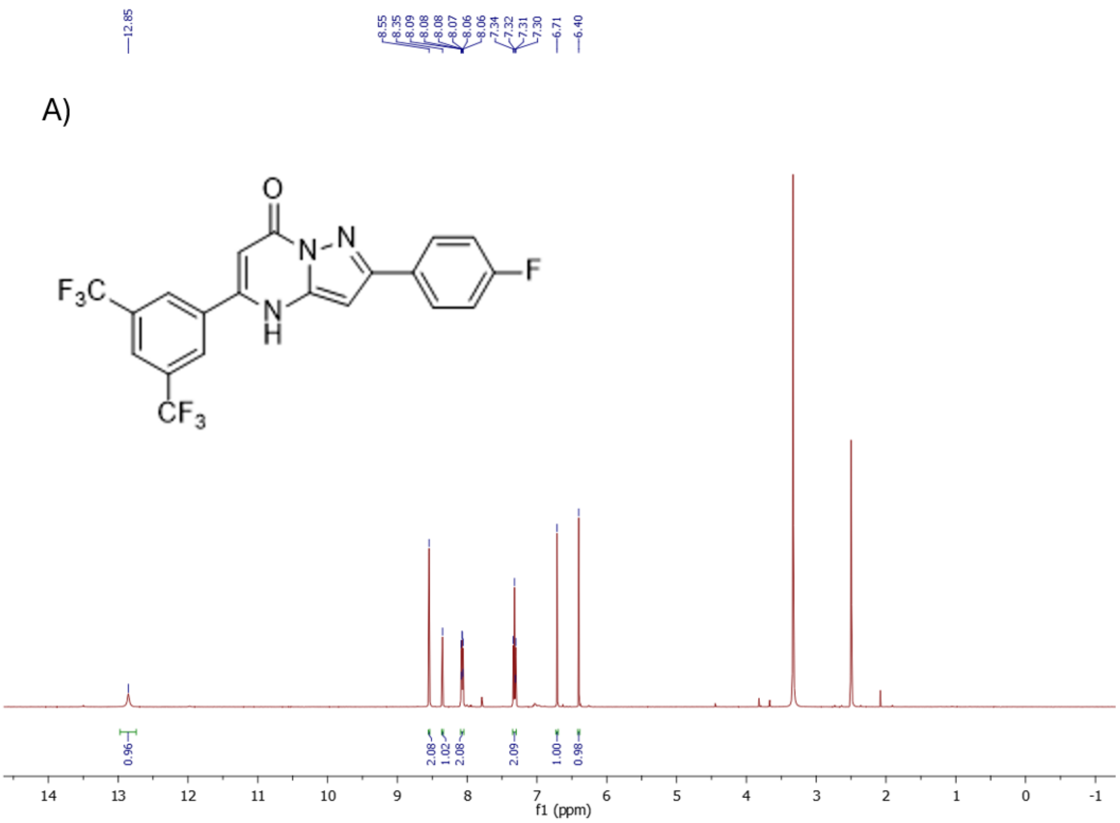

B)

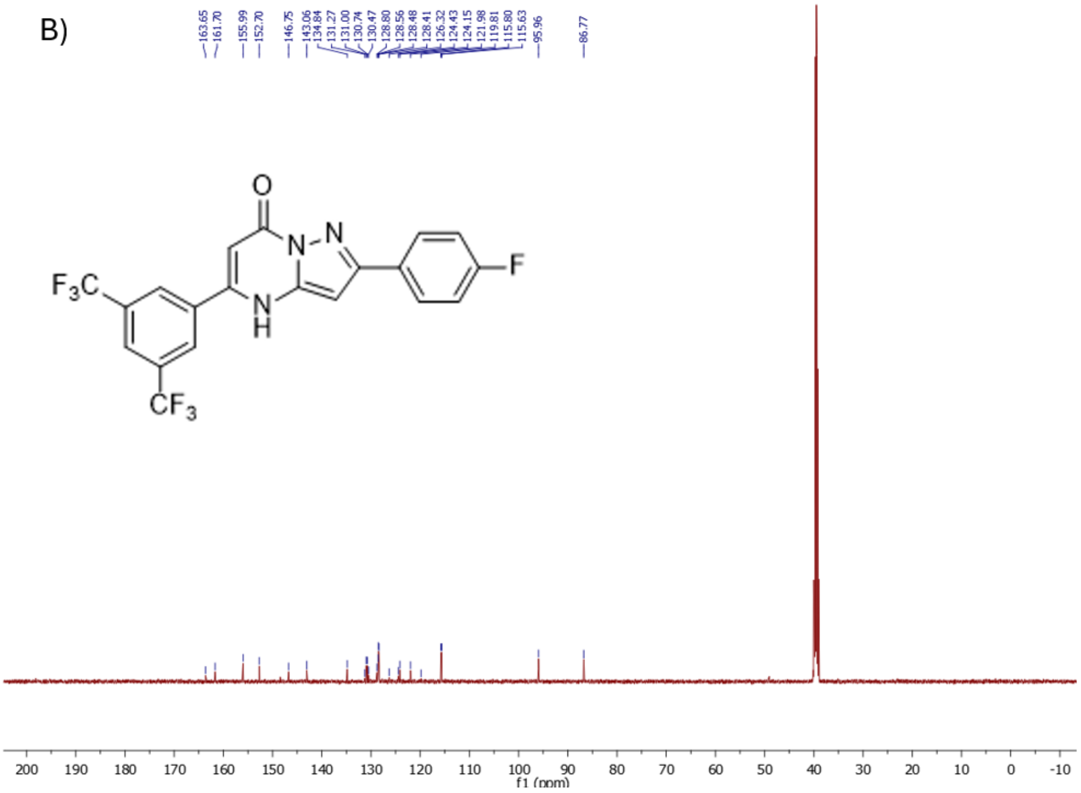

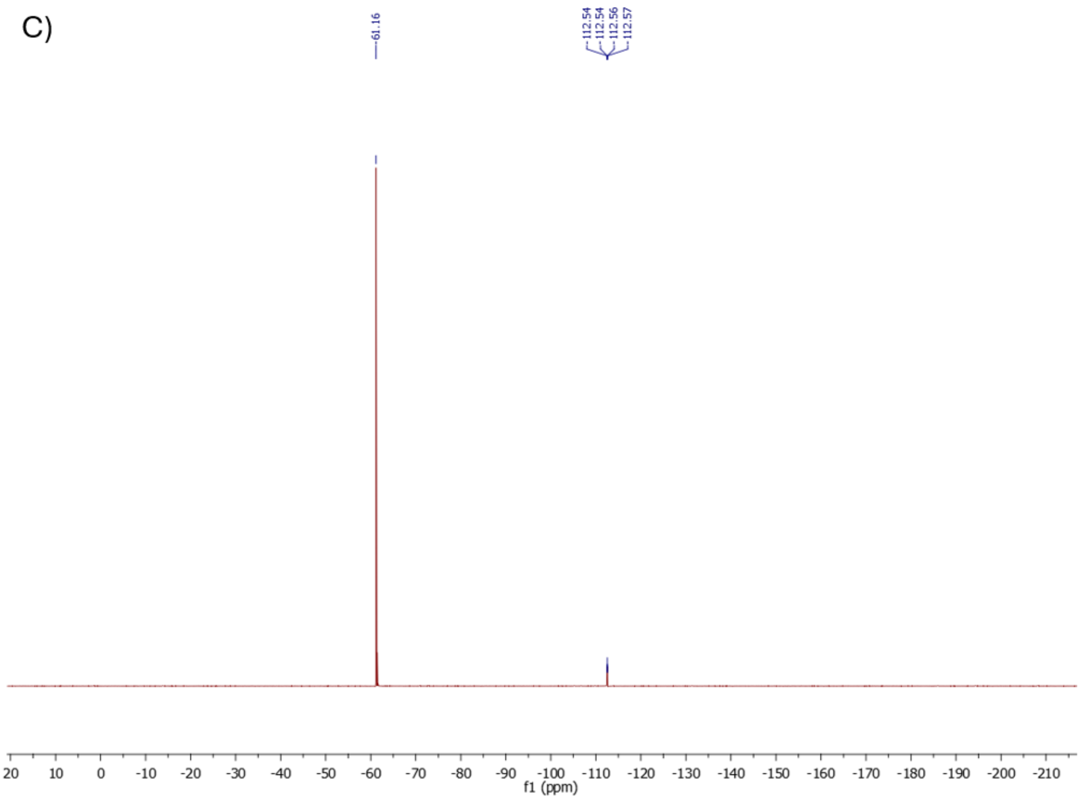


**Figure 11:** $^{1}H$ (500 MHz, DMSO-$d_6$) (A) and $^{13}C$ (125 MHz, DMSO-$d_6$) (B) $^{19}F$ (471 MHz, DMSO-$d_6$) (C) NMR spectra of 5-(3,5-bis(trifluoromethyl)phenyl)-2-(4-fluorophenyl)pyrazolo[1,5-a]pyrimidin-7(4H)-one (**6**).

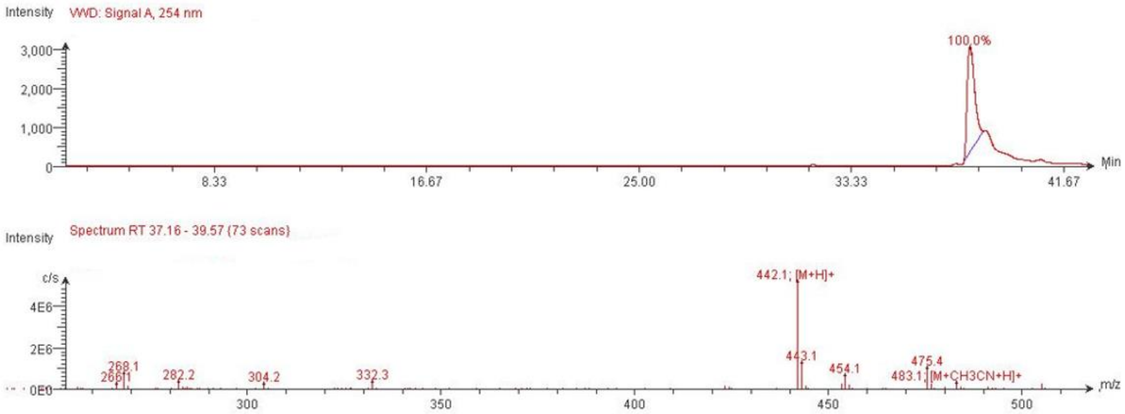


**Figure 12:** LCMS of 5-(3,5-bis(trifluoromethyl)phenyl)-2-(4-fluorophenyl)pyrazolo[1,5-a]pyrimidin-7(4H)-one (**6**), representing its $(M+H)^+$ value 442.1.

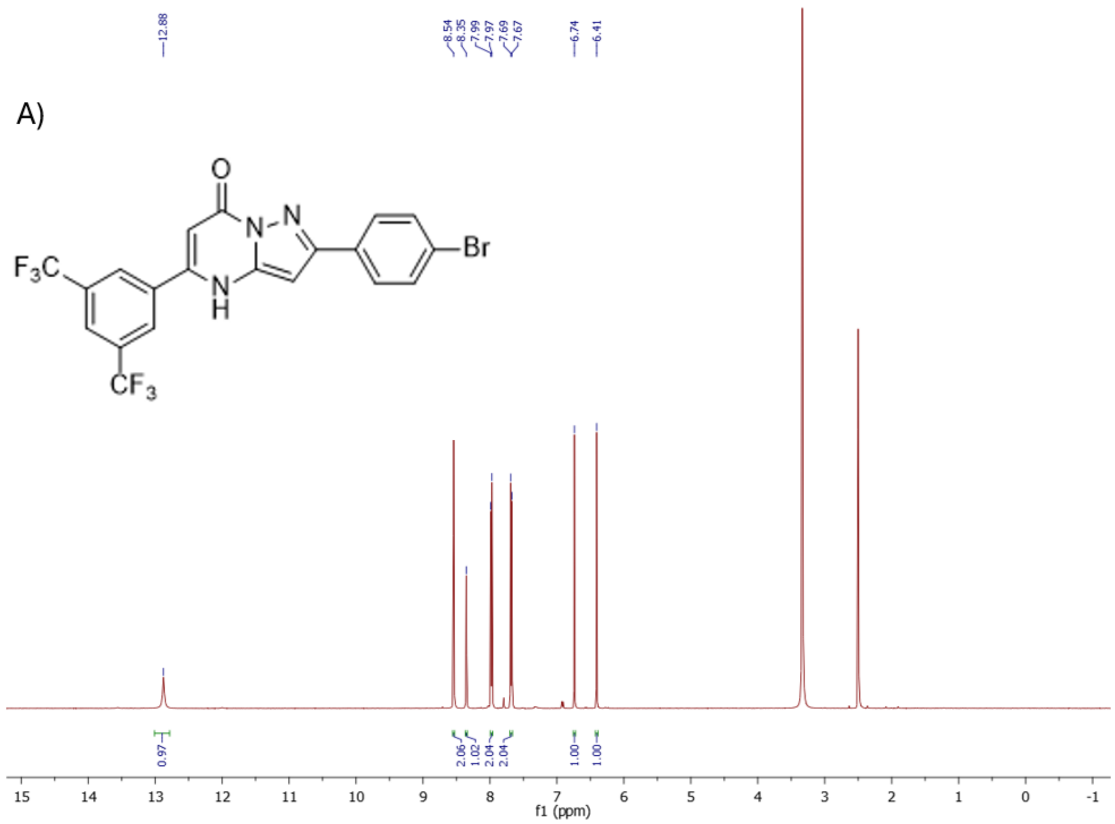
A)
12.88
8.54
8.35
7.99
7.97
7.69
7.67
6.74
6.41
F3C
CF3
O
N
N
H
Br
0.97
2.06
1.02
2.04
2.04
1.00
1.00
f1 (ppm)

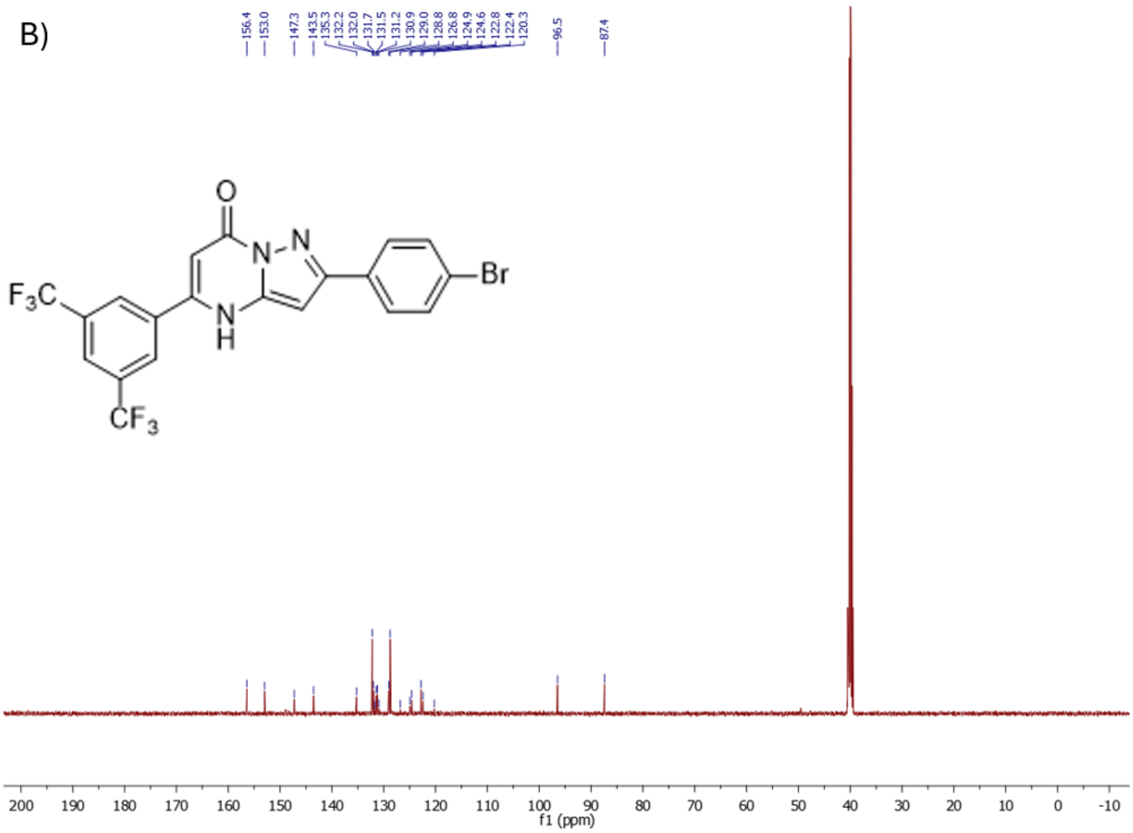
B)
156.4
153.0
147.3
143.5
135.3
132.2
132.0
131.7
131.5
131.2
130.9
129.0
128.8
126.8
124.9
124.6
122.8
122.4
120.3
96.5
87.4
F3C
CF3
O
N
N
H
Br
f1 (ppm)

C)

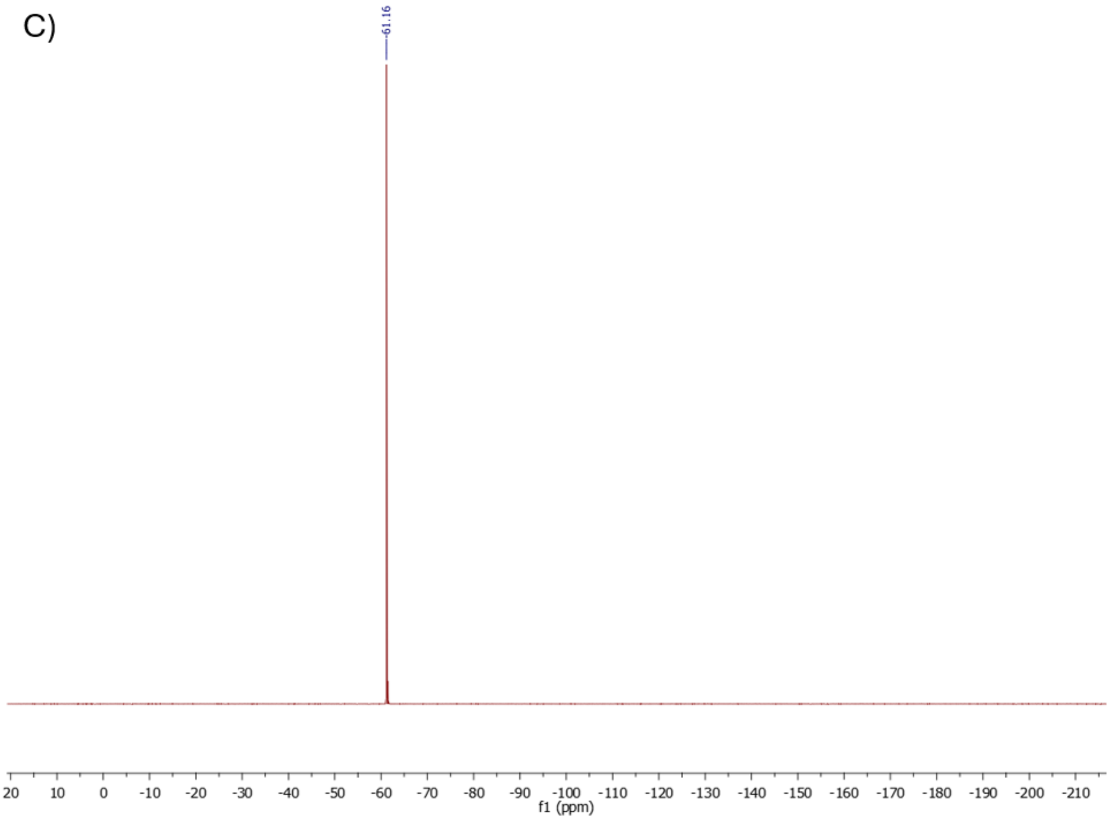


**Figure 13:** $^{1}H$ (500 MHz, DMSO-$d_6$) (A) and $^{13}C$ (125 MHz, DMSO-$d_6$) (B) $^{19}F$ (471 MHz, DMSO-$d_6$) (C) NMR spectra of 5-(3,5-bis(trifluoromethyl)phenyl)-2-(4-bromophenyl)pyrazolo[1,5-a]pyrimidin-7(4H)-one (**7**).

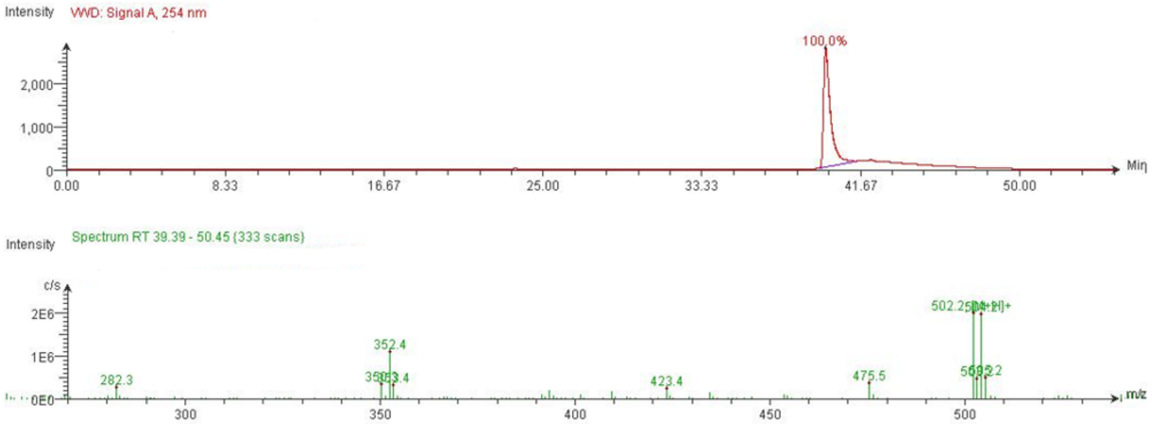


**Figure 14:** LCMS of 5-(3,5-bis(trifluoromethyl)phenyl)-2-(4-bromophenyl)pyrazolo[1,5-a]pyrimidin-7(4H)-one (**7**), representing its $(M+H)^{+}$ value 502.2.

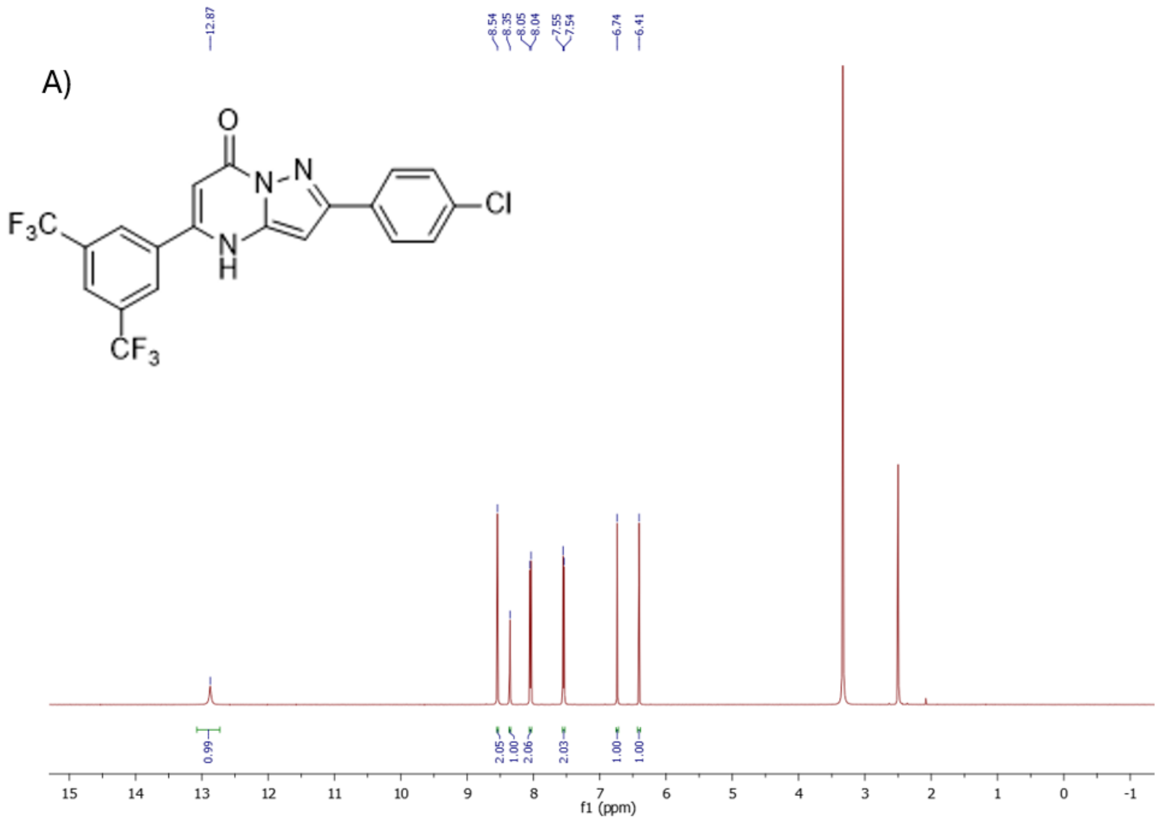
A)
12.87
8.54
8.35
8.05
8.04
7.55
7.54
6.74
6.41
0.99
2.05
1.00
2.06
2.03
1.00
1.00
f1 (ppm)
O
N
N
H
Cl
F3C
CF3

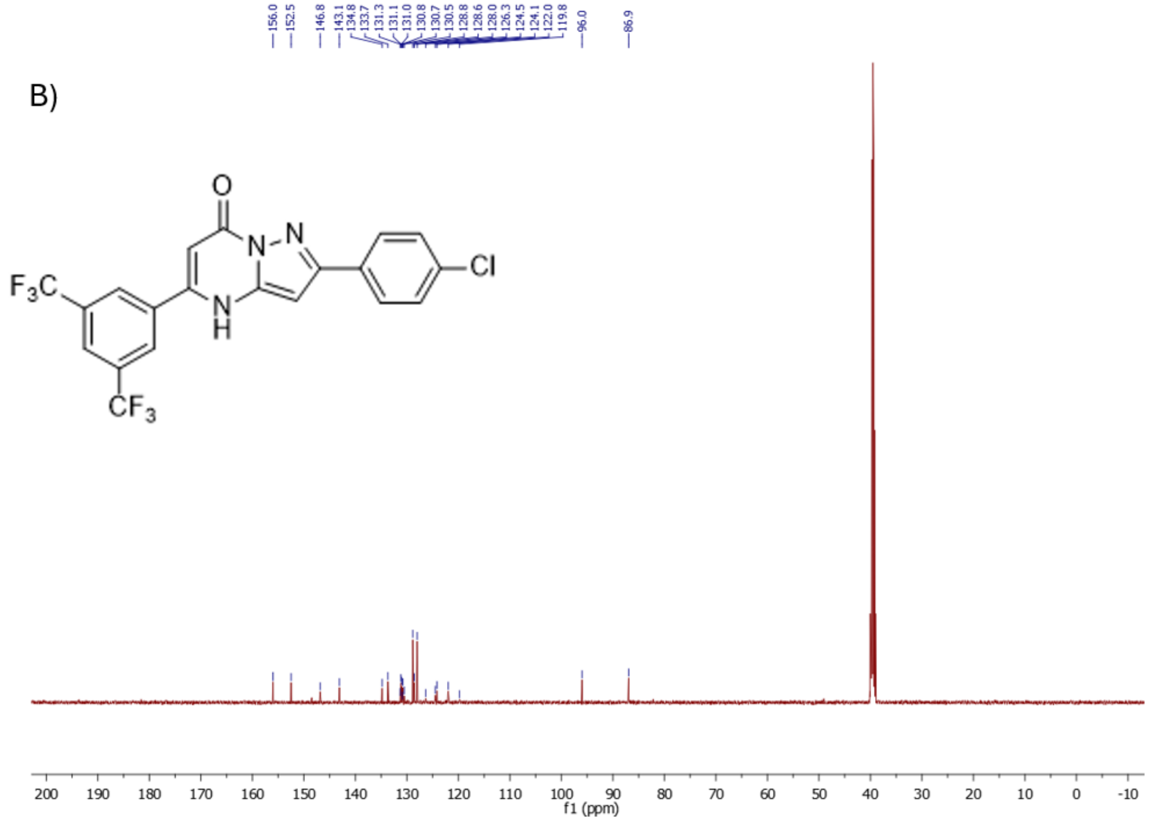
B)
156.0
152.5
146.8
143.1
134.8
133.7
131.3
131.1
131.0
130.8
130.7
130.5
128.8
128.6
128.0
126.3
124.5
124.1
122.0
119.8
96.0
86.9
f1 (ppm)
O
N
N
H
Cl
F3C
CF3

C)

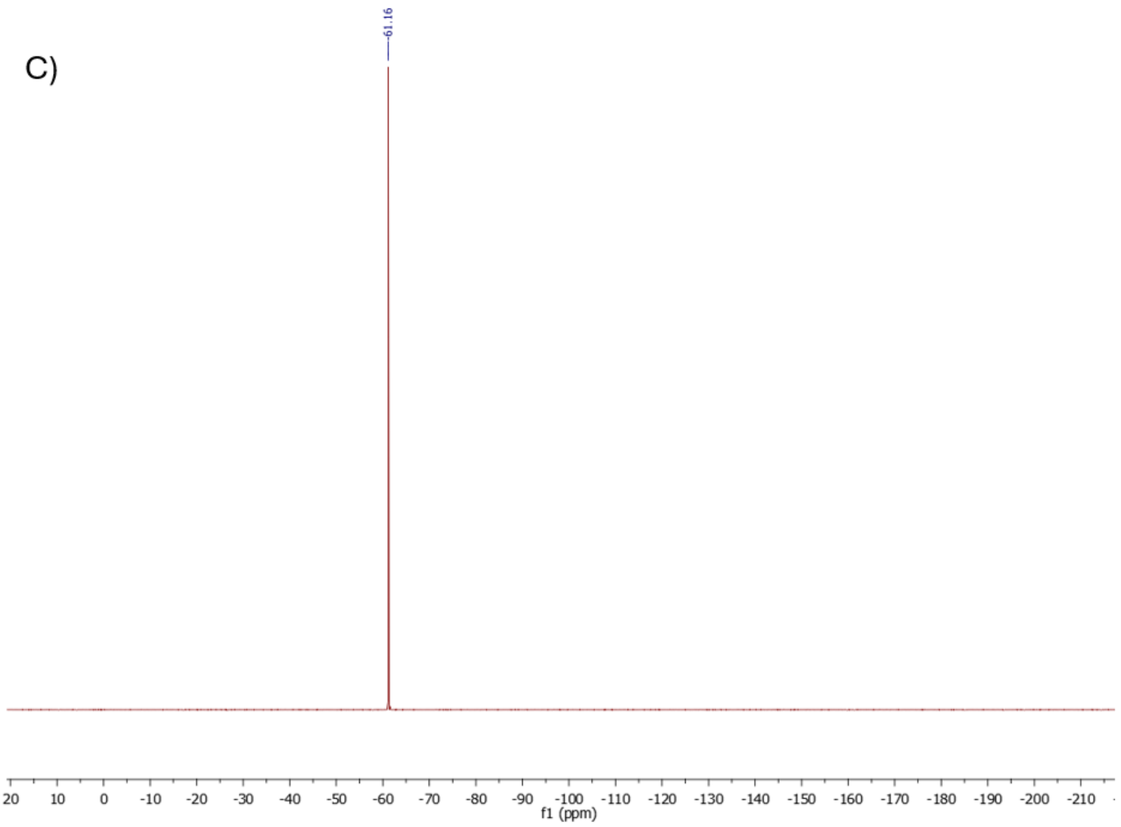


**Figure 15:** $^{1}H$ (500 MHz, DMSO-*d*$_6$) (A) and $^{13}C$ (125 MHz, DMSO-*d*$_6$) (B) $^{19}F$ (471 MHz, DMSO-*d*$_6$) (C) NMR spectra of 5-(3,5-bis(trifluoromethyl)phenyl)-2-(4-chlorophenyl)pyrazolo[1,5-a]pyrimidin-7(4H)-one (**8**).

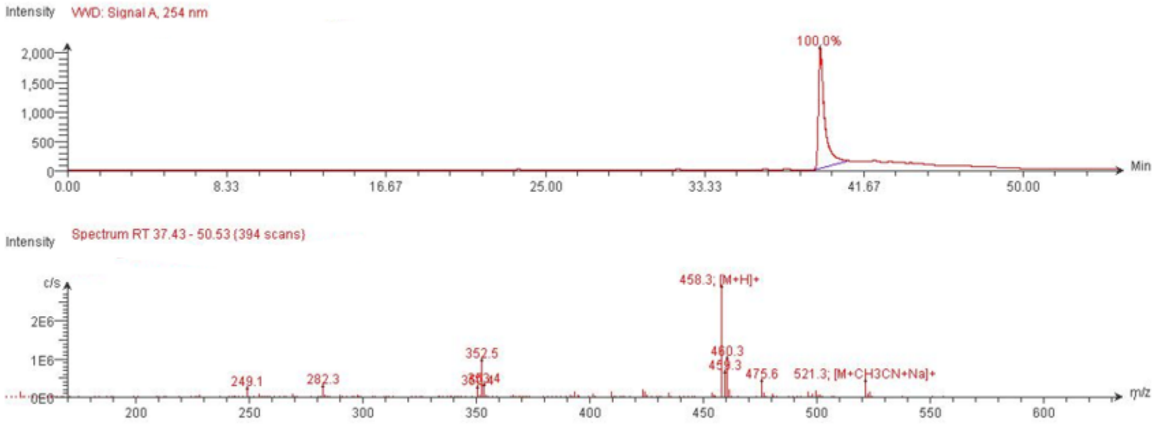


**Figure 16:** LCMS of 5-(3,5-bis(trifluoromethyl)phenyl)-2-(4-chlorophenyl)pyrazolo[1,5-a]pyrimidin-7(4H)-one (**8**), representing its $(M+H)^{+}$ value 458.3.

A)

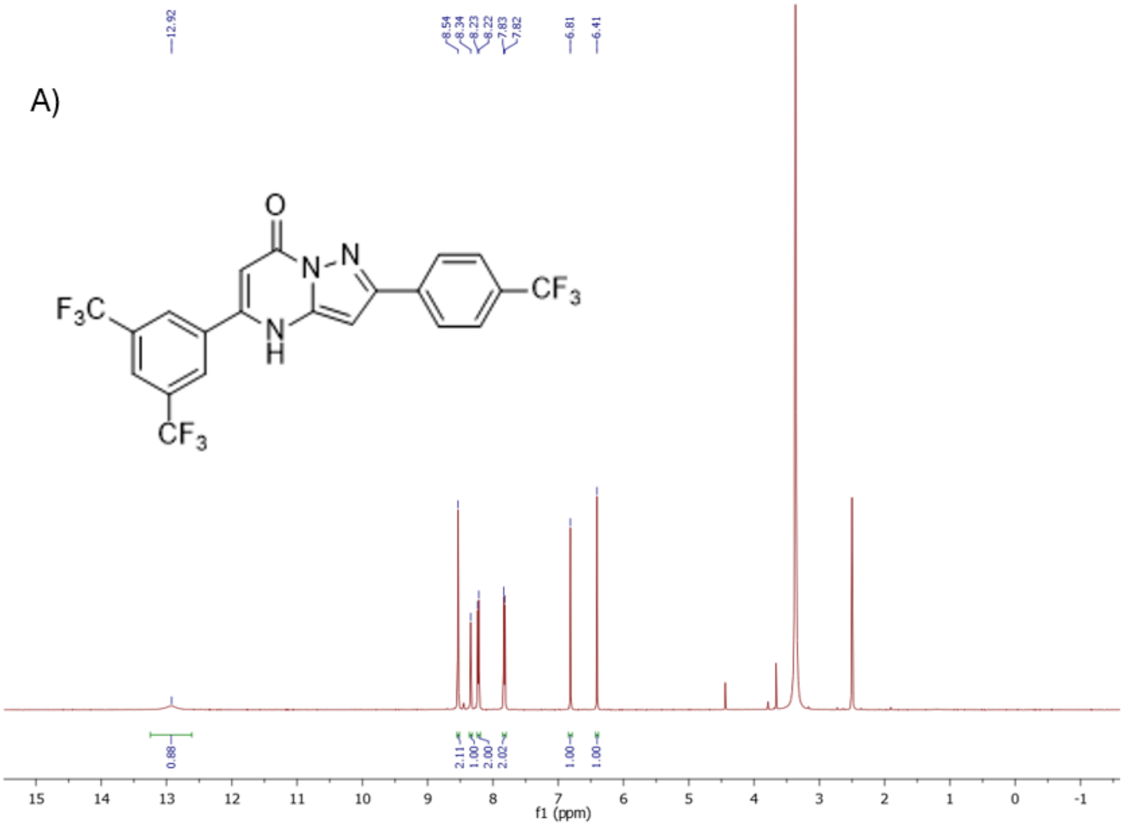
12.92
8.54
8.34
8.23
8.22
7.83
7.82
6.81
6.41
0.88
2.11
1.00
2.00
2.02
1.00
1.00
f1 (ppm)

B)

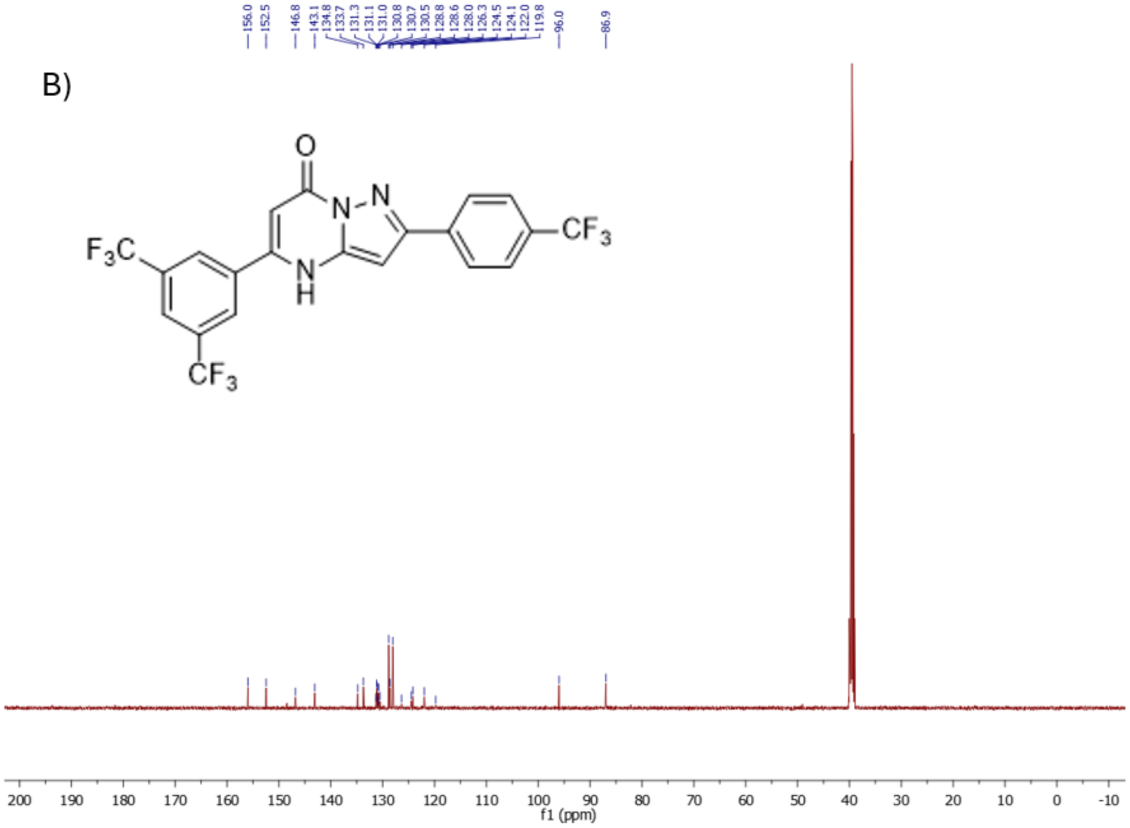
156.0
152.5
146.8
143.1
134.8
133.7
131.3
131.1
131.0
130.8
130.7
130.5
128.8
128.6
128.0
126.3
124.5
124.1
122.0
119.8
96.0
86.9
f1 (ppm)

C)

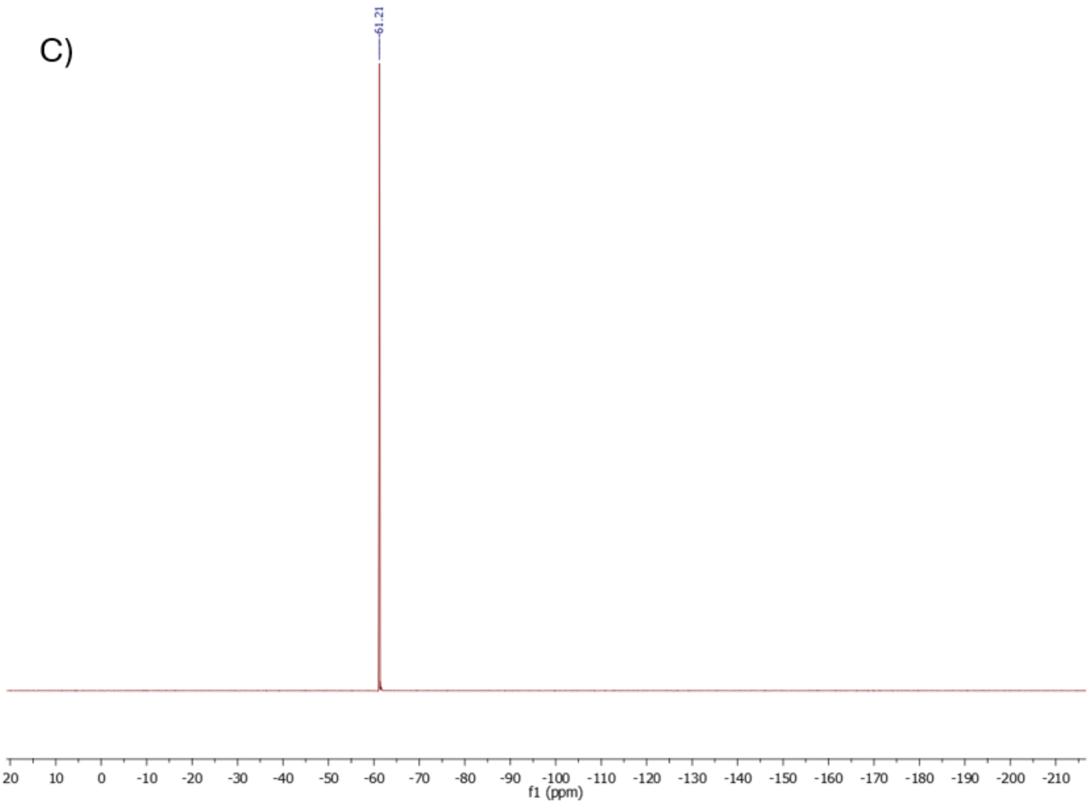


**Figure 17:** $^{1}$H (500 MHz, DMSO-$d_6$) (A) and $^{13}$C (125 MHz, DMSO-$d_6$) (B) $^{19}$F (471 MHz, DMSO-$d_6$) (C) NMR spectra of 5-(3,5-bis(trifluoromethyl)phenyl)-2-(4-(trifluoromethyl)phenyl)pyrazolo[1,5-a]pyrimidin-7(4H)-one (**9**).

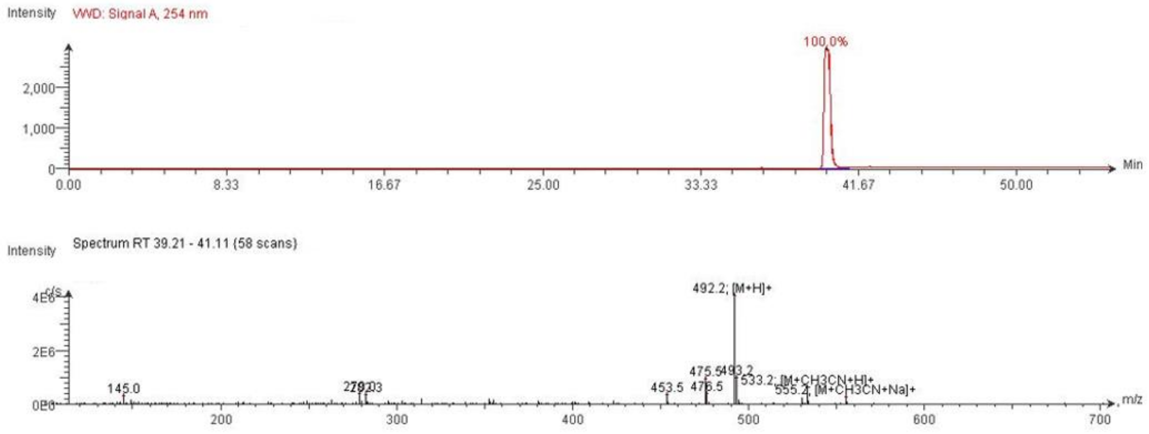


**Figure 18:** LCMS of 5-(3,5-bis(trifluoromethyl)phenyl)-2-(4-(trifluoromethyl)phenyl)pyrazolo[1,5-a]pyrimidin-7(4H)-one (**9**), representing its $(M+H)^+$ value 492.2.

A)

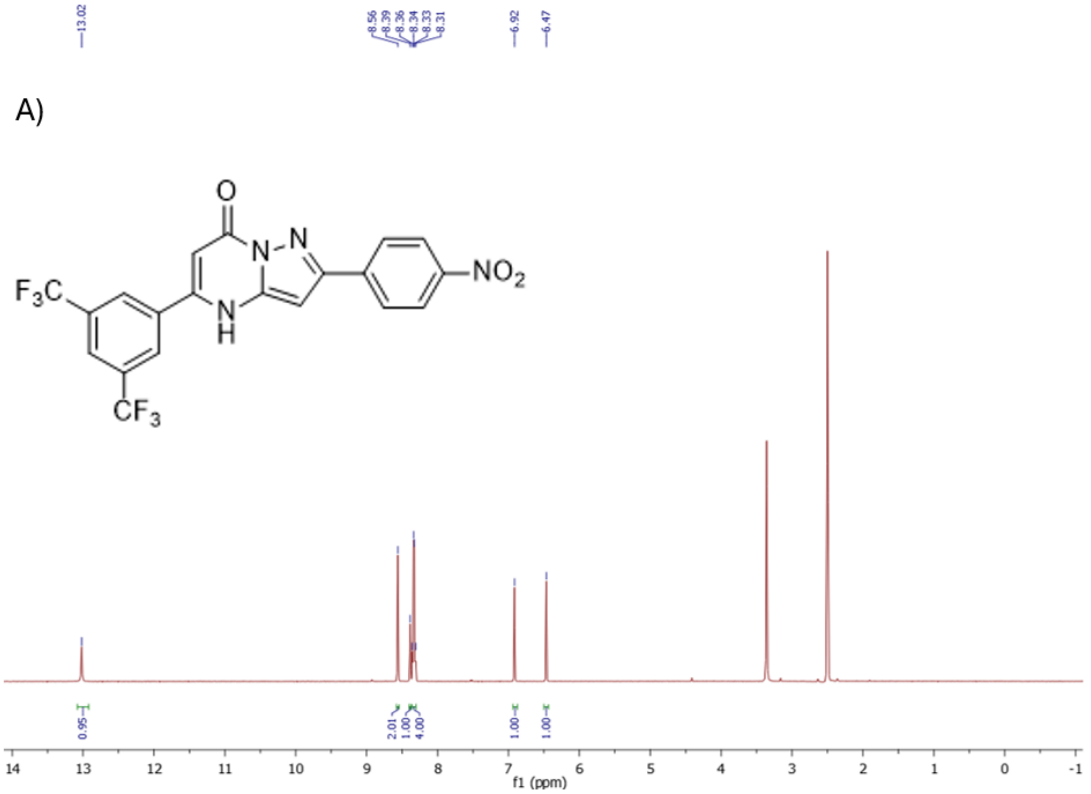
13.02
8.56
8.39
8.36
8.34
8.33
8.31
6.92
6.47
O
N
N
F3C
NO2
N
H
CF3
0.95
2.01
1.00
4.00
1.00
1.00
f1 (ppm)

B)

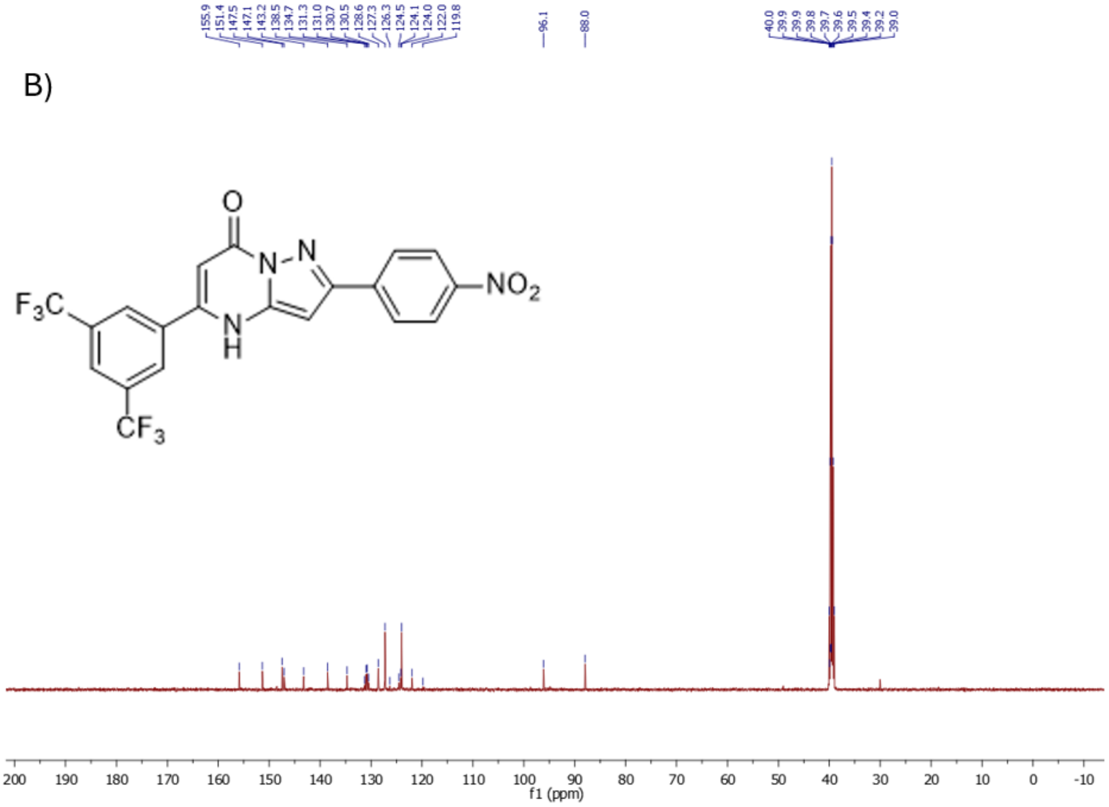
155.9
151.4
147.5
147.1
143.2
138.5
134.7
131.3
131.0
130.7
130.5
128.6
127.3
126.3
124.5
124.1
124.0
122.0
119.8
96.1
88.0
40.0
39.9
39.9
39.8
39.7
39.6
39.5
39.4
39.2
39.0
O
N
N
F3C
NO2
N
H
CF3
f1 (ppm)

C)

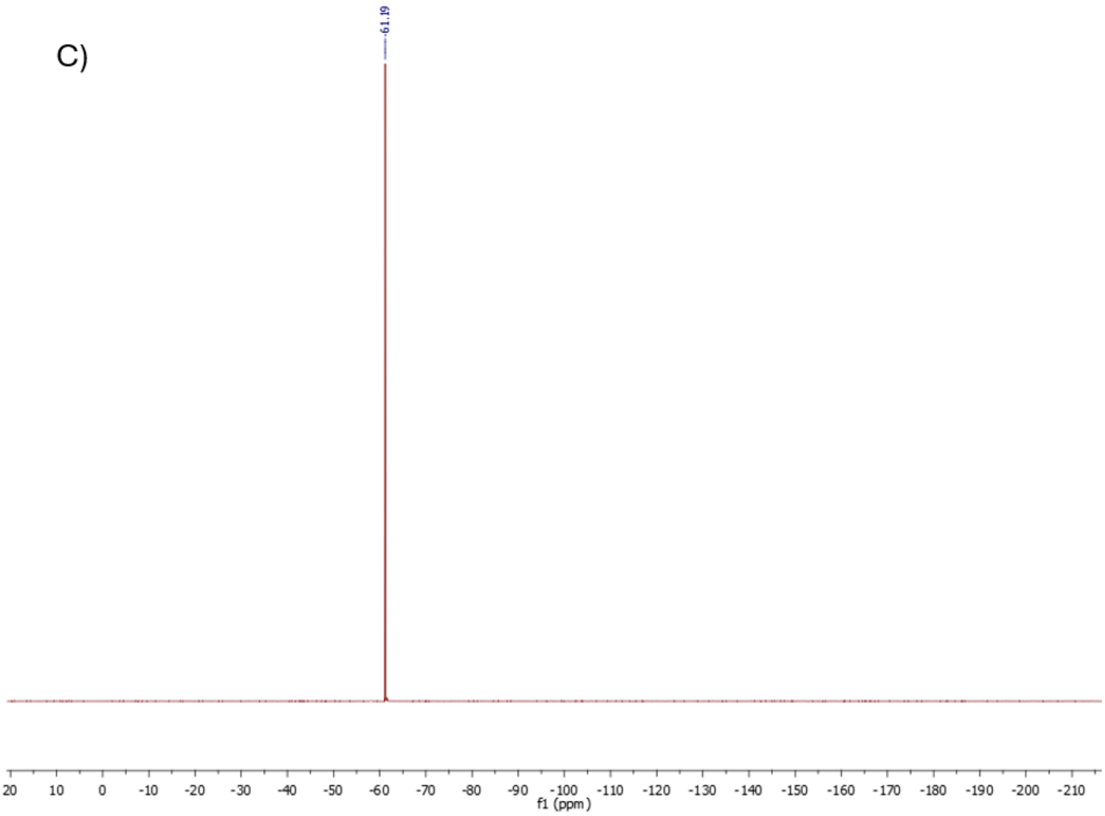


**Figure 19:** $^{1}H$ (500 MHz, DMSO-$d_6$) (A) and $^{13}C$ (125 MHz, DMSO-$d_6$) (B) $^{19}F$ (471 MHz, DMSO-$d_6$) (C) NMR spectra of 5-(3,5-bis(trifluoromethyl)phenyl)-2-(4-nitrophenyl)pyrazolo[1,5-a]pyrimidin-7(4H)-one (**10**).

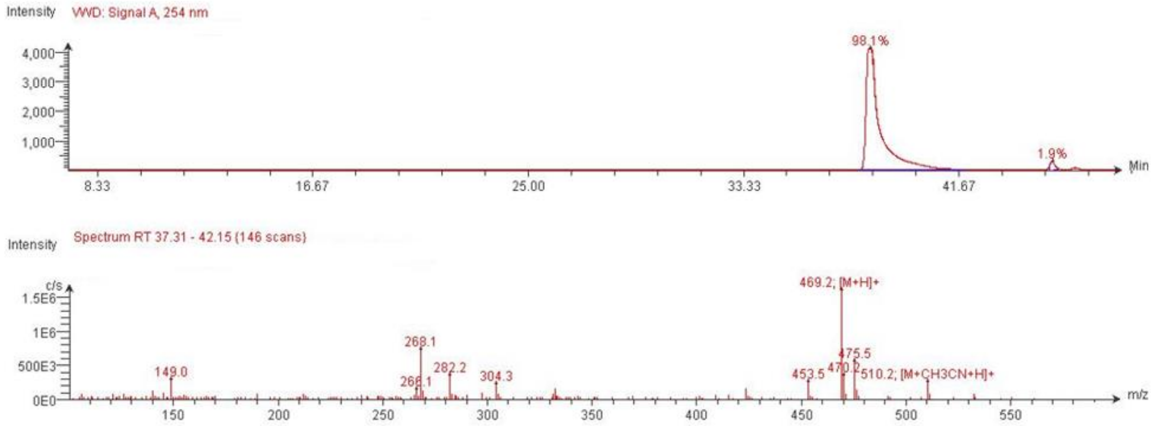


**Figure 20:** LCMS of 5-(3,5-bis(trifluoromethyl)phenyl)-2-(4-nitrophenyl)pyrazolo[1,5-a]pyrimidin-7(4H)-one (**10**), representing its $(M+H)^{+}$ value 469.2.

A)

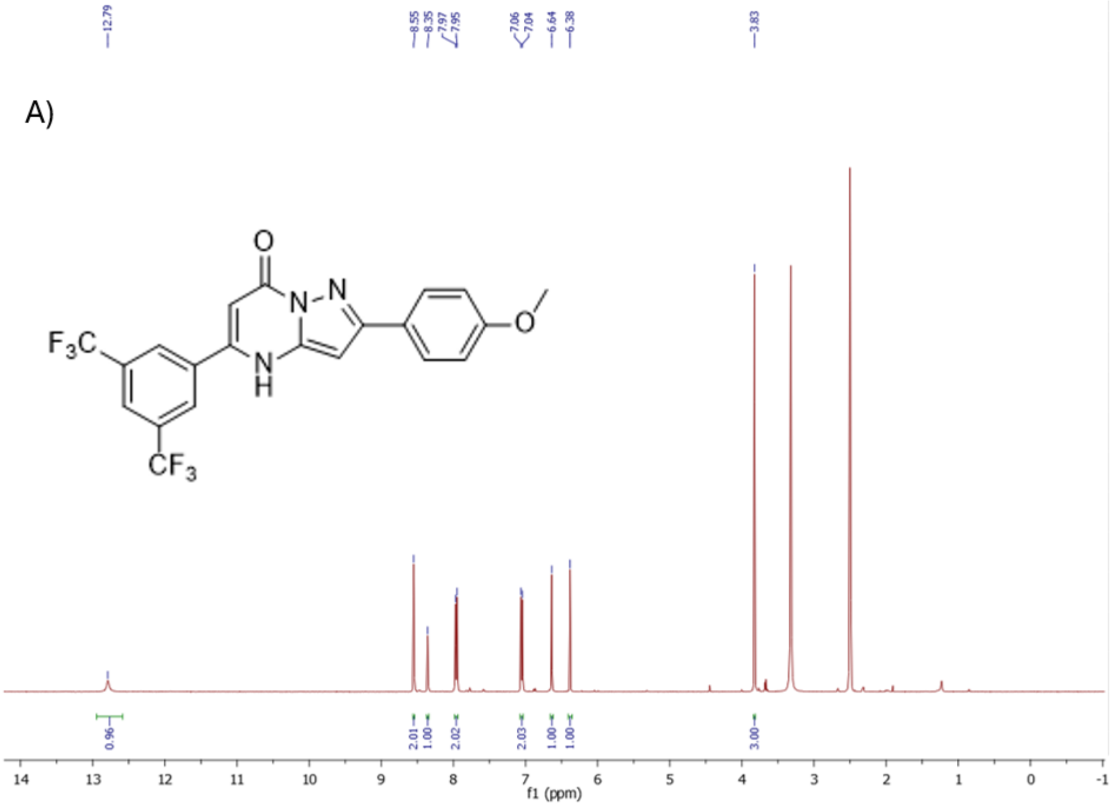
12.79
8.55
8.35
7.97
7.95
7.06
7.04
6.64
6.38
3.83
O
N
N
F3C
N
H
O
CF3
0.96
2.01
1.00
2.02
2.03
1.00
1.00
3.00
14 13 12 11 10 9 8 7 6 5 4 3 2 1 0 -1
f1 (ppm)

B)

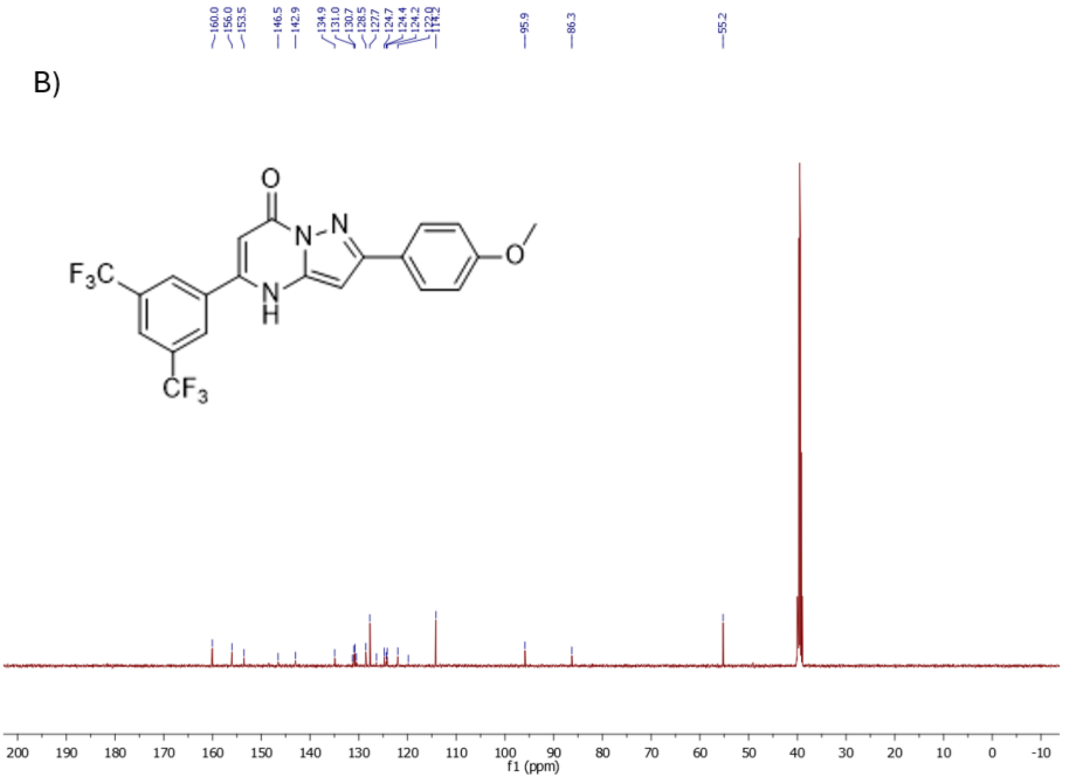
160.0
156.0
153.5
146.5
142.9
134.9
131.0
130.7
128.5
127.7
124.7
124.4
124.2
122.0
114.2
95.9
86.3
55.2
O
N
N
F3C
N
H
O
CF3
200 190 180 170 160 150 140 130 120 110 100 90 80 70 60 50 40 30 20 10 0 -10
f1 (ppm)

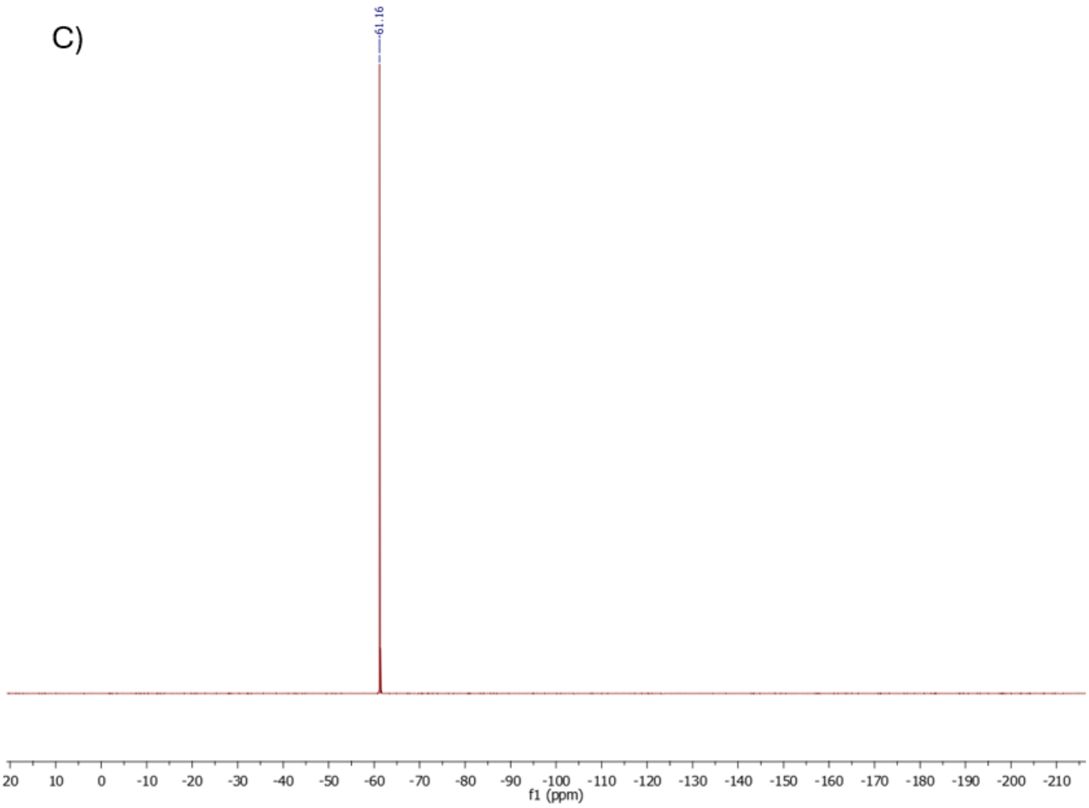


**Figure 21:** $^{1}$H (500 MHz, DMSO-$d_6$) (A) and $^{13}$C (125 MHz, DMSO-$d_6$) (B) $^{19}$F (471 MHz, DMSO-$d_6$) (C) NMR spectra of 5-(3,5-bis(trifluoromethyl)phenyl)-2-(4-methoxyphenyl)pyrazolo[1,5-a]pyrimidin-7(4H)-one (**11**).

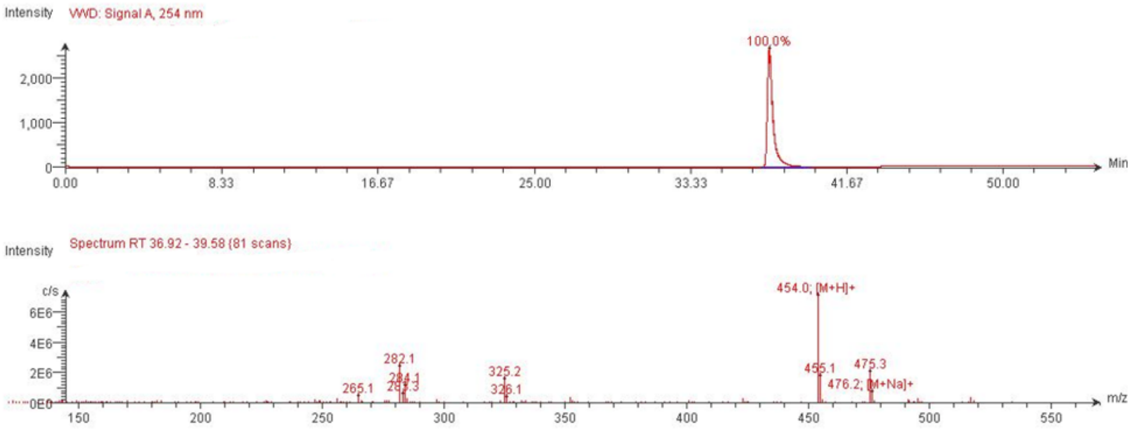


**Figure 22:** LCMS of 5-(3,5-bis(trifluoromethyl)phenyl)-2-(4-methoxyphenyl)pyrazolo[1,5-a]pyrimidin-7(4H)-one (**11**), representing its $(M+H)^+$ value 454.0.

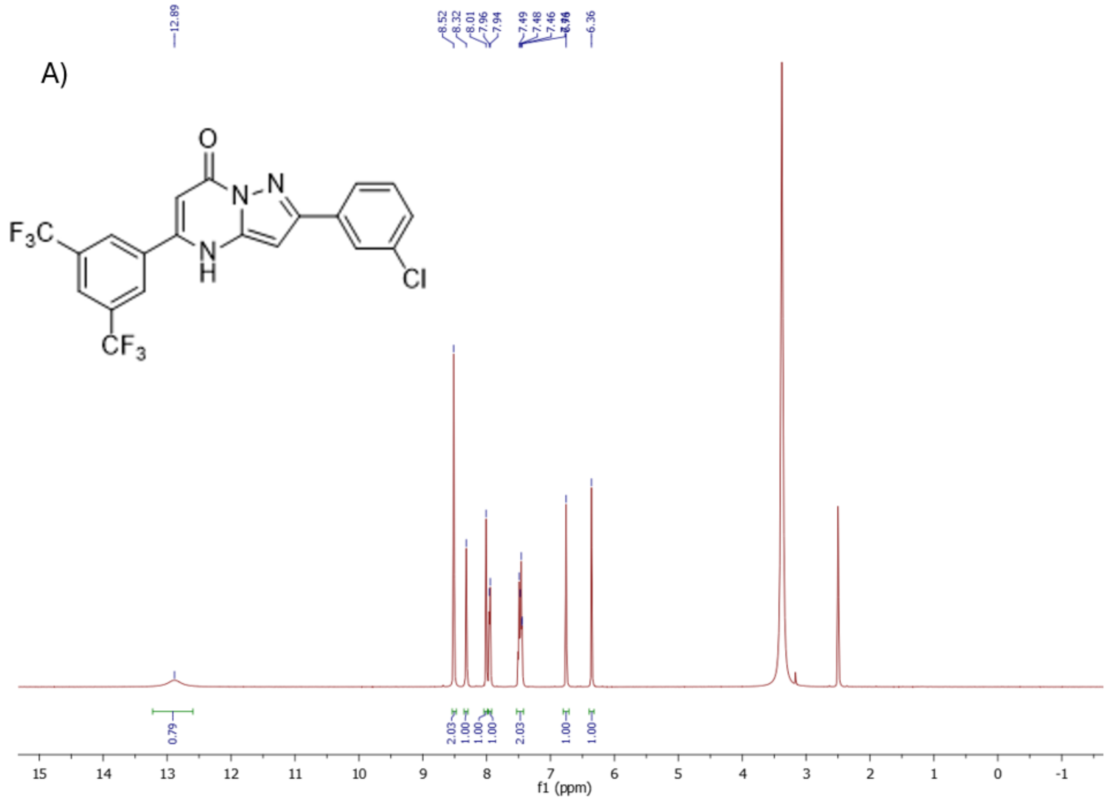
A)
12.89
8.52
8.32
8.01
7.96
7.94
7.49
7.48
7.46
6.36
0.79
2.03
1.00
1.00
1.00
2.03
1.00
1.00
f1 (ppm)

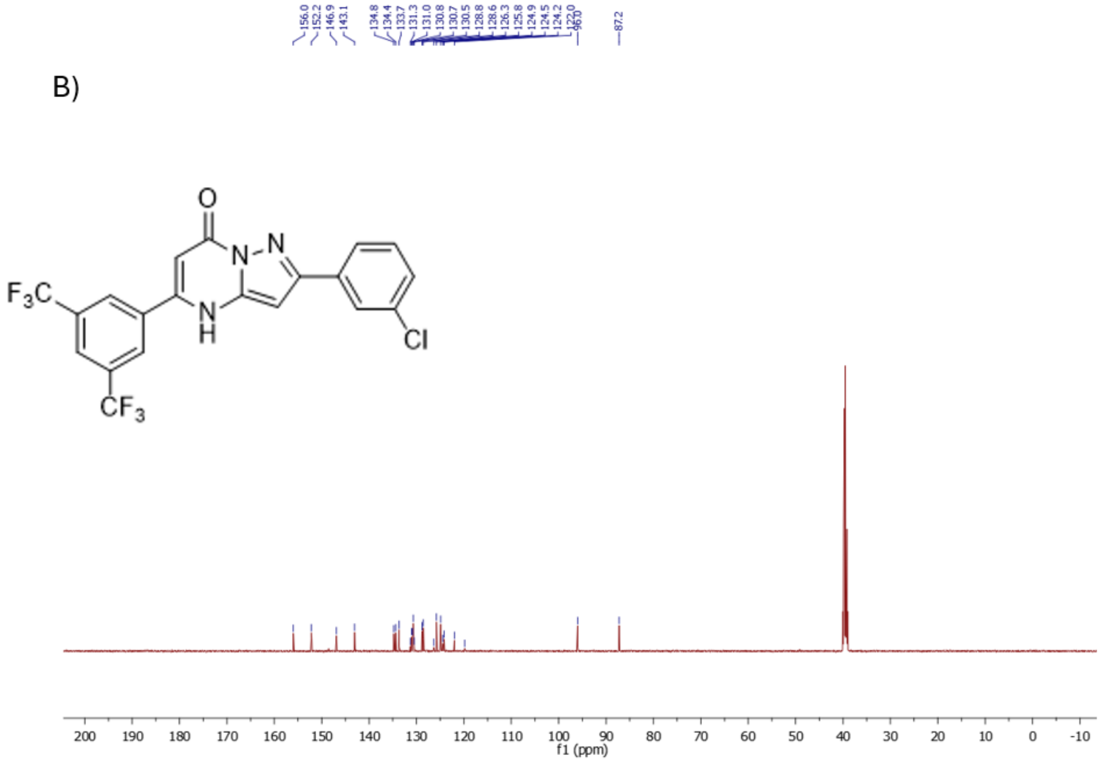
B)
156.0
152.2
146.9
143.1
134.8
134.4
133.7
131.3
131.0
130.8
130.7
130.5
128.8
128.6
126.3
125.8
124.9
124.5
124.2
122.0
96.0
87.2
f1 (ppm)

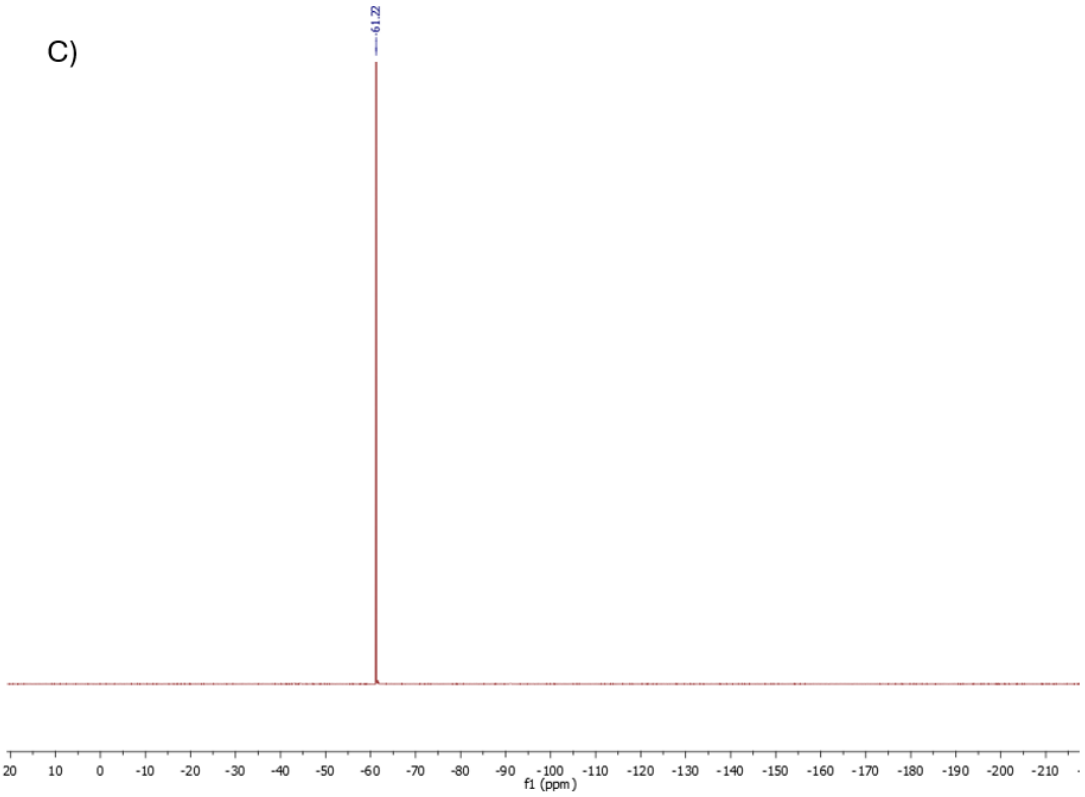


**Figure 23:** $^{1}H$ (500 MHz, DMSO-$d_6$) (A) and $^{13}C$ (125 MHz, DMSO-$d_6$) (B) $^{19}F$ (471 MHz, DMSO-$d_6$) (C) NMR spectra of 5-(3,5-bis(trifluoromethyl)phenyl)-2-(3-chlorophenyl)pyrazolo[1,5-a]pyrimidin-7(4H)-one (**12**).

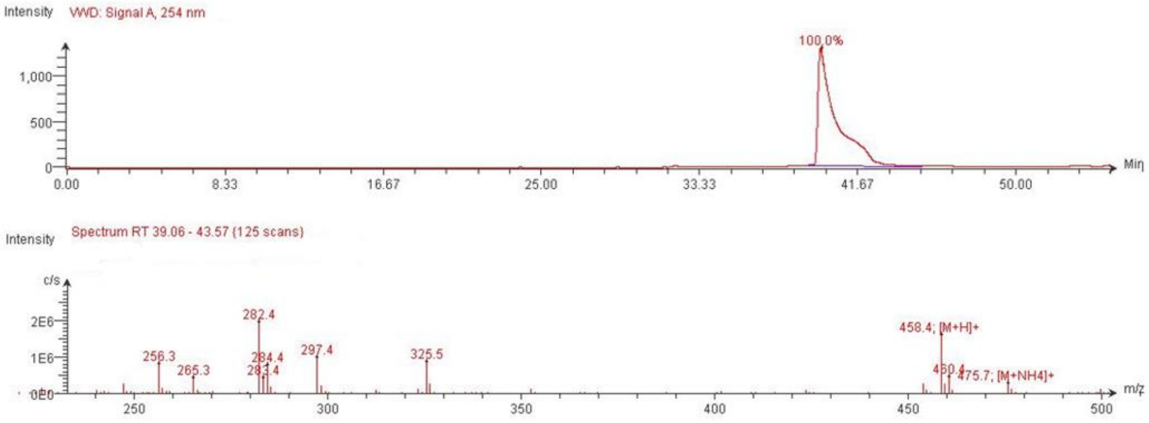


**Figure 24:** LCMS of 5-(3,5-bis(trifluoromethyl)phenyl)-2-(3-chlorophenyl)pyrazolo[1,5-a]pyrimidin-7(4H)-one (**12**), representing its $(M+H)^+$ value 458.4.

A)

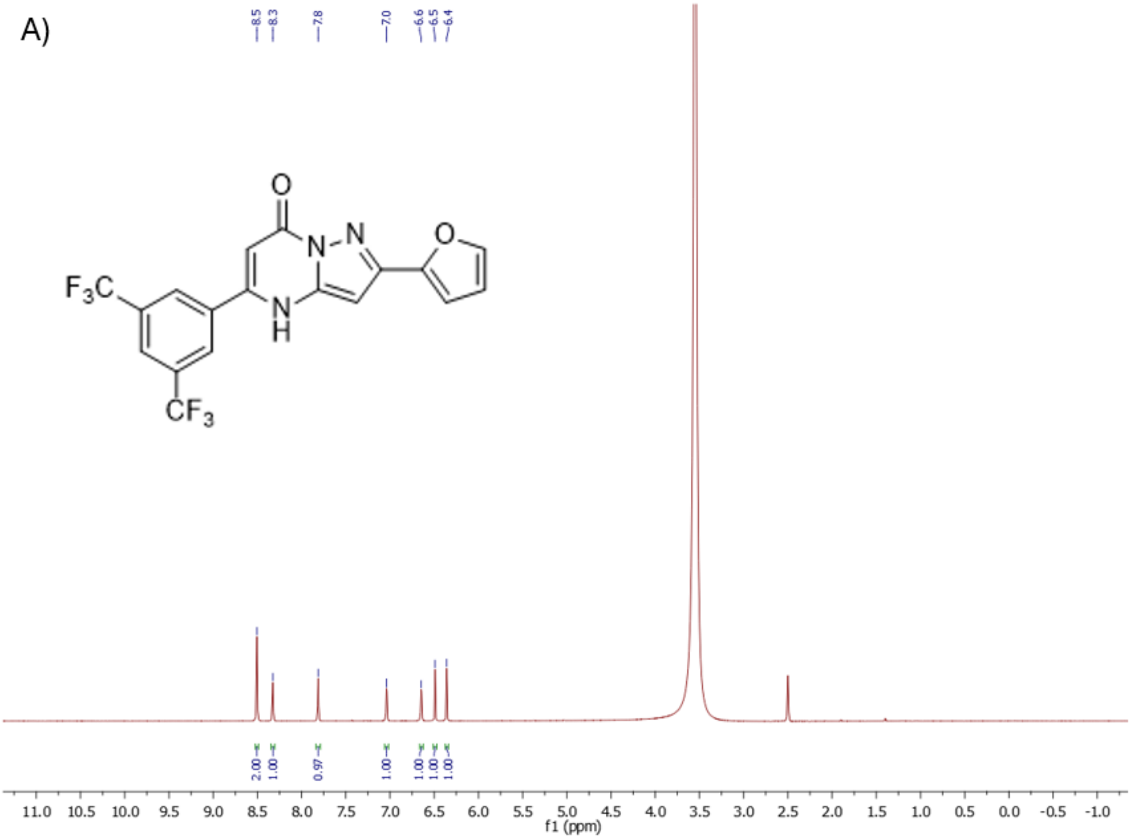
8.5
8.3
7.8
7.0
6.6
6.5
6.4
O
N
N
O
F3C
N
H
CF3
2.00
1.00
0.97
1.00
1.00
1.00
1.00
11.0 10.5 10.0 9.5 9.0 8.5 8.0 7.5 7.0 6.5 6.0 5.5 5.0 4.5 4.0 3.5 3.0 2.5 2.0 1.5 1.0 0.5 0.0 -0.5 -1.0
f1 (ppm)

B)

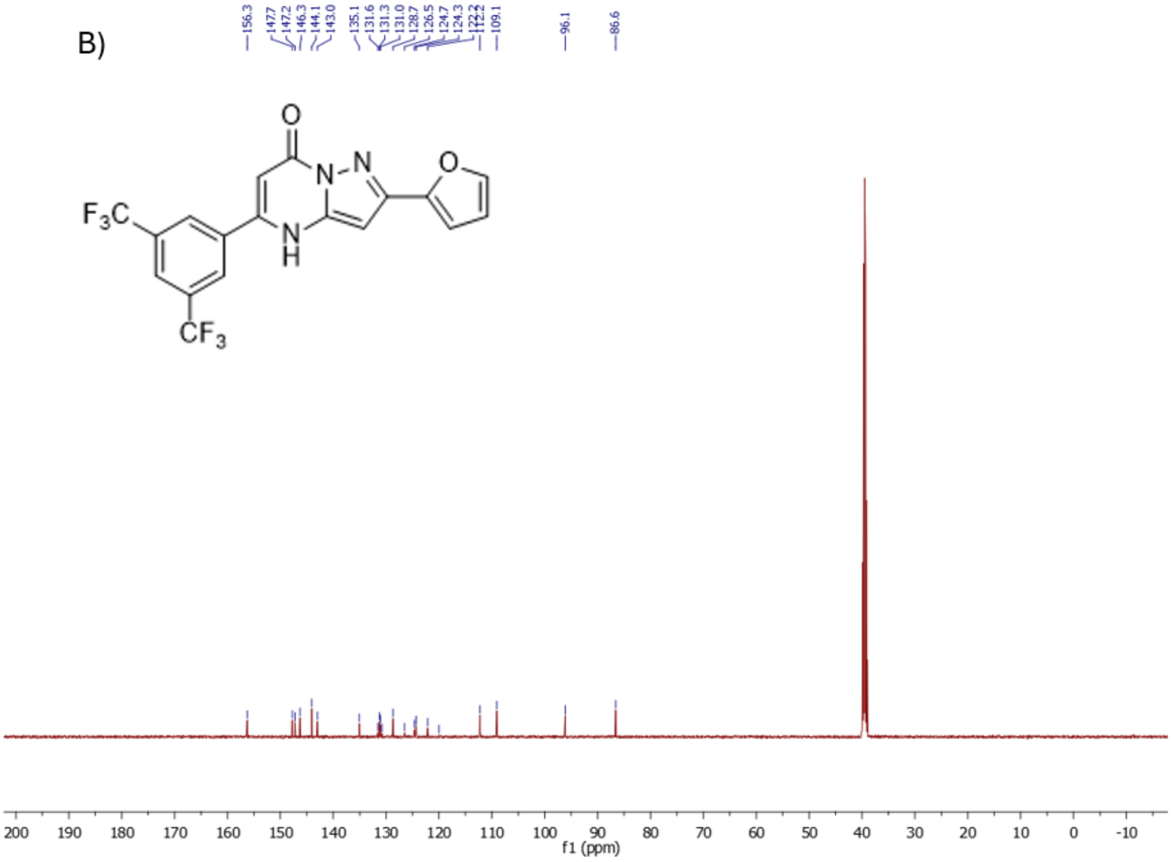
156.3
147.7
147.2
146.3
144.1
143.0
135.1
131.6
131.3
131.0
128.7
126.5
124.7
124.3
122.2
112.2
109.1
96.1
86.6
O
N
N
O
F3C
N
H
CF3
200 190 180 170 160 150 140 130 120 110 100 90 80 70 60 50 40 30 20 10 0 -10
f1 (ppm)

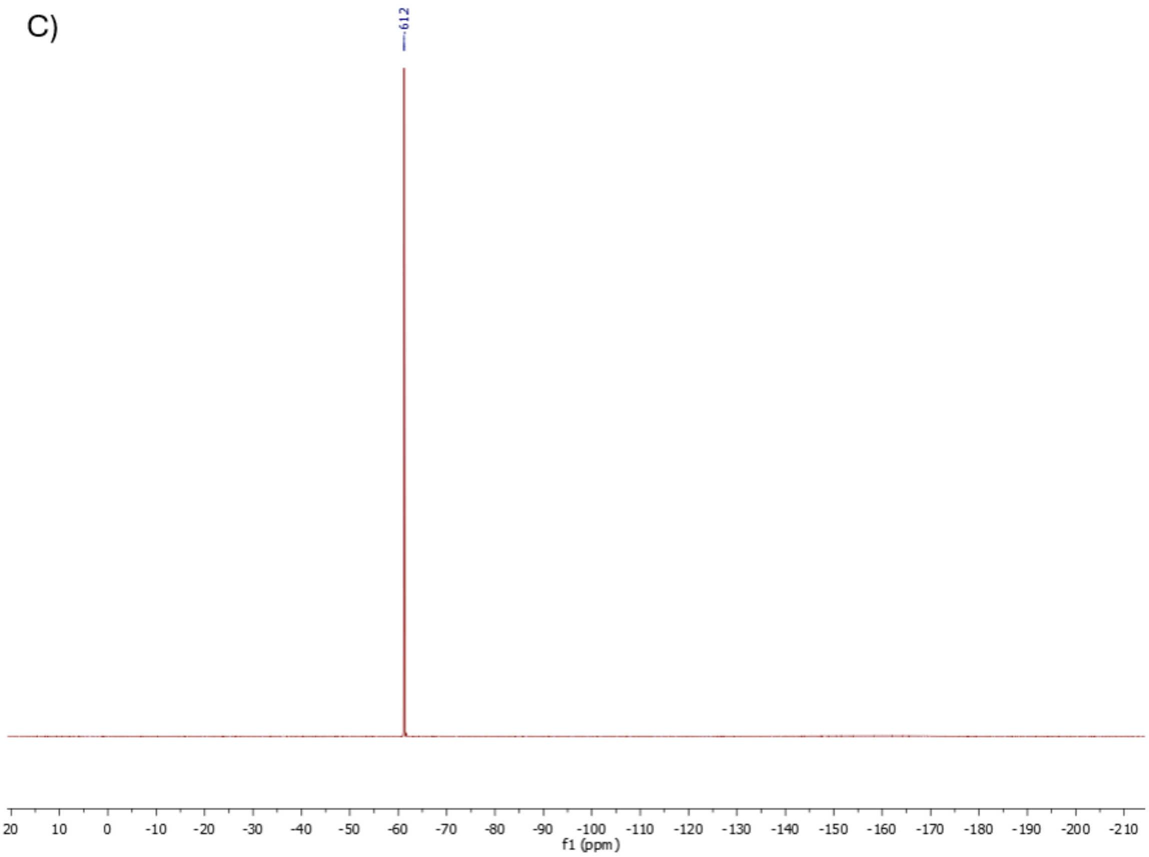


**Figure 25:** $^{1}H$ (500 MHz, DMSO-$d_6$) (A) and $^{13}C$ (125 MHz, DMSO-$d_6$) (B) $^{19}F$ (471 MHz, DMSO-$d_6$) (C) NMR spectra of 5-(3,5-bis(trifluoromethyl)phenyl)-2-(furan-2-yl)pyrazolo[1,5-a]pyrimidin-7(4H)-one (**13**).

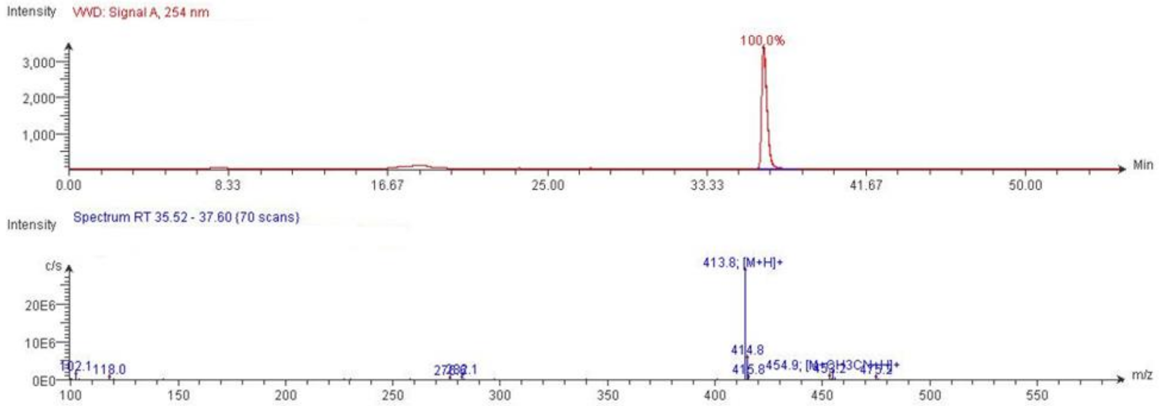


**Figure 26:** LCMS of 5-(3,5-bis(trifluoromethyl)phenyl)-2-(furan-2-yl)pyrazolo[1,5-a]pyrimidin-7(4H)-one (**13**), representing its $(M+H)^+$ value 413.8.

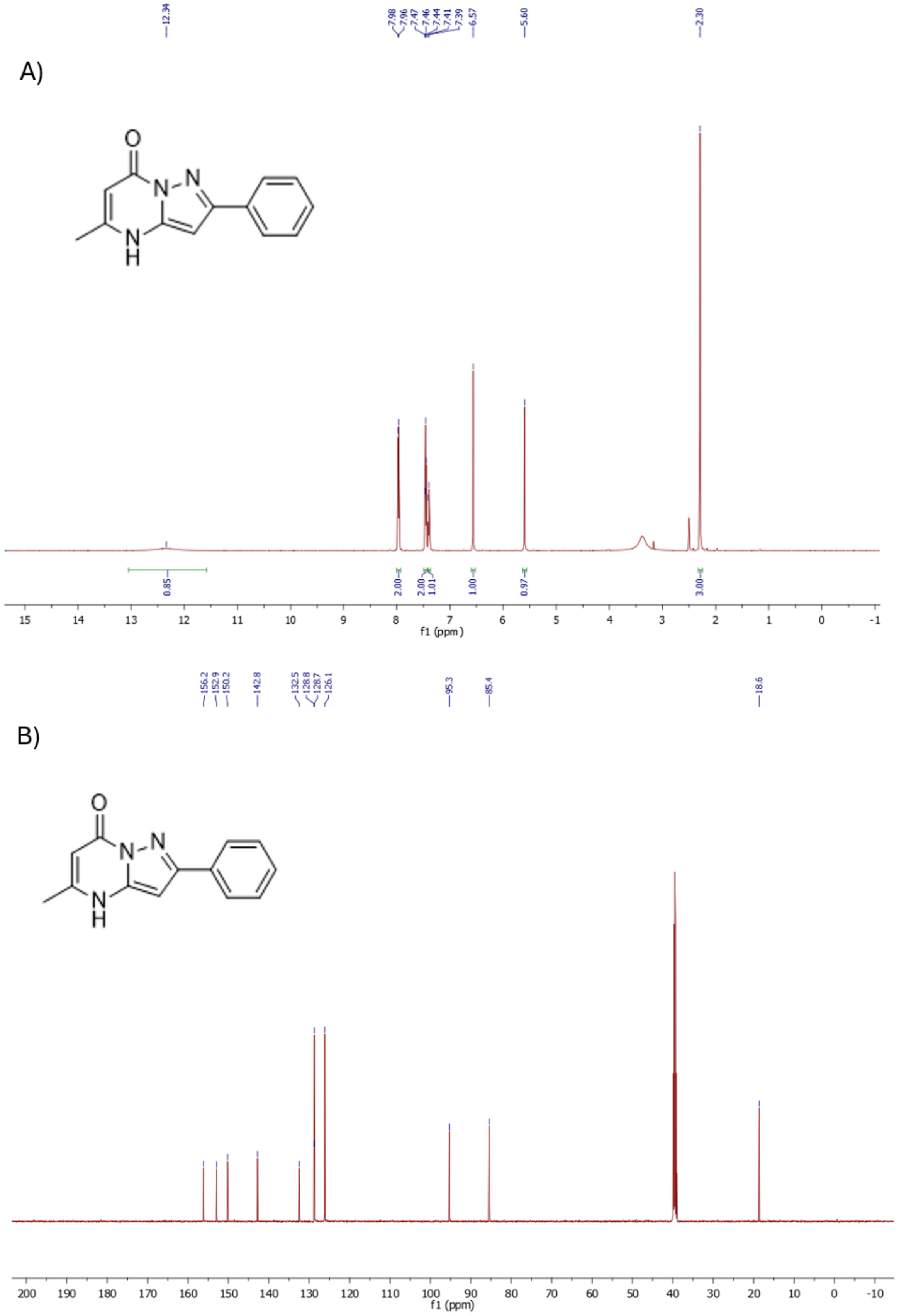


**Figure 27:** $^{1}H$ (500 MHz, DMSO-$d_6$) (A) and $^{13}C$ (125 MHz, DMSO-$d_6$) (B) NMR spectra of 5-methyl-2-phenylpyrazolo[1,5-a]pyrimidin-7(4H)-one (**14**).

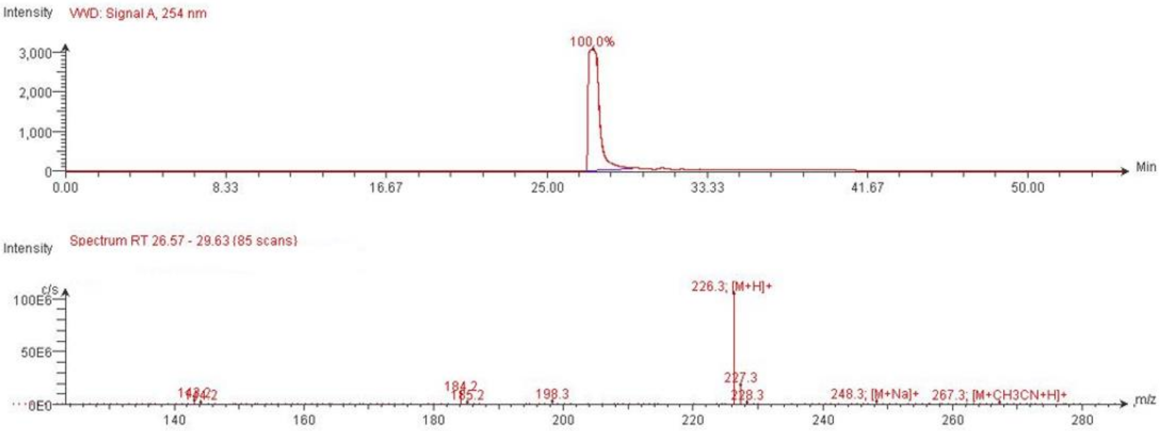


**Figure 28:** LCMS of 5-methyl-2-phenylpyrazolo[1,5-a]pyrimidin-7(4H)-one (**14**), representing its $(M+H)^+$ value 226.3.

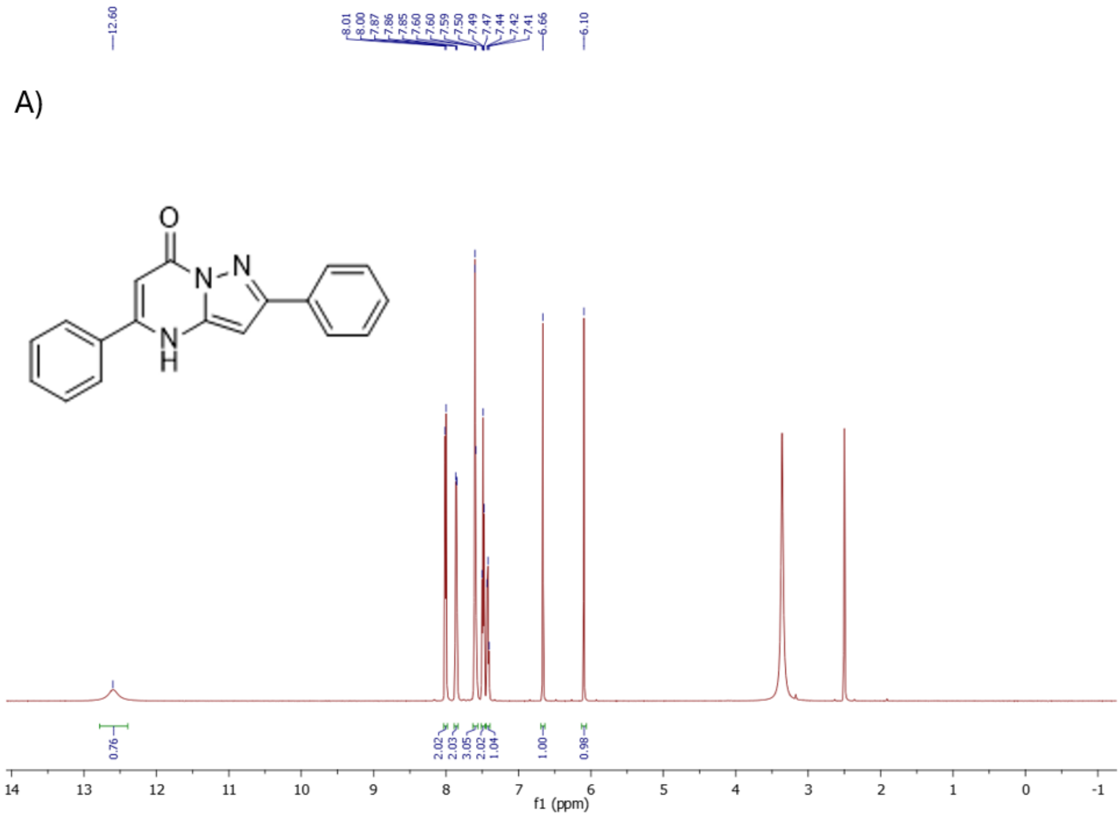


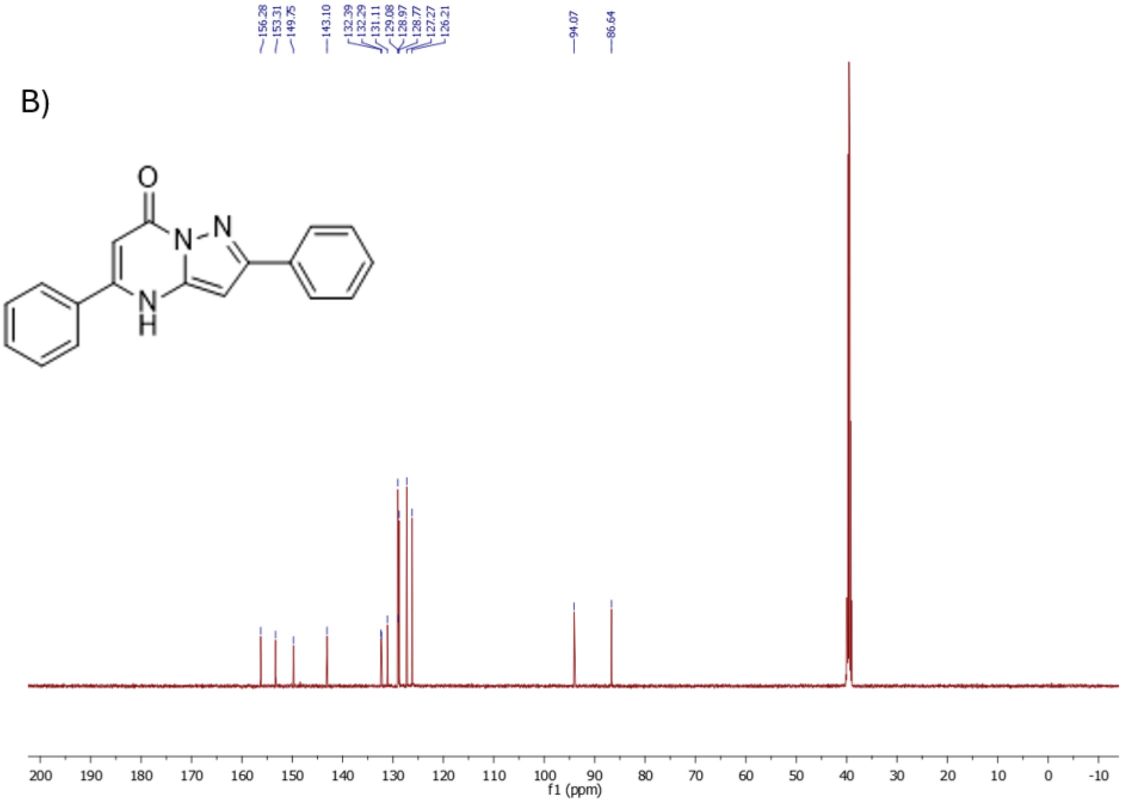


**Figure 29:** $^{1}H$ (500 MHz, DMSO-$d_6$) (A) and $^{13}C$ (125 MHz, DMSO-$d_6$) (B) NMR spectra of 2,5-diphenylpyrazolo[1,5-a]pyrimidin-7(4H)-one (**15**).

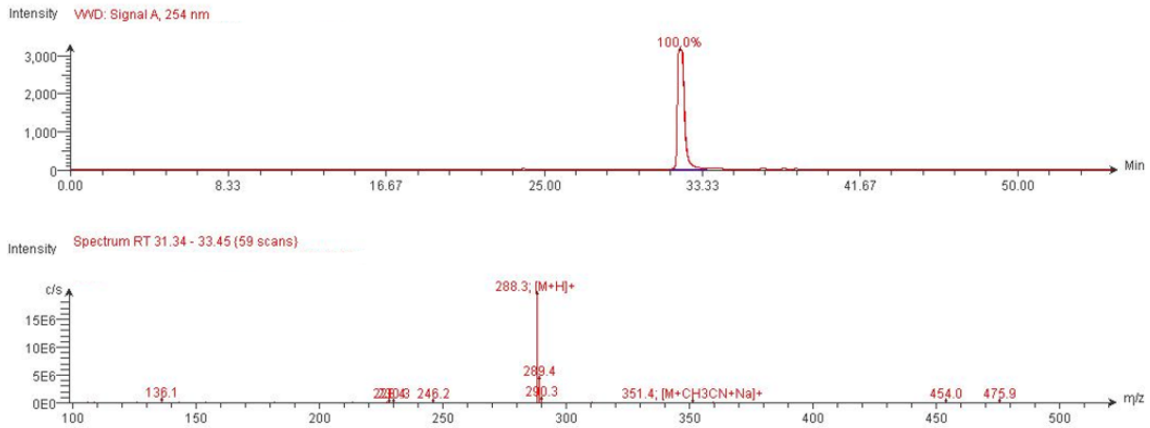


**Figure 30:** LCMS of 2,5-diphenylpyrazolo[1,5-a]pyrimidin-7(4H)-one (**15**), representing its $(M+H)^+$ value 288.3.

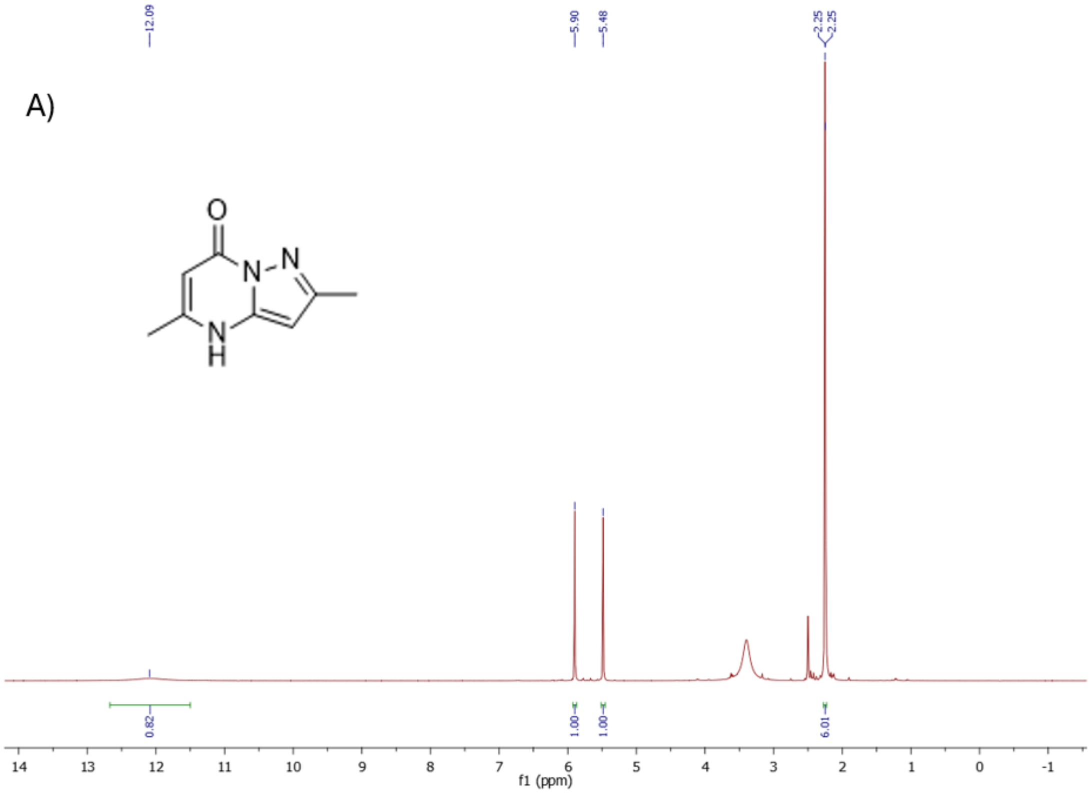



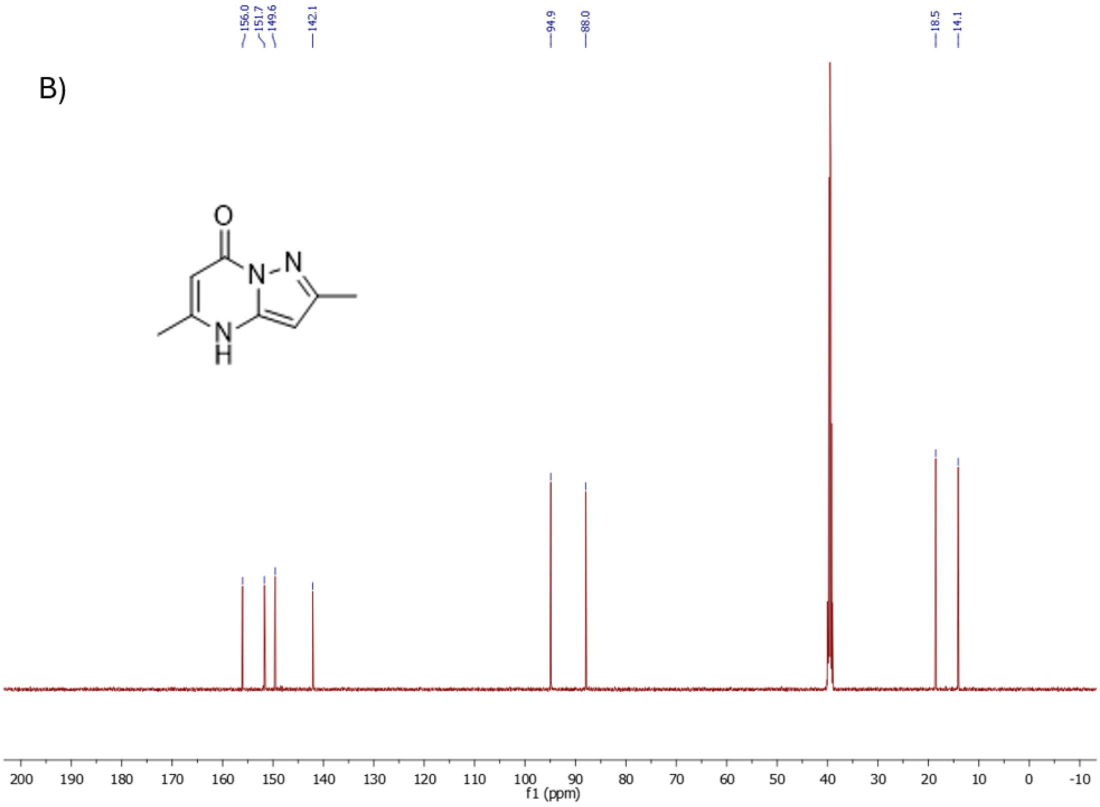



**Figure 31:** $^{1}H$ (500 MHz, DMSO-$d_6$) (A) and $^{13}C$ (125 MHz, DMSO-$d_6$) (B) NMR spectra of 2,5-dimethylpyrazolo[1,5-a]pyrimidin-7(4H)-one (**16**).

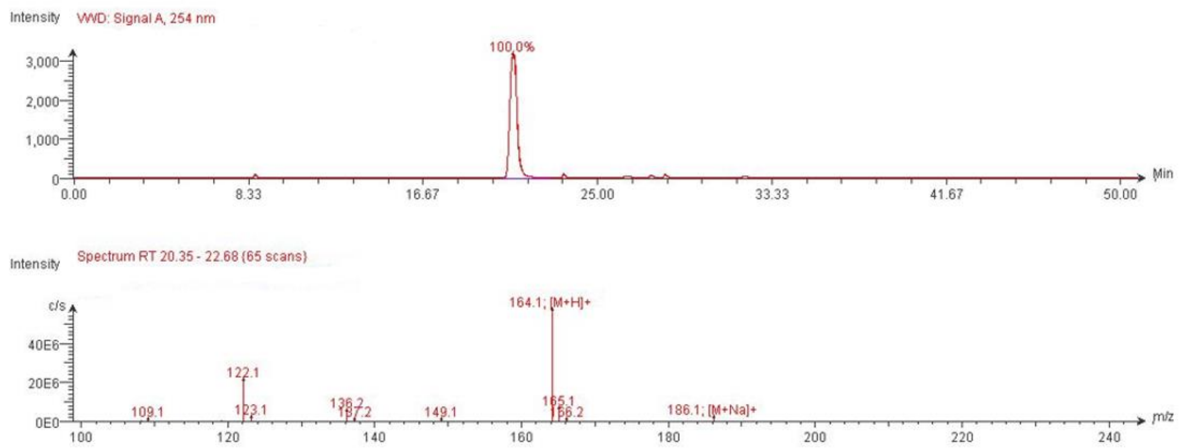


**Figure 32:** LCMS of 2,5-dimethylpyrazolo[1,5-a]pyrimidin-7(4H)-one (**16**), representing its $(M+H)^+$ value 164.1.

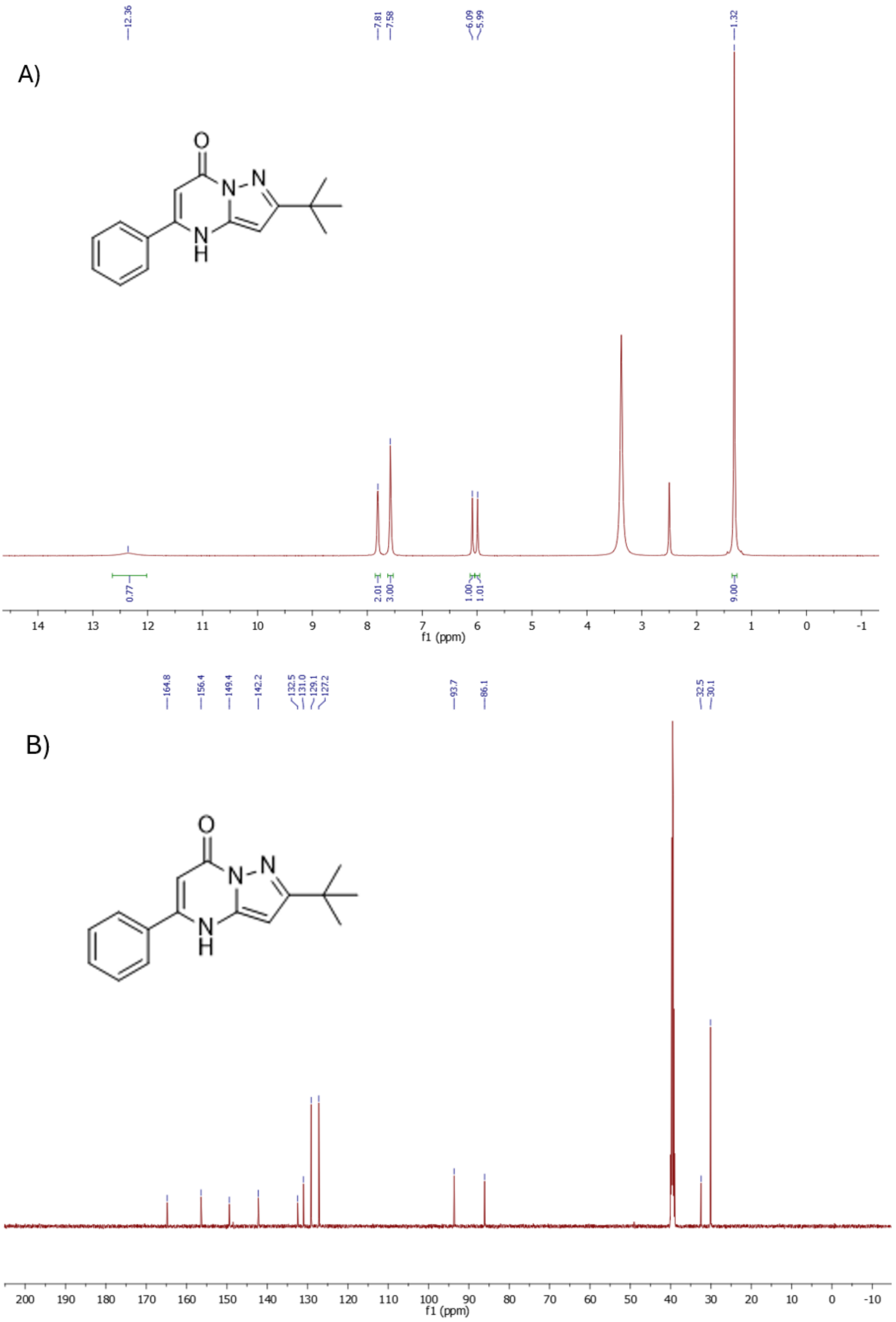


**Figure 33:** $^{1}H$ (500 MHz, DMSO-$d_6$) (A) and $^{13}C$ (125 MHz, DMSO-$d_6$) (B) NMR spectra of 2-(tert-butyl)-5-phenylpyrazolo[1,5-a]pyrimidin-7(4H)-one (**17**).

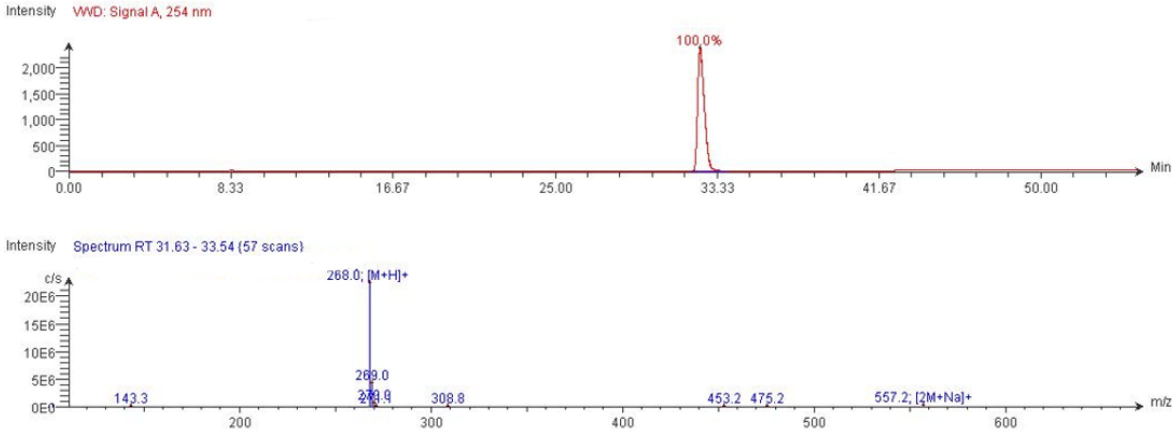


**Figure 34:** LCMS of 2-(tert-butyl)-5-phenylpyrazolo[1,5-a]pyrimidin-7(4H)-one (**17**), representing its $(M+H)^+$ value 268.0.

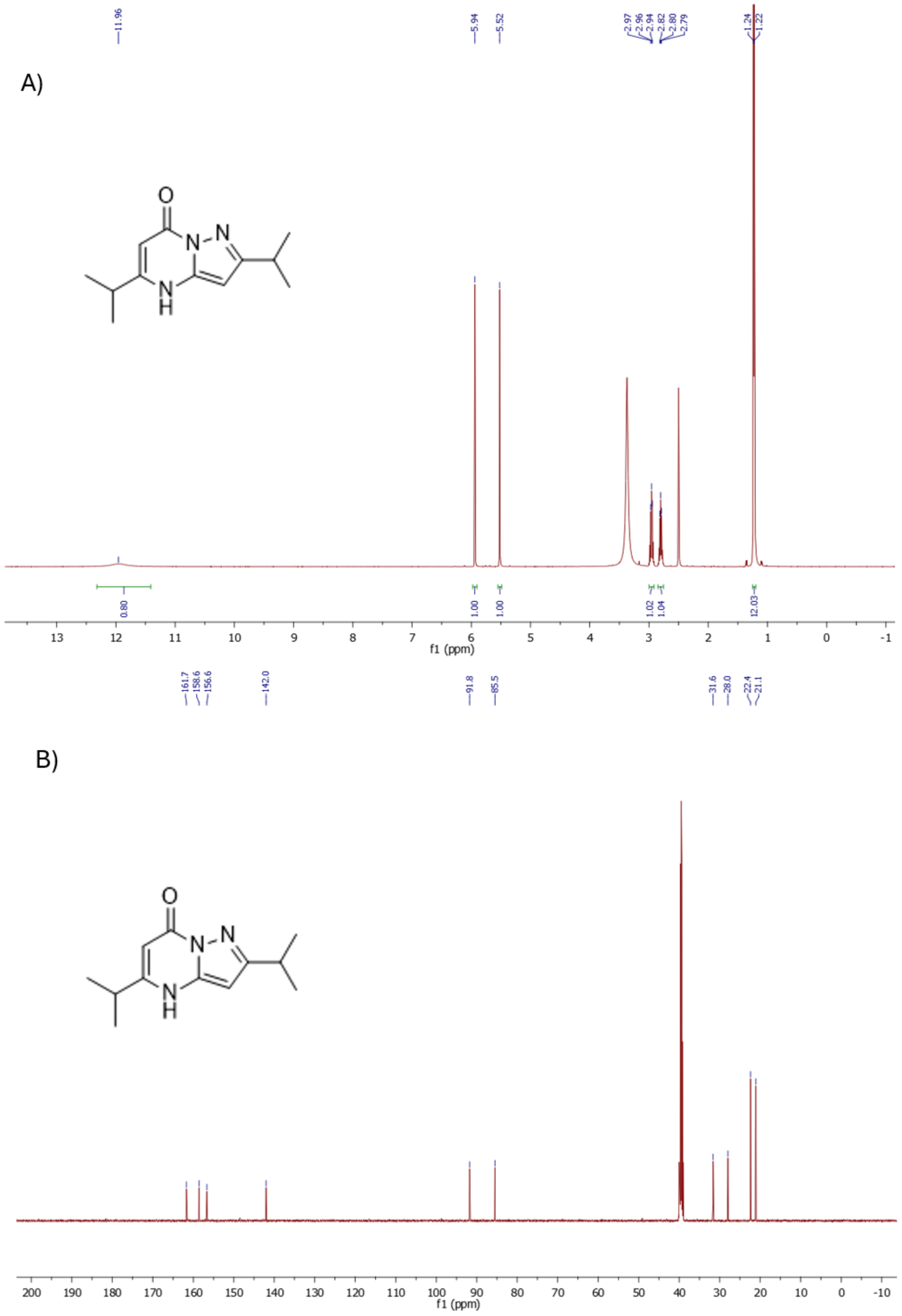


**Figure 35:** $^{1}$H (500 MHz, DMSO-$d_6$) (A) and $^{13}$C (125 MHz, DMSO-$d_6$) (B) NMR spectra of 2,5-diisopropylpyrazolo[1,5-a]pyrimidin-7(4H)-one (**18**).

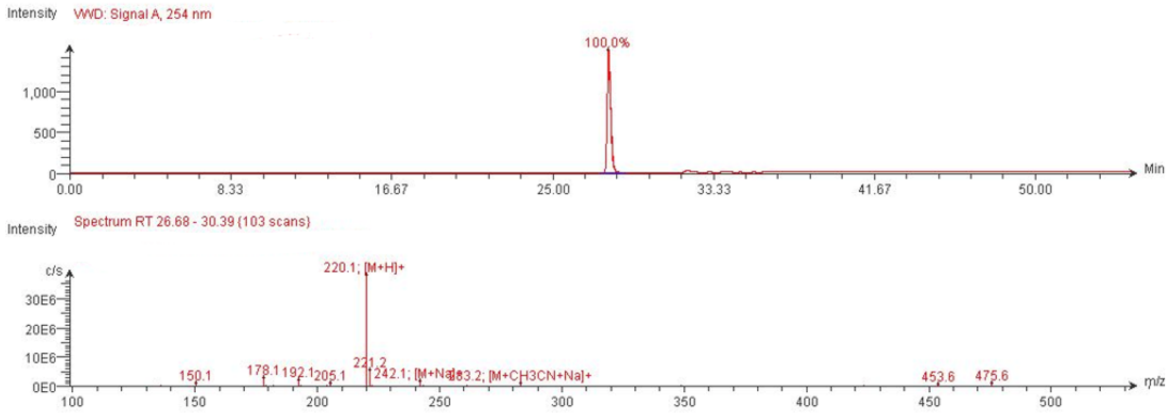


**Figure 36:** LCMS of 2,5-diisopropylpyrazolo[1,5-a]pyrimidin-7(4H)-one (**18**), representing its $(M+H)^+$ value 220.1.

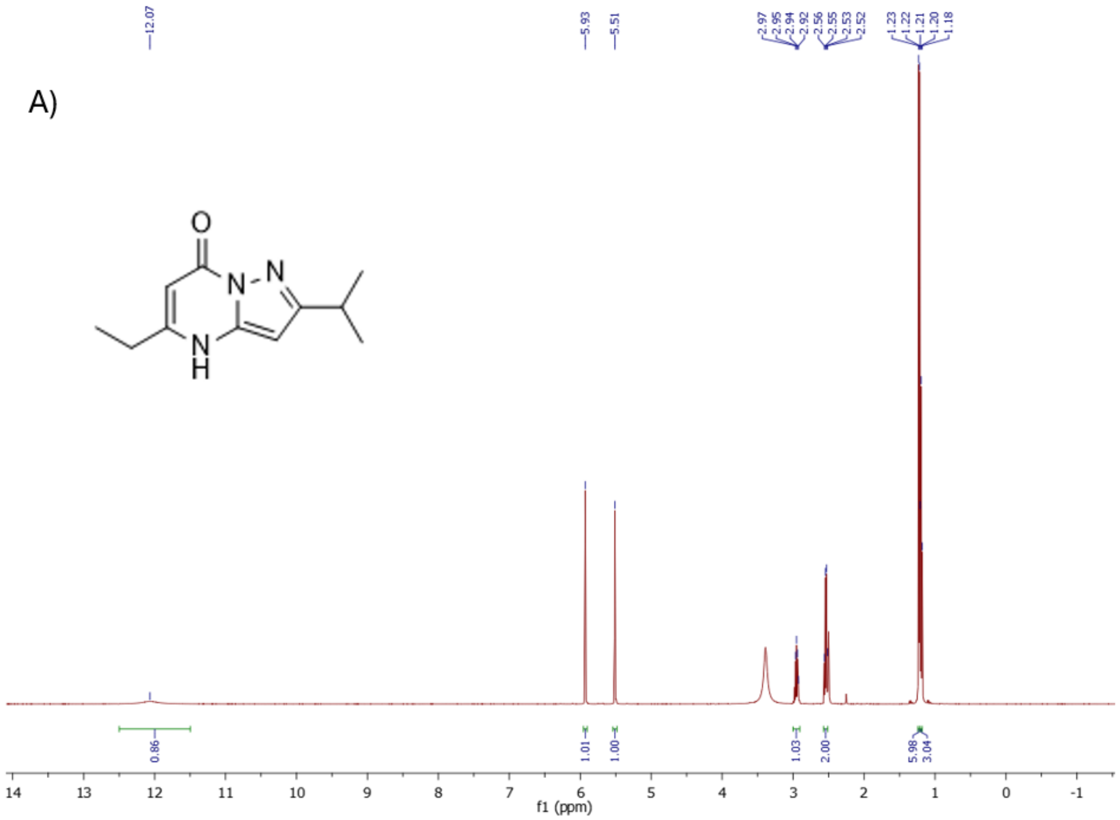


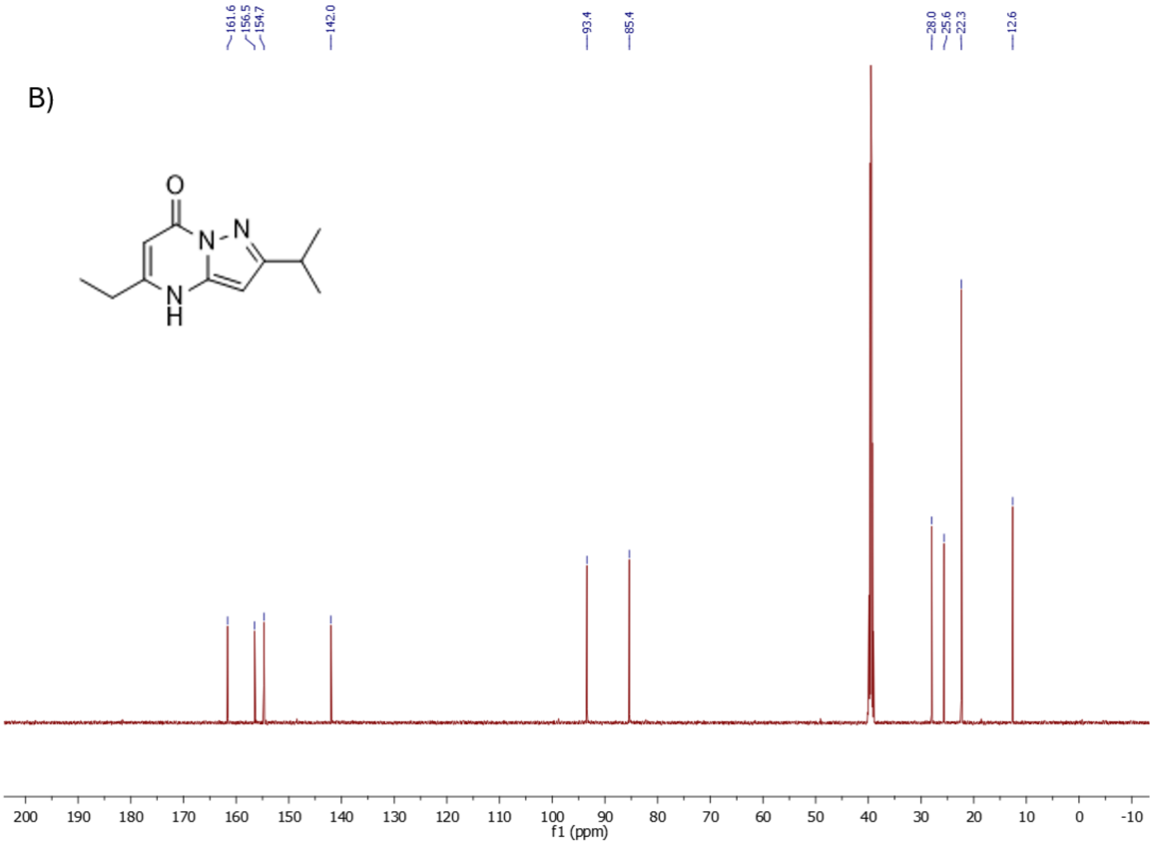


**Figure 37:** $^{1}$H (500 MHz, DMSO-$d_6$) (A) and $^{13}$C (125 MHz, DMSO-$d_6$) (B) NMR spectra of 5-ethyl-2-isopropylpyrazolo[1,5-a]pyrimidin-7(4H)-one (**19**).

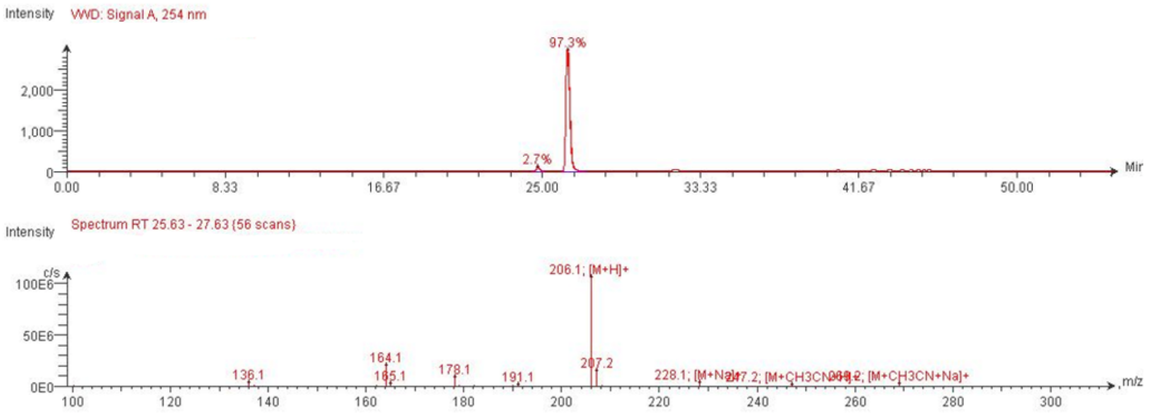


**Figure 38:** LCMS of 5-ethyl-2-isopropylpyrazolo[1,5-a]pyrimidin-7(4H)-one (**19**) representing its $(M+H)^+$ value 206.1.

A)

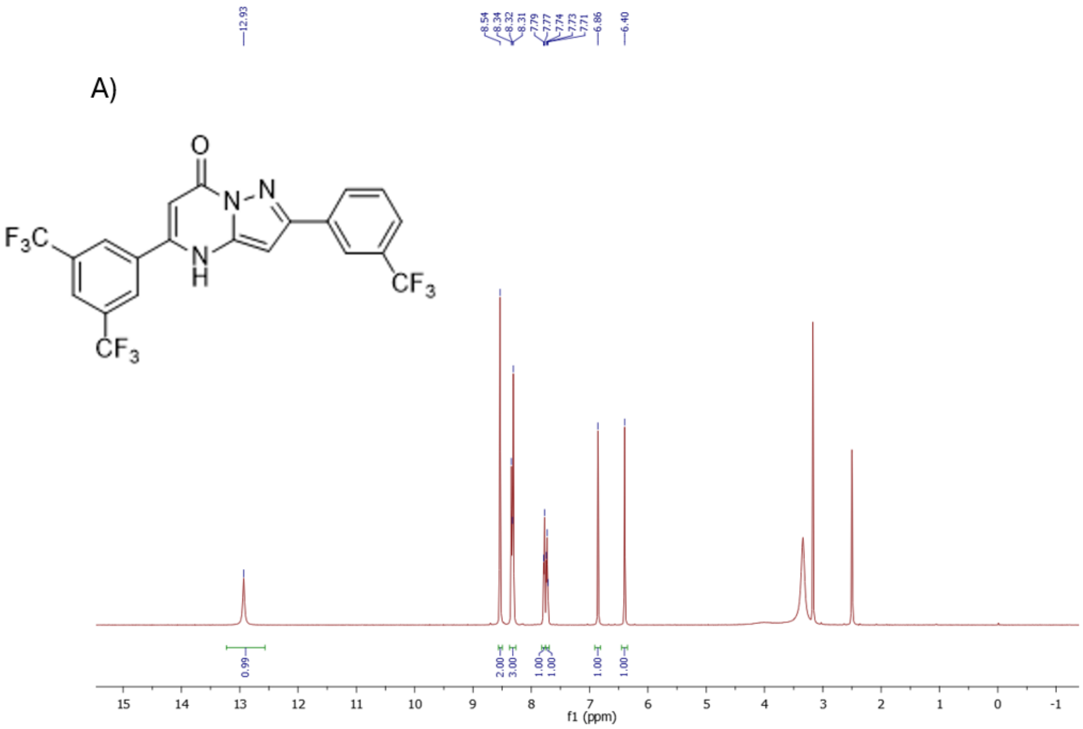
12.93
8.54
8.34
8.32
8.31
7.79
7.77
7.74
7.73
7.71
6.86
6.40
F3C
CF3
O
N
N
H
0.99
2.00
3.00
1.00
1.00
1.00
1.00
15 14 13 12 11 10 9 8 7 6 5 4 3 2 1 0 -1
f1 (ppm)

B)

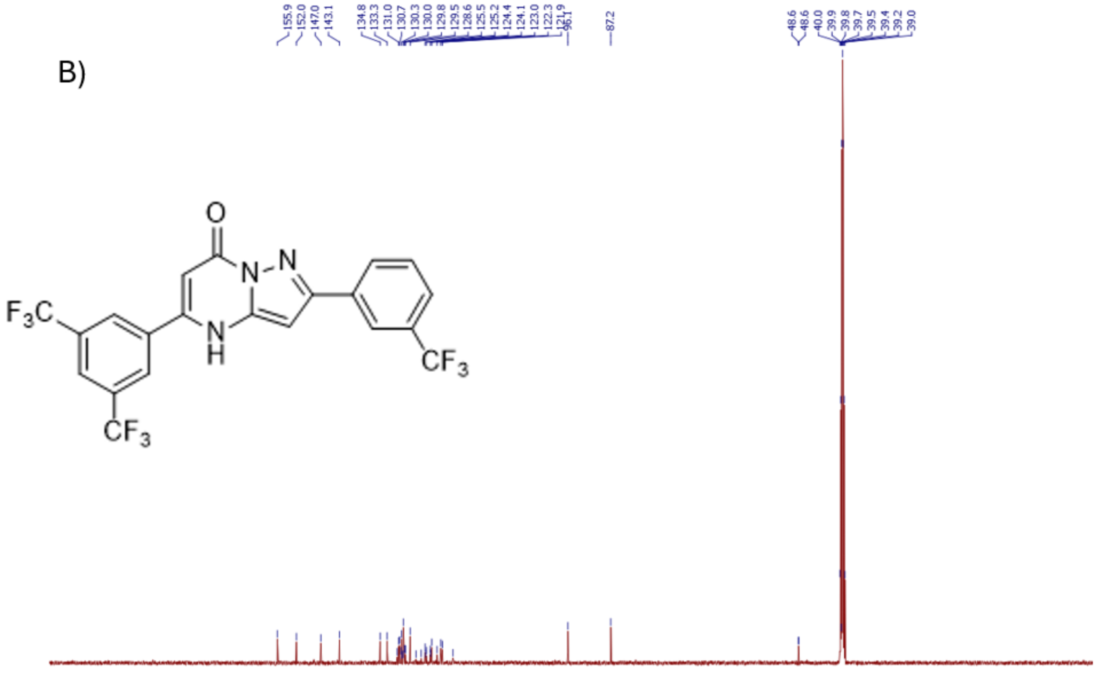
155.9
152.0
147.0
143.1
134.8
133.3
131.0
130.7
130.3
130.0
129.8
129.5
128.6
125.5
125.2
124.4
124.1
123.0
122.3
121.9
96.1
87.2
48.6
48.6
40.0
39.9
39.8
39.7
39.5
39.4
39.2
39.0
F3C
CF3
O
N
N
H
200 190 180 170 160 150 140 130 120 110 100 90 80 70 60 50 40 30 20 10 0 -10
f1 (ppm)

C)

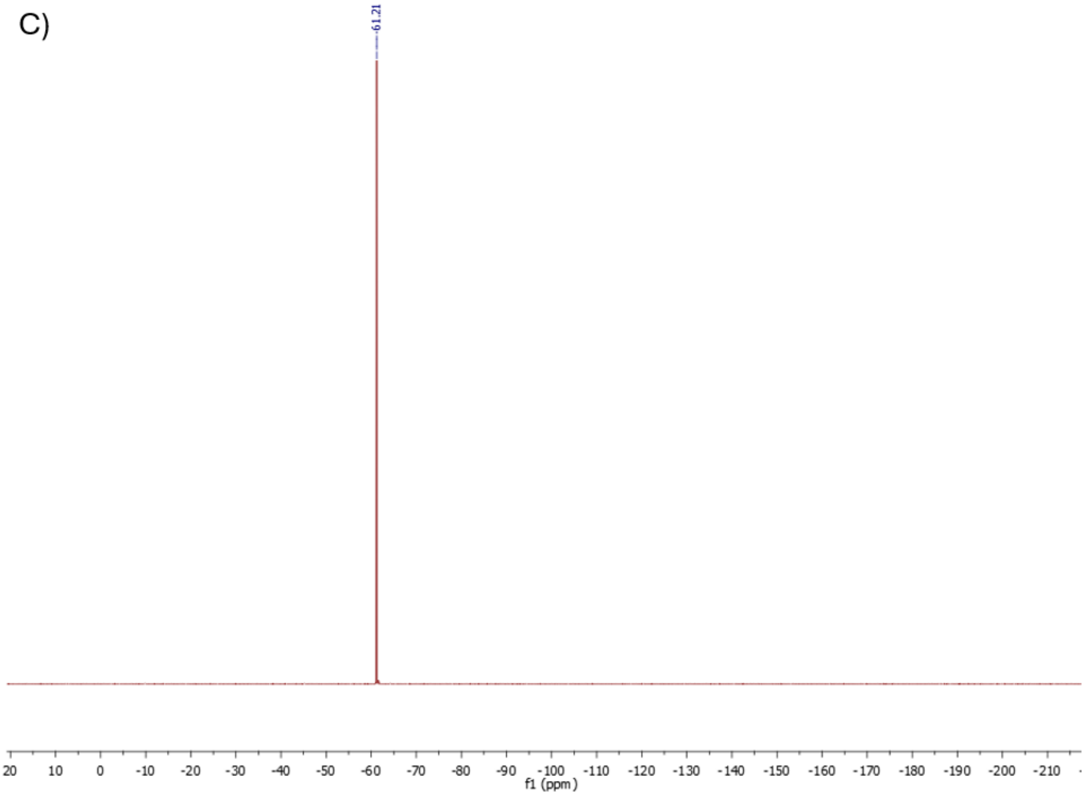


**Figure 39:** $^{1}H$ (500 MHz, DMSO-$d_6$) (A) and $^{13}C$ (125 MHz, DMSO-$d_6$) (B) $^{19}F$ (471 MHz, DMSO-$d_6$) (C) NMR spectra of 5-(3,5-bis(trifluoromethyl)phenyl)-2-(3-(trifluoromethyl)phenyl)pyrazolo[1,5-a]pyrimidin-7(4H)-one (**20**).

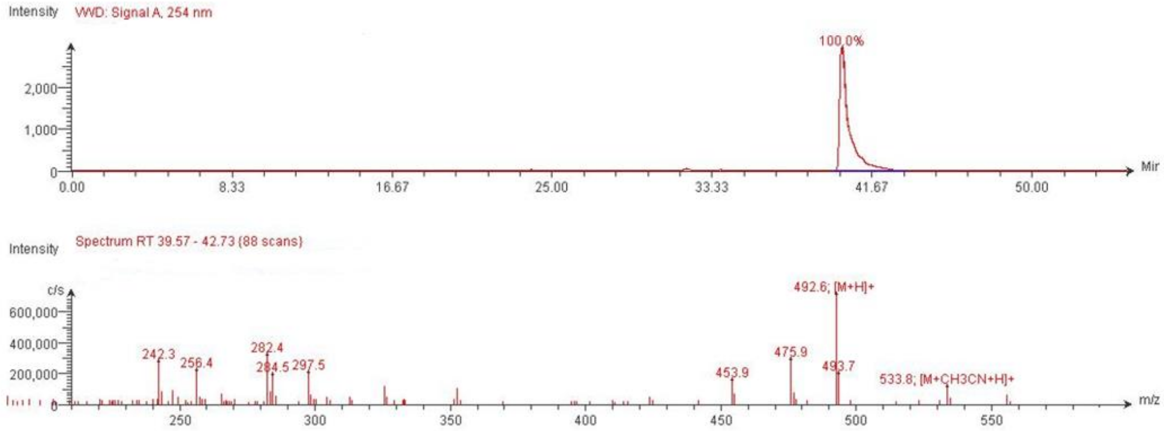


**Figure 40:** LCMS of 5-(3,5-bis(trifluoromethyl)phenyl)-2-(3-(trifluoromethyl)phenyl)pyrazolo[1,5-a]pyrimidin-7(4H)-one (**20**), representing its $(M+H)^+$ value 492.6.

A)

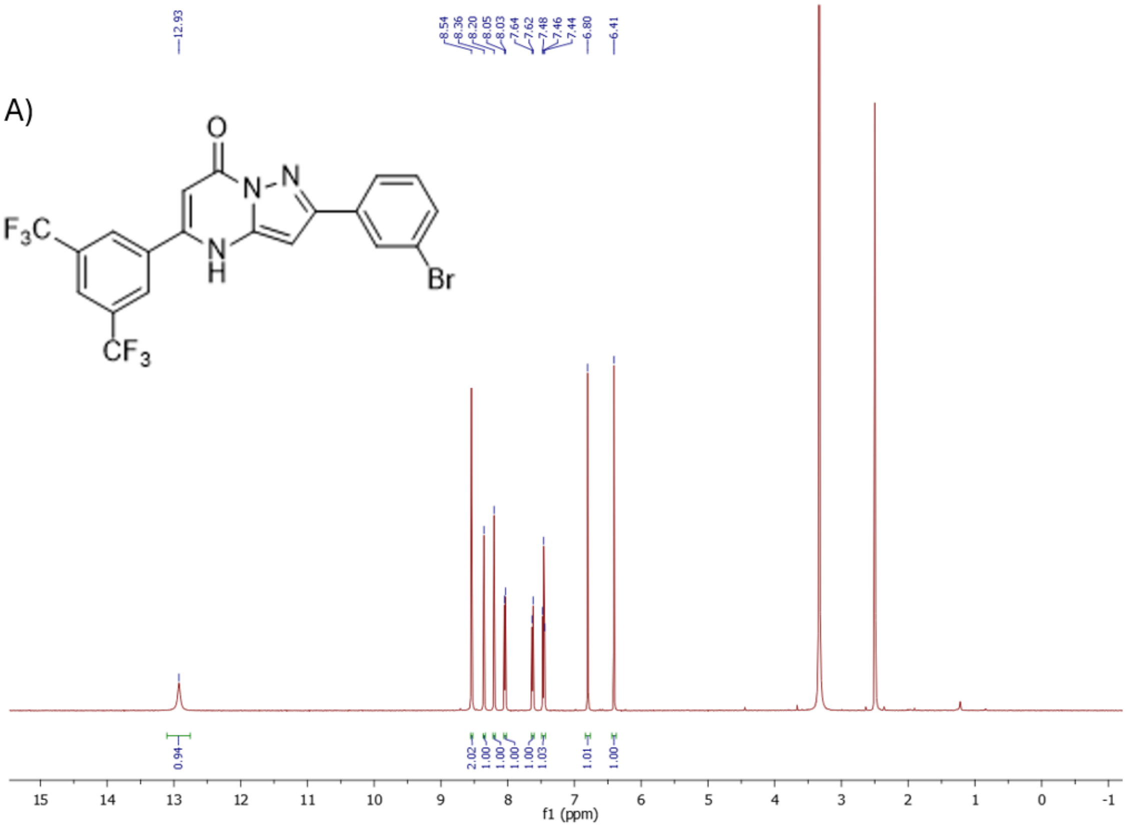
12.93
8.54
8.36
8.20
8.05
8.03
7.64
7.62
7.48
7.46
7.44
6.80
6.41
O
N
N
F3C
N
H
Br
CF3
0.94
2.02
1.00
1.00
1.00
1.00
1.03
1.01
1.00
15 14 13 12 11 10 9 8 7 6 5 4 3 2 1 0 -1
f1 (ppm)

B)

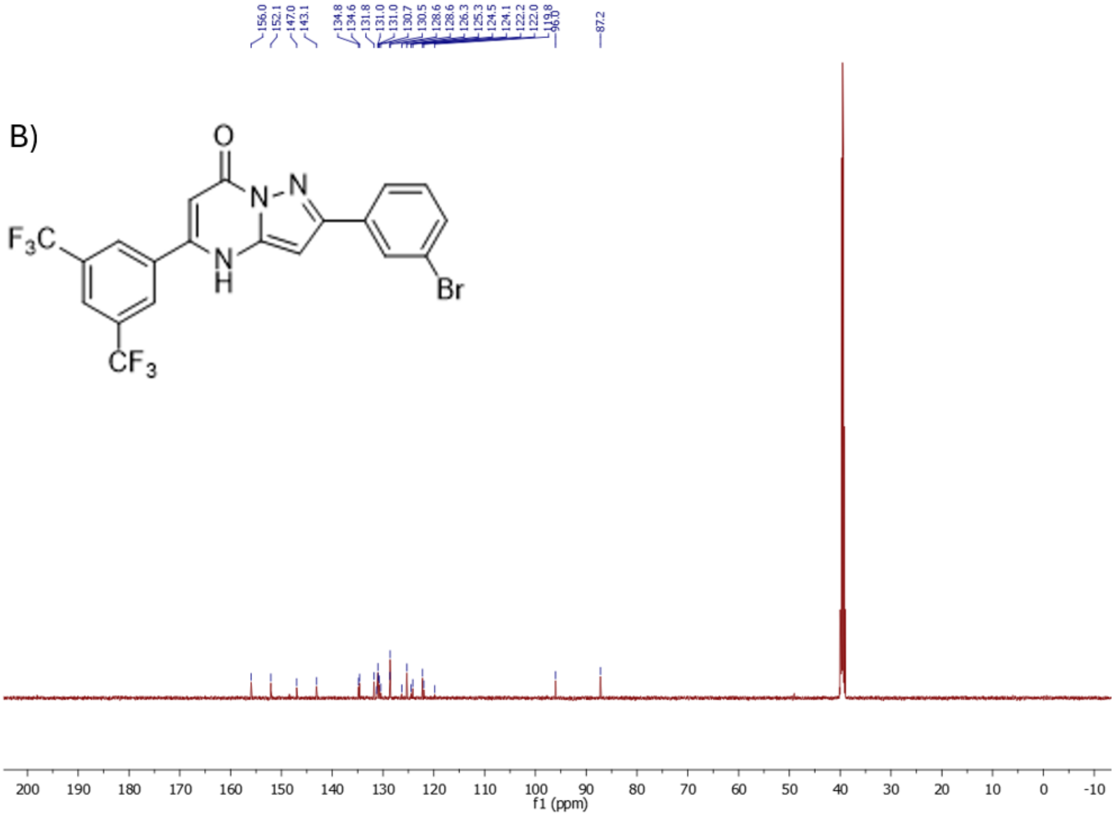
156.0
152.1
147.0
143.1
134.8
134.6
131.8
131.0
131.0
130.7
130.5
128.6
128.6
126.3
125.3
124.5
124.1
122.2
122.0
119.8
96.0
87.2
O
N
N
F3C
N
H
Br
CF3
200 190 180 170 160 150 140 130 120 110 100 90 80 70 60 50 40 30 20 10 0 -10
f1 (ppm)

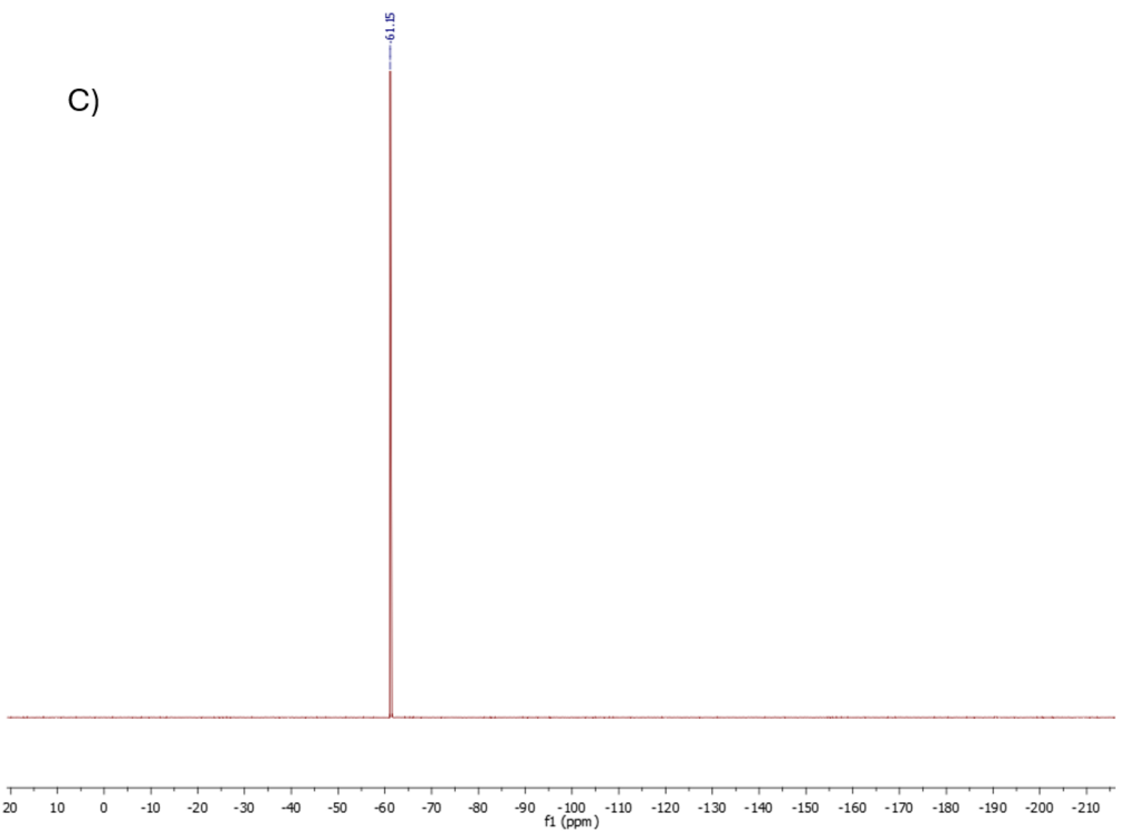


**Figure 41:** $^{1}H$ (500 MHz, DMSO-$d_6$) (A) and $^{13}C$ (125 MHz, DMSO-$d_6$) (B) $^{19}F$ (471 MHz, DMSO-$d_6$) (C) NMR spectra of 5-(3,5-bis(trifluoromethyl)phenyl)-2-(3-bromophenyl)pyrazolo[1,5-α]pyrimidin-7(4¬H)-one (**21**).

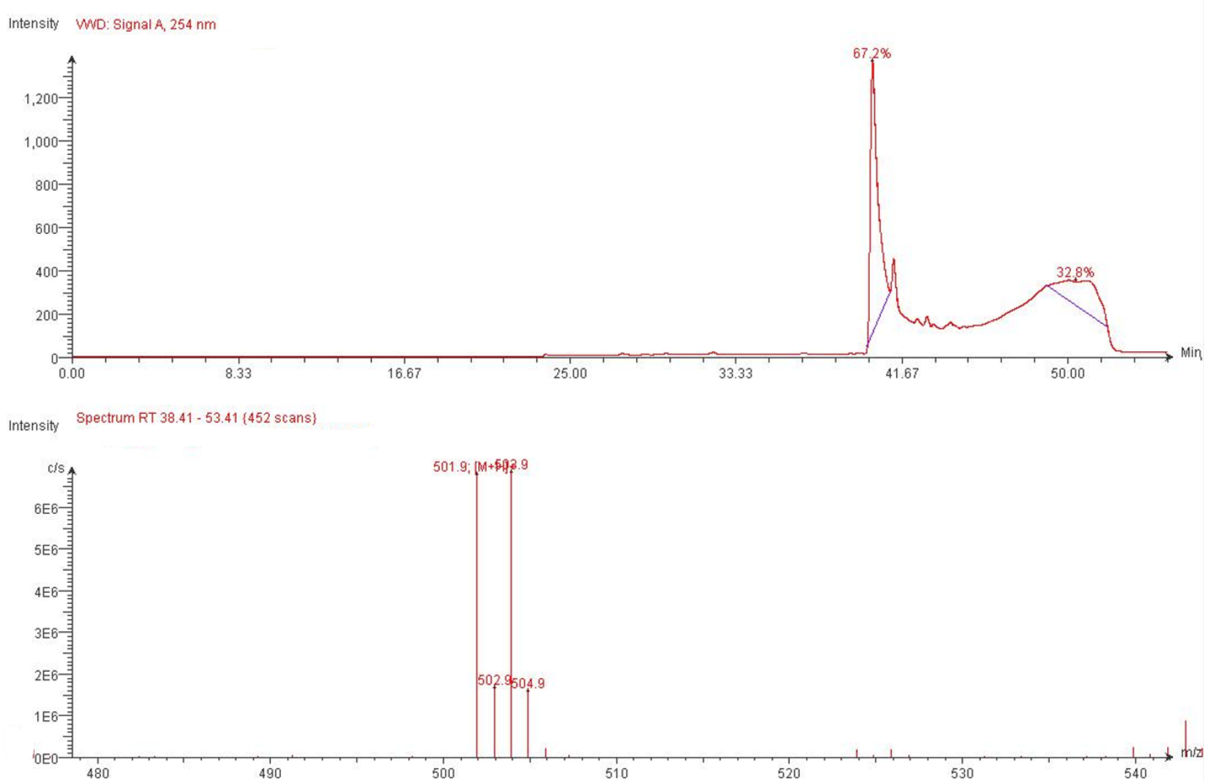


**Figure 42:** LCMS of 5-(3,5-bis(trifluoromethyl)phenyl)-2-(3-bromophenyl)pyrazolo[1,5-α]pyrimidin-7(4¬H)-one (**21**), representing its $(M+H)^{+}$ value 501.9.

A)

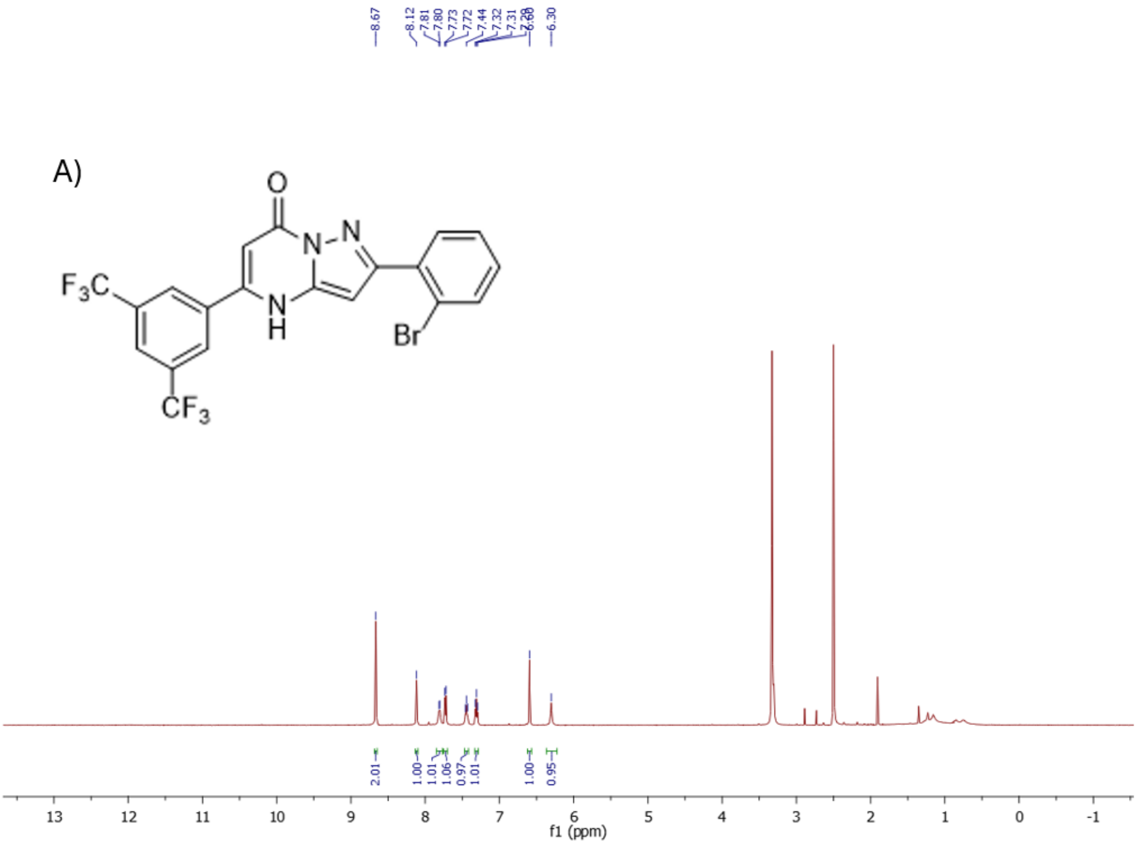
O
N
N
H
F3C
CF3
Br
8.67
8.12
2.01
1.00
1.01
1.06
0.97
1.01
1.00
0.95
f1 (ppm)

B)

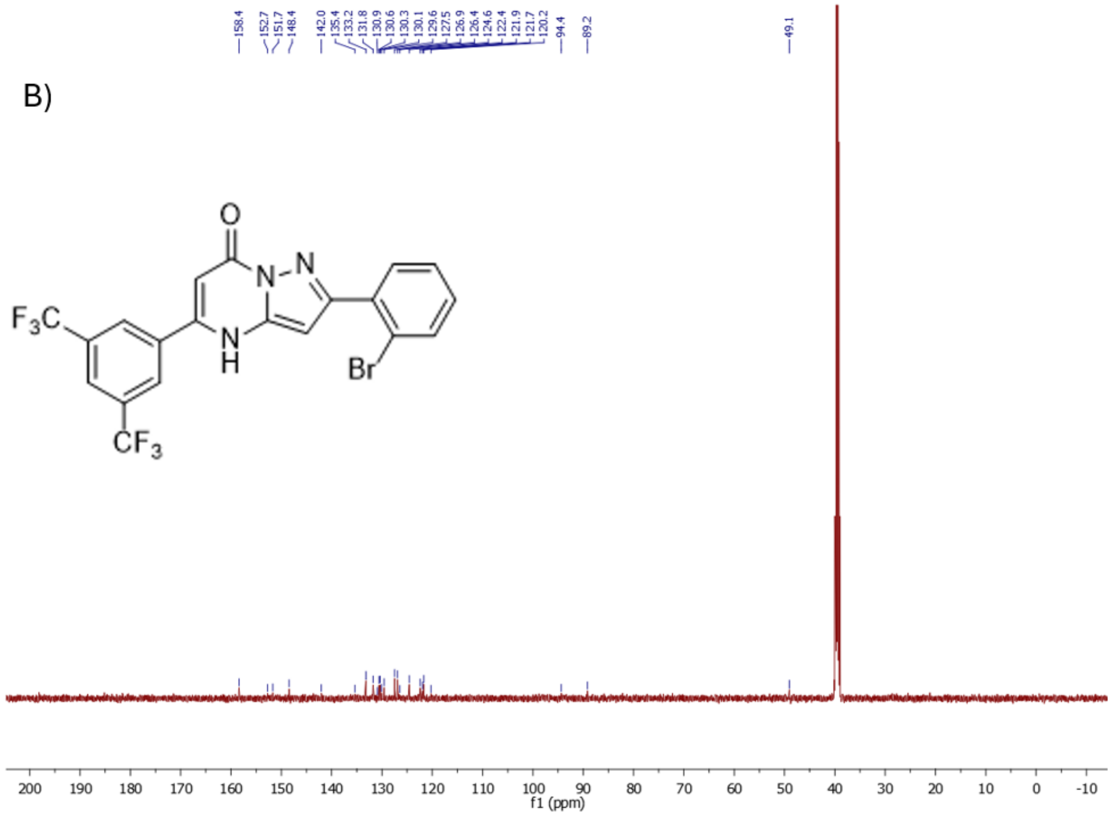
f1 (ppm)

C)

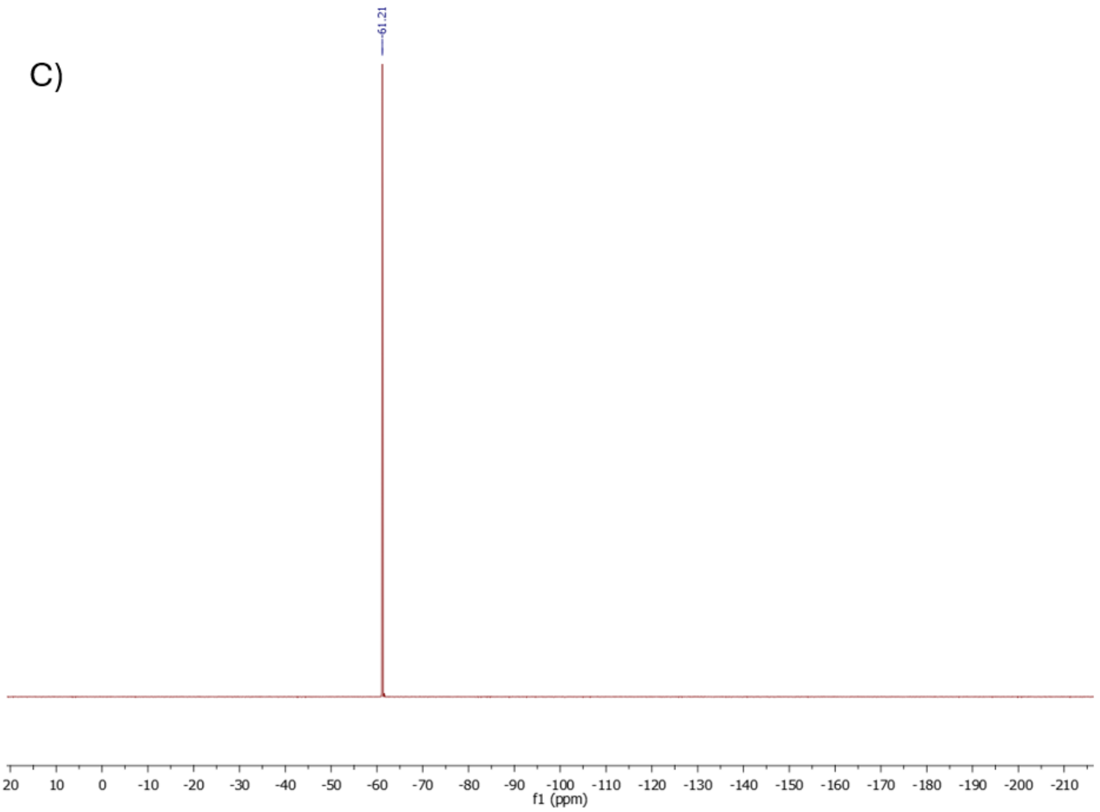


**Figure 43:** $^{1}$H (500 MHz, DMSO-*d6*) (A) and $^{13}$C (125 MHz, DMSO-*d6*) (B) $^{19}$F (471 MHz, DMSO-*d6*) (C) NMR spectra of 5-(3,5-bis(trifluoromethyl)phenyl)-2-(2-bromophenyl)pyrazolo[1,5-a]pyrimidin-7(4H)-one (**22**).

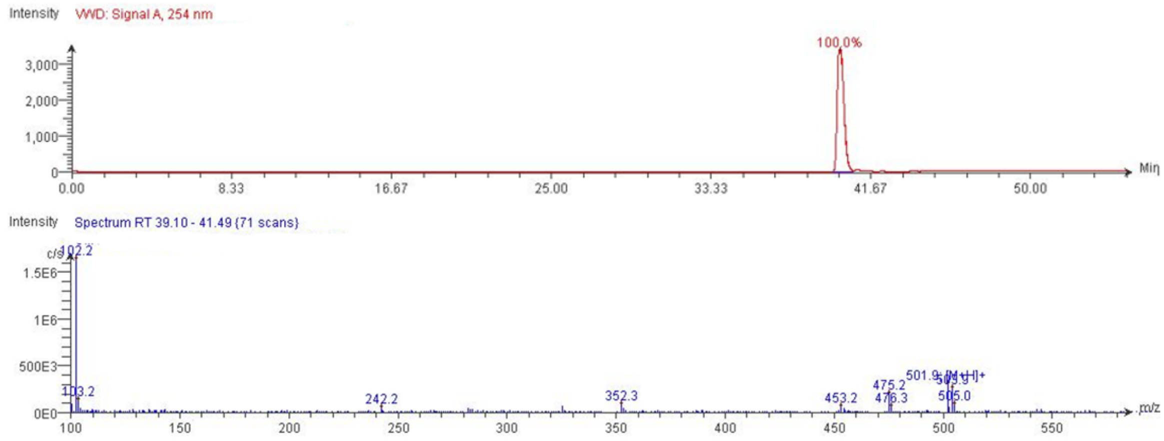


**Figure 44:** LCMS of 5-(3,5-bis(trifluoromethyl)phenyl)-2-(2-bromophenyl)pyrazolo[1,5-a]pyrimidin-7(4H)-one (**22**), representing its (M+H)[+] value 501.9.

A)

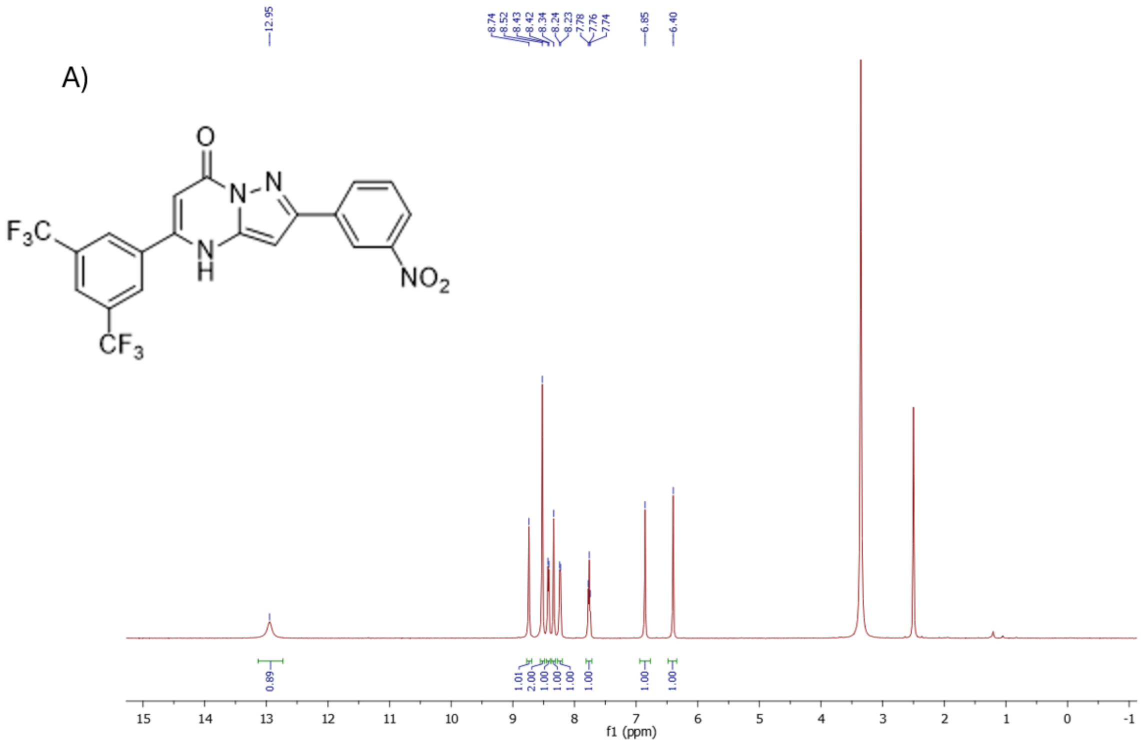

12.95
8.74
8.52
8.43
8.42
8.34
8.24
8.23
7.78
7.76
7.74
6.85
6.40
F3C
CF3
O
N
N
NH
NO2
0.89
1.01
2.00
1.00
1.00
1.00
1.00
1.00
1.00
15 14 13 12 11 10 9 8 7 6 5 4 3 2 1 0 -1
f1 (ppm)


B)

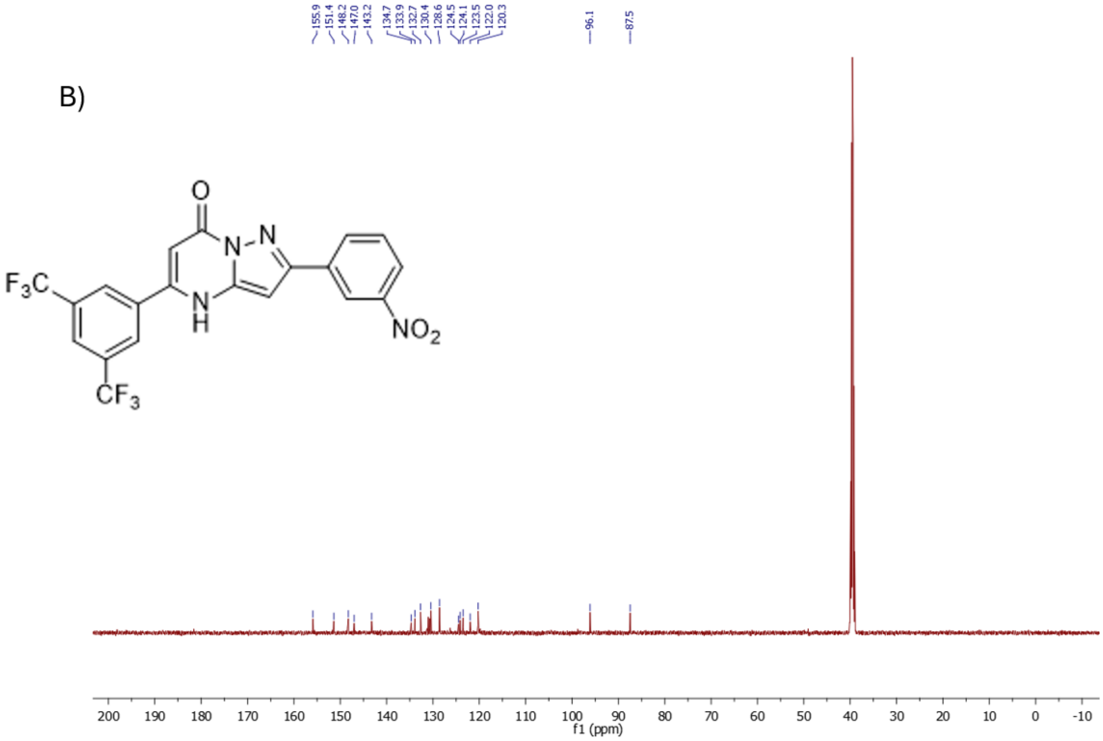

155.9
151.4
148.2
147.0
143.2
134.7
133.9
132.7
130.4
128.6
124.5
124.1
123.5
122.0
120.3
96.1
87.5
F3C
CF3
O
N
N
NH
NO2
200 190 180 170 160 150 140 130 120 110 100 90 80 70 60 50 40 30 20 10 0 -10
f1 (ppm)

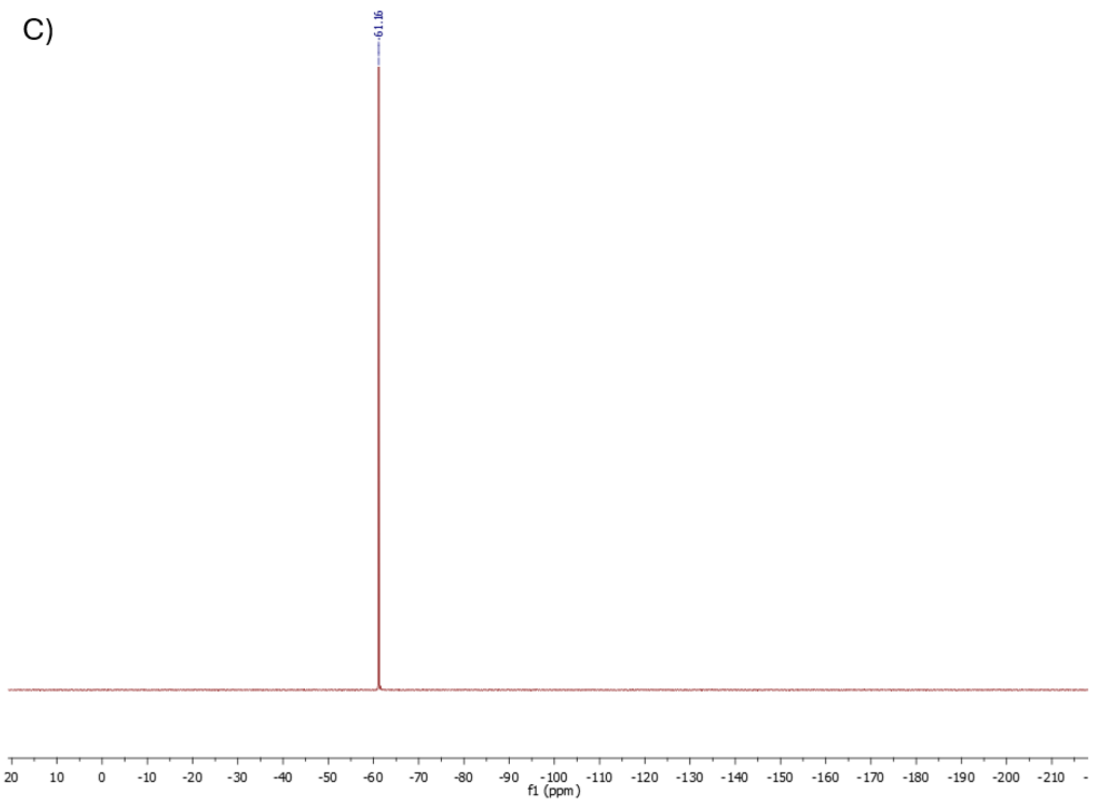

**Figure 45:** $^{1}H$ (500 MHz, DMSO-$d_6$) (A) and $^{13}C$ (125 MHz, DMSO-$d_6$) (B) $^{19}F$ (471 MHz, DMSO-$d_6$) (C) NMR spectra of 5-(3,5-bis(trifluoromethyl)phenyl)-2-(3-nitrophenyl)pyrazolo[1,5-a]pyrimidin-7(4H)-one (**23**).

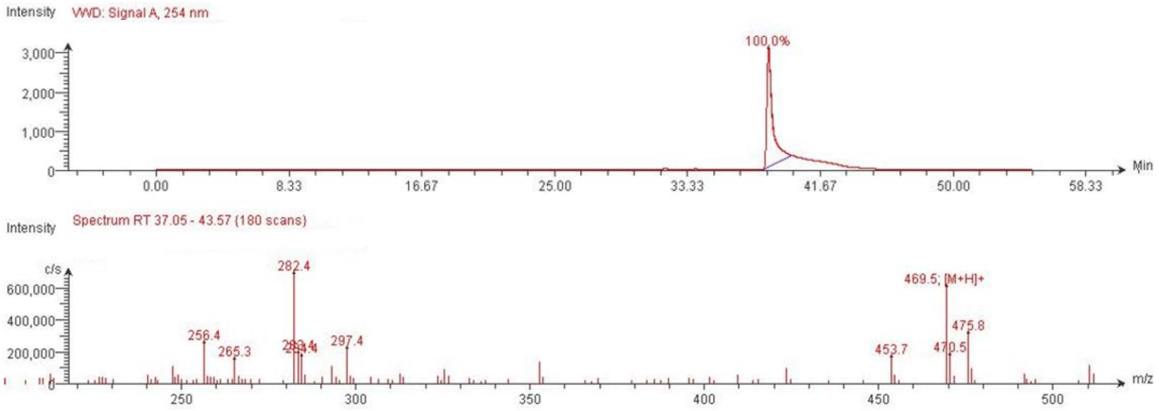

**Figure 46:** LCMS of 5-(3,5-bis(trifluoromethyl)phenyl)-2-(3-nitrophenyl)pyrazolo[1,5-a]pyrimidin-7(4H)-one (**23**), representing its $(M+H)^+$ value 469.5.

## Biology

**Table 1.** Summary of two-way ANOVA with Tukey's post-test for screened pyrazolo[1,5-α]pyrimidinone derivatives and identification of HIT compounds. Summary of results from two-way ANOVA with Tukey's post-test statistical analysis whereby the P values are reported as follows: ≥0.05 (ns), 0.01 to 0.05 (*), 0.001 to 0.01 (**), 0.001 to 0.01(***) and <0.0001(****). The HIT compounds which passed the statistical testing and criteria thresholds have been highlighted in grey.

| Compound | $R^1$ Group | $R^2$ Group | U-251 MG $IC_{50}$ Value (μM) | HEK293 $IC_{50}$ Value (μM) | Statistical Summary | P value | All criteria met -Yes/No |
|---|---|---|---|---|---|---|---|
| **1** | $CH_3$ | 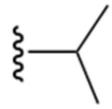 | 118.6 | 449.6 | * | 0.0141 | No |
| **2** | 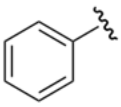 | 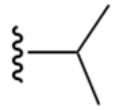 | 20.60 | 9.802 | * | 0.0176 | No |
| **3** | 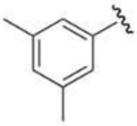 | 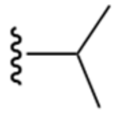 | 9.289 | 6.517 | *** | 0.0009 | No |
| **4** | 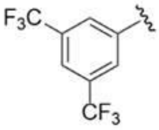 | 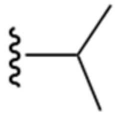 | 14.02 | 7.383 | *** | 0.0009 | No |
| **5** | 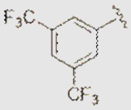 | Ph | 6.172 | 15.35 | **** | <0.0001 | Yes |
| **6** | 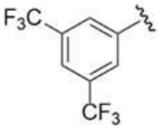 | 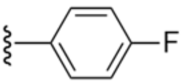 | 52.18 | 68.22 | ** | 0.0028 | No |
| **7** | 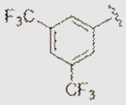 | 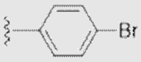 | 29.82 | 60.88 | **** | <0.0001 | Yes |
| **8** | 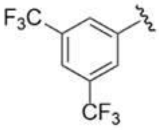 | 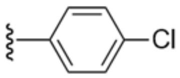 | 17.30 | 52.37 | **** | <0.0001 | No |

| | | | | | | | |
|---|---|---|---|---|---|---|---|
| **9** | 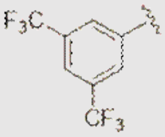 | 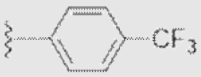 | 27.46 | 54.43 | **** | <0.0001 | Yes |
| **10** | 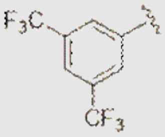 | 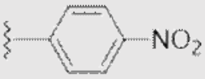 | 29.62 | 73.78 | **** | <0.0001 | Yes |
| **11** | 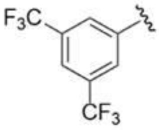 | 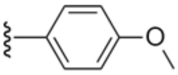 | 23.48 | 29.48 | **** | <0.0001 | No |
| **12** | 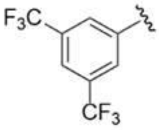 | 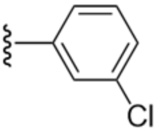 | 21.65 | 29.48 | **** | <0.0001 | No |
| **13** | 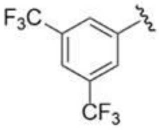 | 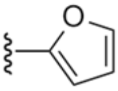 | N/A | N/A | Ns | 0.7979 | No |
| **14** | $CH_3$ | Ph | 81.49 | N/A | ** | 0.0025 | No |
| **15** | Ph | Ph | 19.92 | 15.92 | **** | <0.0001 | No |
| **16** | $CH_3$ | $CH_3$ | N/A | N/A | **** | <0.0001 | No |
| **17** | Ph | t-Bu | 76.58 | N/A | **** | <0.0001 | No |
| **18** | i-Pr | i-Pr | 170.0 | 117.7 | * | 0.0313 | No |
| **19** | Et | i-Pr | 154.0 | N/A | Ns | 0.9381 | No |
| **20** | 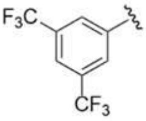 | 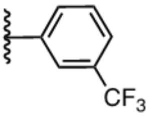 | 30.97 | N/A | ** | 0.0012 | No |
| **21** | 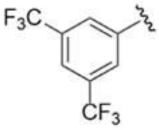 | 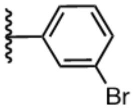 | 0.7139 | 19.04 | **** | <0.0001 | Yes |
| **22** | 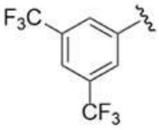 | 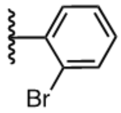 | 2.786 | 66.91 | **** | <0.0001 | Yes |

| | | | | | | | |
|---|---|---|---|---|---|---|---|
| **23** | 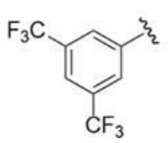 | 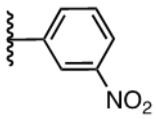 | 23.19 | 60.32 | **** | <0.0001 | Yes |

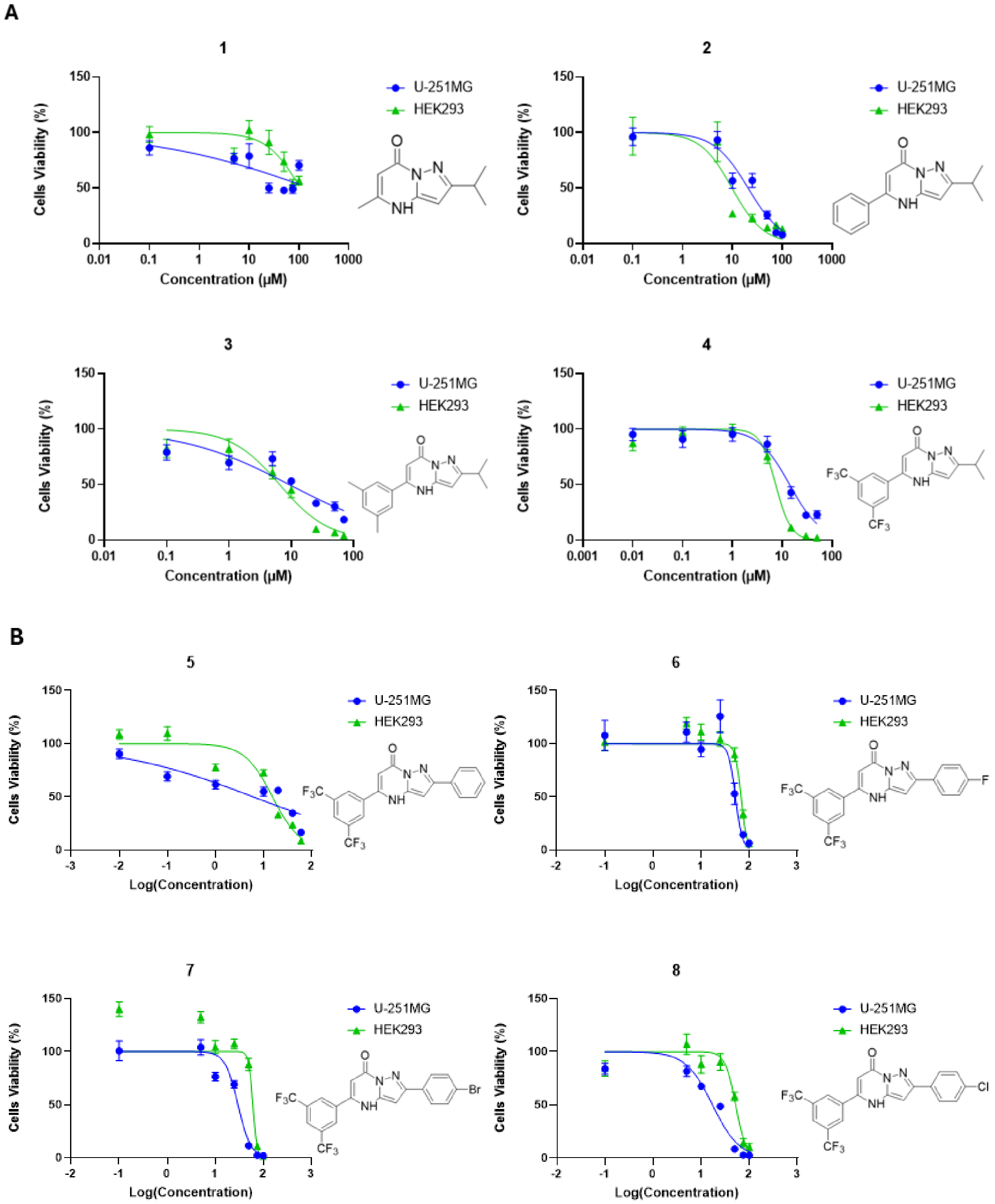

**9**

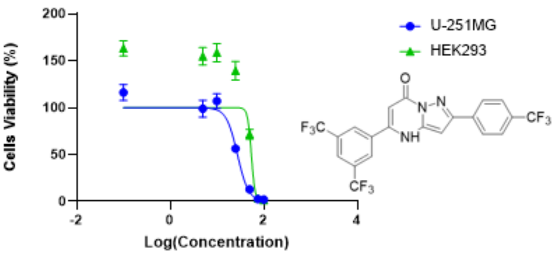


**10**

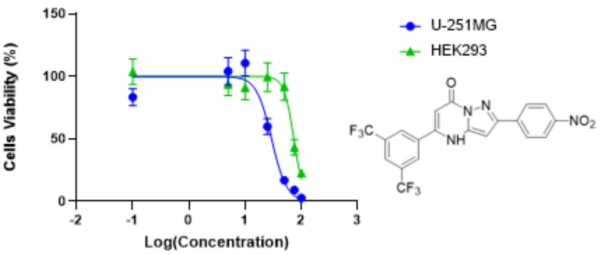


**11**

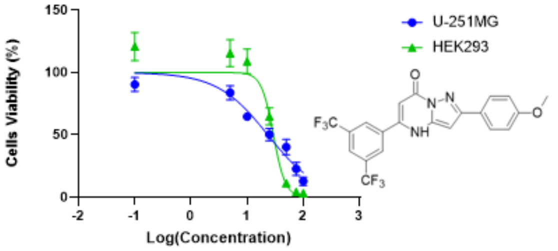


**12**

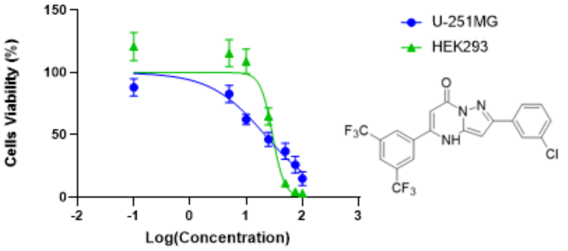


**13**

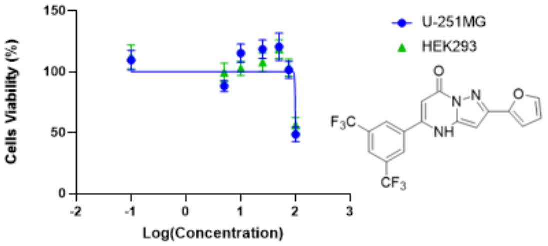


**C**

**14**

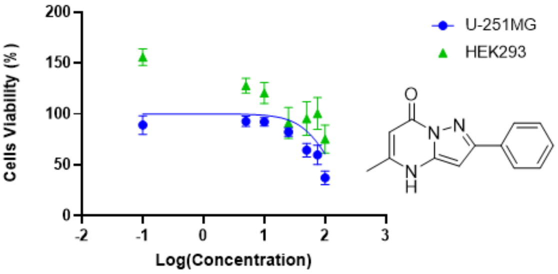


**15**

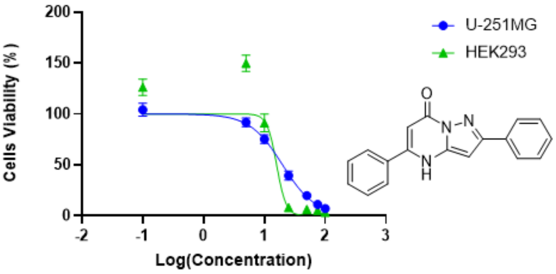

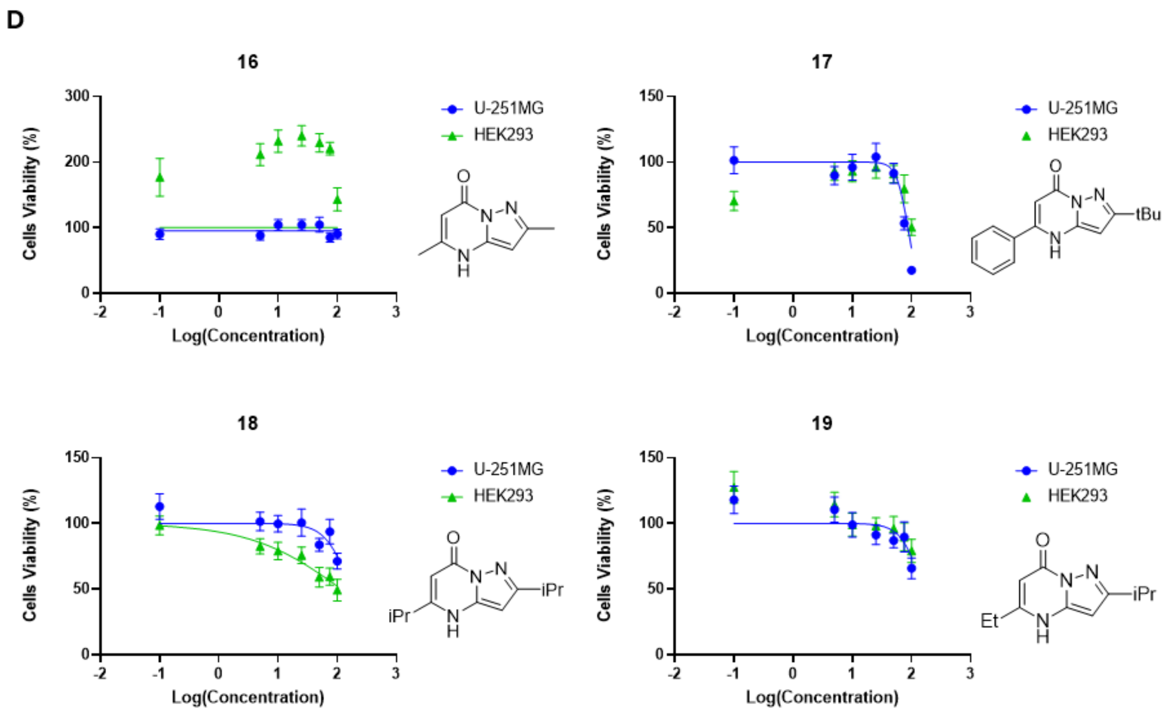


**Figure 47.** Dose-response curves for pyrazolo[1,5-α]pyrimidinones. U-251MG glioblastoma and HEK293 embryonic kidney cell lines were treated with varied concentrations of **A.** Group 1, **B.** Group 2, **C.** Group 3, or **D.** Group 4 pyrazolo[1,5-α]pyrimidinone derivatives and incubated for 5 days. Post incubation the MTT cell viability assay was carried out to obtain dose response curves, where the X axis denotes the logarithmic concentration of compound, and the Y axis denote the % cell viability of the pooled data for the compounds.

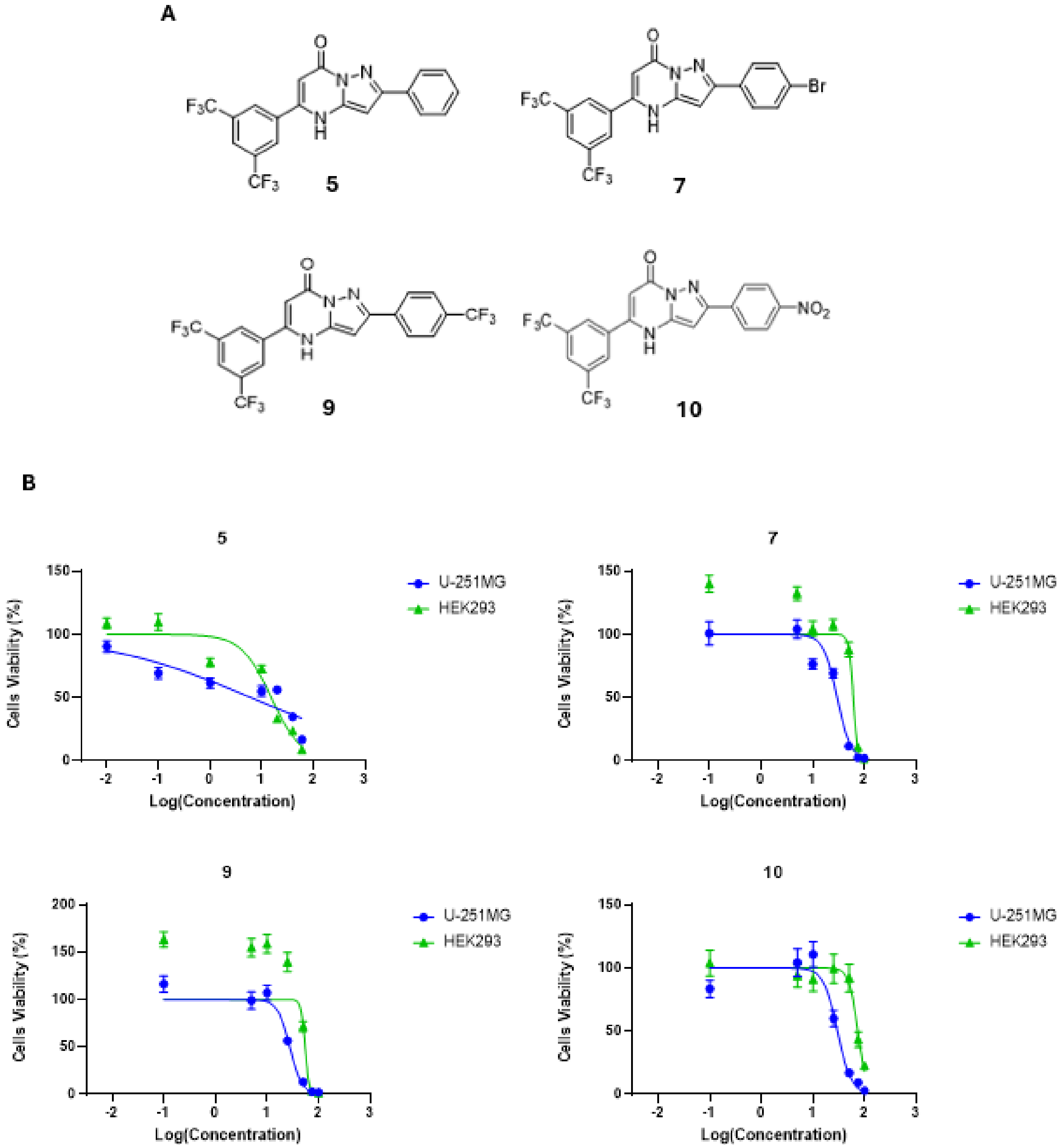


**Figure 48.** Structure of HIT compounds and its effect on the cytotoxicity of pyrazolo[1,5-α]pyrimidinone derivatives. **A.** Structures of preliminary HIT compounds chosen for further analysis and HIT optimization. **B.** **5** exhibited a biological response in both U-251 MG and HEK293 cell lines, in addition to demonstrating selective cytotoxicity towards glioblastoma cells over non-cancerous cells, evident by 2 separate curves (p-value <0.0001). Addition of lipophilic, electron-withdrawing groups i.e., $NO_2$, $CF_3$, and Br resulted in an improved selective capacity with no biological response in HEK293 cells but significant cytotoxicity in glioblastoma cells (p-value <0.0001).

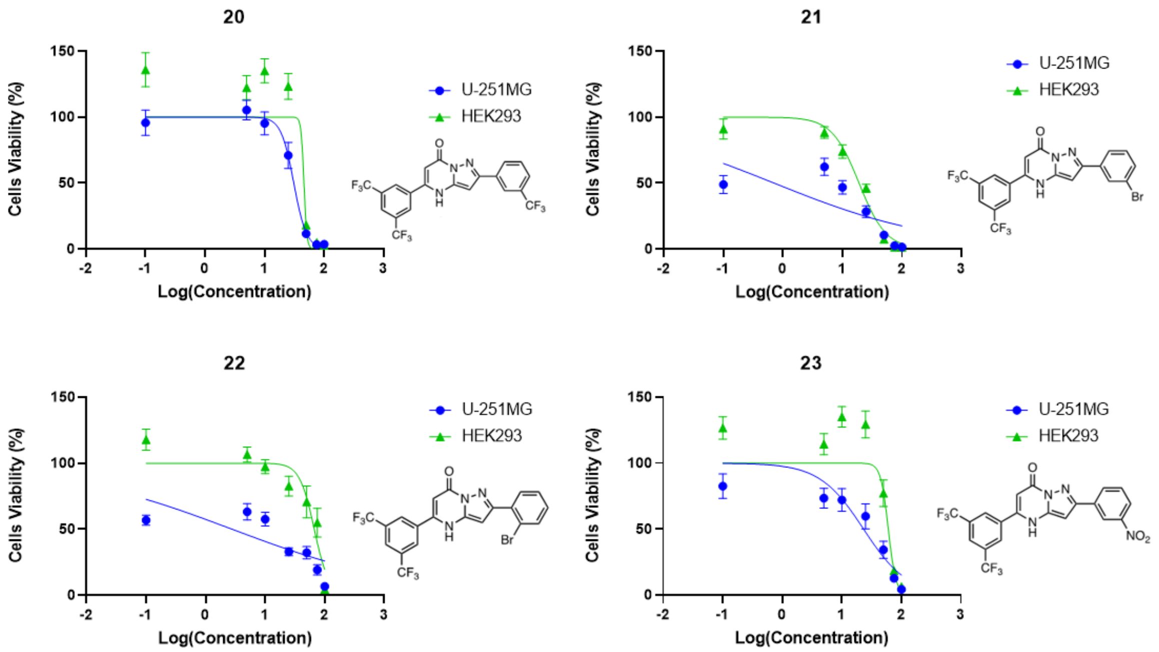


**Figure 49**. Dose response curves of optimised Group 2 pyrazolo[1,5-α]pyrimidinone derivatives. U-251 MG glioblastoma and HEK293 embryonic kidney cell lines were treated with optimised Group 2 pyrazolo[1,5-α]pyrimidinone derivatives for 5 days. Post incubation the MTT cell viability assay was carried out to obtain dose response curves, where the X axis denotes the logarithmic concentration of compound, and the Y axis denote the % cell viability of the pooled data for the optimised Group 2 compounds.

**Table 2.** HIT compound concentrations for flow cytometry PI and JC-1 analysis. U-251 MG cells were treated with the below mentioned concentrations of the HIT compounds including the highest toxic concentrations, the $IC_{50}$ value and the highest non-toxic drug concentration, prior to evaluation with either PI stain or JC-1 dye.

| Compound Name | Highest Toxic Concentration (µM) | $IC_{50}$ Value (µM) | Highest Non-Toxic Concentration (HNT) (µM) |
|---|---|---|---|
| **5** | 45 | 6.17 | 0.1 |
| **7** | 50 | 29.49 | 1 |
| **9** | 50 | 26.75 | 10 |
| **10** | 50 | 29.62 | 5 |

| | | | |
|---|---|---|---|
| **21** | 50 | 0.66 | 1 |
| **22** | 50 | 2.79 | 1 |

**Table 3.** Summary of results from one-way ANOVA statistical analysis for PI evaluated HIT and optimised HIT compounds: **7**, **9, 10**, **21** and **22,** whereby the p values are reported as follows: ≥0.05 (ns), 0.01 to 0.05 (*), 0.001 to 0.01 (**), 0.001 to 0.01(***) and <0.0001(****). The negative control was compared with the highest toxic concentration, $IC_{50}$ value and the lowest toxic concentration using Dunnett's T3 multiple comparisons statistical test.

| HIT Compound | Comparison | Summary | P Value |
|---|---|---|---|
| **7** | Neg Ctrl v $IC_{50}$ | *** | 0.0005 |
| | Neg Ctrl v 50 μM | ** | 0.0034 |
| | Neg Ctrl v HNT | * | 0.0216 |
| **9** | Neg Ctrl v $IC_{50}$ | ** | 0.084 |
| | Neg Ctrl v 50 μM | **** | <0.001 |
| | Neg Ctrl v HNT | ns | >0.9999 |
| **10** | Neg Ctrl v $IC_{50}$ | ** | 0.0048 |
| | Neg Ctrl v 50 μM | *** | 0.0005 |
| | Neg Ctrl v HNT | ns | 0.1976 |
| **21** | Neg Ctrl v $IC_{50}$ | * | 0.0327 |
| | Neg Ctrl v 50 μM | **** | <0.001 |
| | Neg Ctrl v HNT | ns | 0.0979 |
| **22** | Neg Ctrl v $IC_{50}$ | *** | 0.0002 |
| | Neg Ctrl v 50 μM | **** | <0.0001 |
| | Neg Ctrl v HNT | ** | 0.0012 |

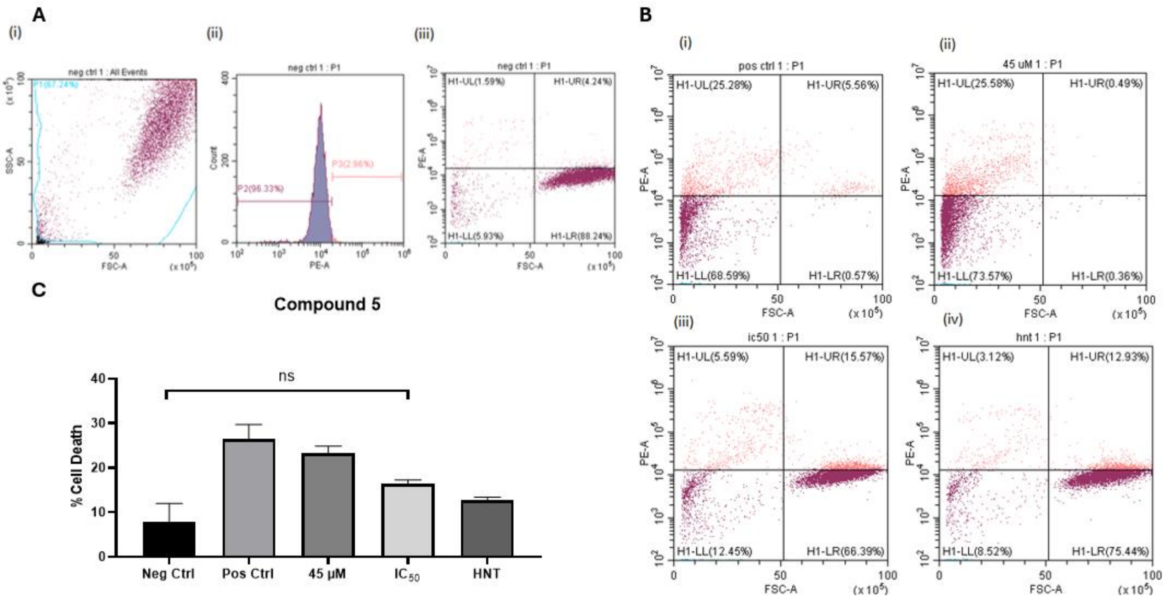


**Figure 50.** Example of PI flow cytometry analysis. **A.** U-251 MG cells treated with **5** for 5 days were stained with PI and analysed via flow cytometry. (i) Graph showing total cell population excluding cellular debris. (ii) Graph distinguishing the viable cell population P2 from the dead cell population P3. (iii) Quadrant graph for cell cycle analysis for **5. B.** Quadrant graphs of forward scatter (FSC-A) verses PE-A of U-251 MG cells treated with (i) positive control (ii) highest toxic concentration of **5** (iii) **5** $IC_{50}$ concentration (iv) highest non-toxic concentration of **5. C.** Bar chart representation of percentage cell death post 5-day incubation with varied concentrations of **5** determined via PI flow cytometric analysis. Statistical significance calculated via one-way ANOVA with Dunnett's T3 multiple comparison only shown between the $IC_{50}$ value of **5** and negative control.

**Table 4.** Summary of results from one-way ANOVA statistical analysis for JC-1 evaluated HIT and optimised HIT compounds: **7, 9, 10, 21** and **22,** whereby the P values are reported as follows: ≥0.05 (ns), 0.01 to 0.05 (*), 0.001 to 0.01 (**), 0.001 to 0.01(***) and <0.0001(****). The negative control was compared with the highest toxic concentration, $IC_{50}$ value and the lowest toxic concentration using Dunnett's T3 multiple comparisons statistical test.

| HIT Compound | Comparison | Summary | P Value |
|---|---|---|---|
| **7** | Neg Ctrl v $IC_{50}$ | ** | 0.0021 |
| | Neg Ctrl v 50 µM | **** | <0.0001 |
| | Neg Ctrl v HNT | ns | 0.9867 |
| **9** | Neg Ctrl v $IC_{50}$ | *** | 0.0007 |
| | Neg Ctrl v 50 µM | ** | 0.0028 |
| | Neg Ctrl v HNT | ns | 0.2912 |

| | | | |
|---|---|---|---|
| **10** | Neg Ctrl v $IC_{50}$ | ** | 0.0075 |
| | Neg Ctrl v 50 µM | *** | 0.0004 |
| | Neg Ctrl v HNT | * | 0.0191 |
| **21** | Neg Ctrl v $IC_{50}$ | * | 0.0408 |
| | Neg Ctrl v 50 µM | *** | 0.0006 |
| | Neg Ctrl v HNT | *** | 0.0002 |
| **22** | Neg Ctrl v $IC_{50}$ | * | 0.0408 |
| | Neg Ctrl v 50 µM | * | 0.0116 |
| | Neg Ctrl v HNT | ns | 0.2188 |

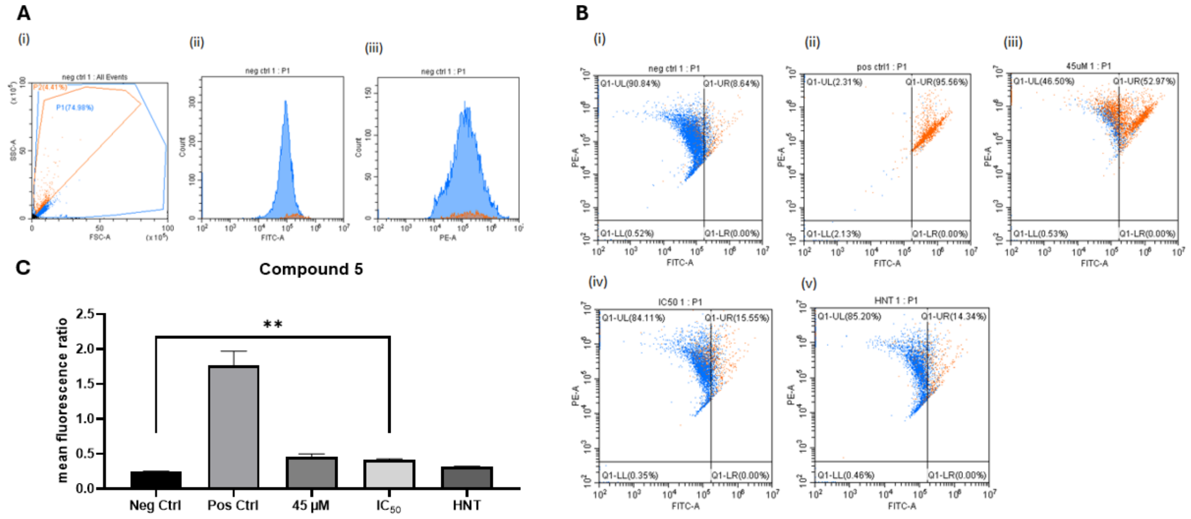


**Figure 51**. Example of JC-1 flow cytometry analysis**. A.** U-251 MG cells treated with **5** for 5 days were stained with JC-1 and analysed via flow cytometry. (i) Graph showing total cell population excluding cellular debris. (ii) Graph distinguishing the cell population in the green FITC channel (iii) Graph distinguishing the cell population in the red PE channel **B.** Quadrant graphs of FITC-A verses PE-A of U-251 MG cells treated with varied concentrations of **5.** (i) 8.64 % apoptotic cell population observed in negative control for **5**. (ii) 95.56 % apoptotic cell population observed in positive control for **5** (iii) 52.97 % apoptosis observed in highest toxic concentration of **5** (iv) 15.55 % apoptosis observed in **5** $IC_{50}$ (v) 14.34 % apoptosis observed in highest non-toxic concentration of **5. C.** Bar chart representation of percentage cell death post 5-day incubation with varied concentrations of **5** determined via JC-1 flow cytometric analysis. Statistical significance calculated via one-way ANOVA with Dunnett's T3 multiple comparison only shown between the $IC_{50}$ value of 5 and negative control.
